# Copper(II) Complexes with 2,2′:6′,2″-Terpyridine Derivatives Displaying Dimeric Dichloro−μ–Bridged Crystal Structure: Biological Activities from 2D and 3D Tumor Spheroids to In Vivo Models

**DOI:** 10.1021/acs.jmedchem.4c00119

**Published:** 2024-03-22

**Authors:** Katarzyna Choroba, Barbara Machura, Karol Erfurt, Ana Rita Casimiro, Sandra Cordeiro, Pedro V. Baptista, Alexandra R. Fernandes

**Affiliations:** † Institute of Chemistry, University of Silesia, Szkolna 9, 40-006 Katowice, Poland; ‡ Department of Chemical Organic Technology and Petrochemistry, Silesian University of Technology, Krzywoustego 4, 44-100 Gliwice, Poland; § Associate Laboratory i4HB-Institute for Health and Bioeconomy, NOVA School of Science and Technology, NOVA University Lisbon, 2819-516 Caparica, Portugal; ∥ UCIBIO, Departamento de Ciências da Vida, NOVA School of Science and Technology, Campus de Caparica, 2829-516 Caparica, Portugal

## Abstract

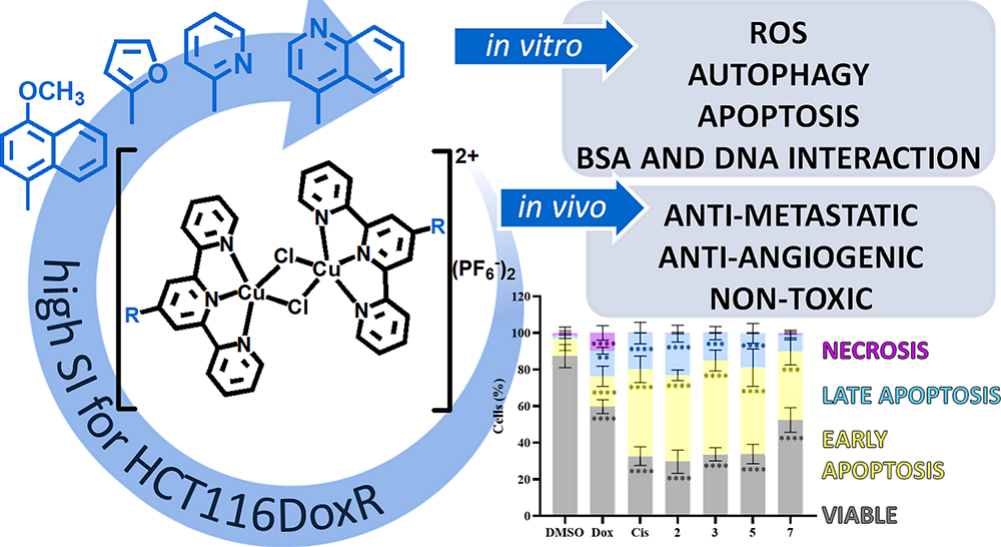

Eight 2,2′:6′,2″-terpyridines, substituted at the 4′-position with aromatic groups featuring variations in π-conjugation, ring size, heteroatoms, and methoxy groups, were employed to enhance the antiproliferative potential of [Cu_2_Cl_2_(R-terpy)_2_](PF_6_)_2_. Assessing the cytotoxicity in A2780 (ovarian carcinoma), HCT116 (colorectal carcinoma), and HCT116DoxR (colorectal carcinoma resistant to doxorubicin) and normal primary fibroblasts revealed that Cu(II) complexes with 4-quinolinyl, 4-methoxy-1-naphthyl, 2-furanyl, and 2-pyridynyl substituents showed superior therapeutic potential in HCT116DoxR cells with significantly reduced cytotoxicity in normal fibroblasts (42–129× lower). Besides their cytotoxicity, the Cu(II) complexes are able to increase intracellular ROS and interfere with cell cycle progression, leading to cell death by apoptosis and autophagy. Importantly, they demonstrated antimetastatic and antiangiogenic properties without in vivo toxicity. In accordance with their nuclear accumulation, the Cu(II) complexes are able to cleave pDNA and interact with bovine serum albumin, which is a good indication of their ability for internalization and transport toward tumor cells.

## Introduction

Copper ranks as the third most abundant transition metal in the human body, playing a crucial role in a wide spectrum of biological processes, such as electron transfer, oxidation, and dioxygen transport.^1,2^ Cu(I) and Cu(II) ions participate in numerous metabolic pathways as components of cuproenzymes, like copper–zinc superoxide dismutase, ceruloplasmin, and group of amine oxidases.^2−4^ Notably, various cancer types demonstrate increased copper accumulation, which is associated with a substantial role of copper in proliferation or angiogenesis, highlighting copper’s significant involvement in proliferation, angiogenesis, tumorigenesis, and cancer development.^5−7^ The distinct responses of tumor and normal cells to copper present new opportunities for developing efficient copper-based anticancer agents.

When considering the antiproliferative potential of copper(II) complexes, special attention is given to Cu(II)–terpyridine systems.^8−15^ The chelating ability of 2,2′:6′,2″-terpyridine (terpy) and its derivatives (R-terpy) enhances complex stability, and their molecular structures facilitate noncovalent interactions with DNA through the major groove,^16−18^ π-stacking between the plane of the aromatic rings and DNA base pairs,^19−27^ and electrostatic binding.^28−30^ Moreover, many Cu(II)–terpyridine systems can generate reactive oxygen species (ROS), giving rise to damage in the cytoplasm, mitochondria, and DNA.^18,20,21,31−33^ Importantly, a broad range of possible structural modifications for 2,2′:6′,2″-terpyridine provide opportunities to enhance the anticancer profile and reduce side effects of Cu-based anticancer agents. Substituents introduced into the terpy framework have been shown to control the electronic and structural features of the resulting Cu(II) complexes, and thus their cytotoxicity behavior.^12,17,20,21,23,24,30,34−37^ Exemplarily, the five-coordinated Cu(II) complex [CuCl_2_(R-terpy)] with 1-methyl-1*H*-pyrrol-2-yl-2,2′:6′,2″-terpyridine exhibiting a rare trigonal-bipyramidal geometry induced by the bulky 1-methyl-1*H*-pyrrole substituent, demonstrated no cytotoxic activity in tumor HCT116, A2780, A549, and MCF7 cell lines.^38^ In contrast, the highly promising cytotoxicity of [Cu(4′-(2-quinolinyl)-terpy)Cl](PF_6_) in HCT116 cancer cell line was manifested by the high internalization of HCT116 cells via energy-dependent and energy-independent mechanisms. This complex showed the ability to intercalate and destabilize DNA, induce intracellular ROS triggering apoptosis and autophagy, and display low toxicity in an in vivo model of CAM. This cytotoxicity was correlated with the unique square-planar geometry of the complex.^39^

**Scheme 1:**
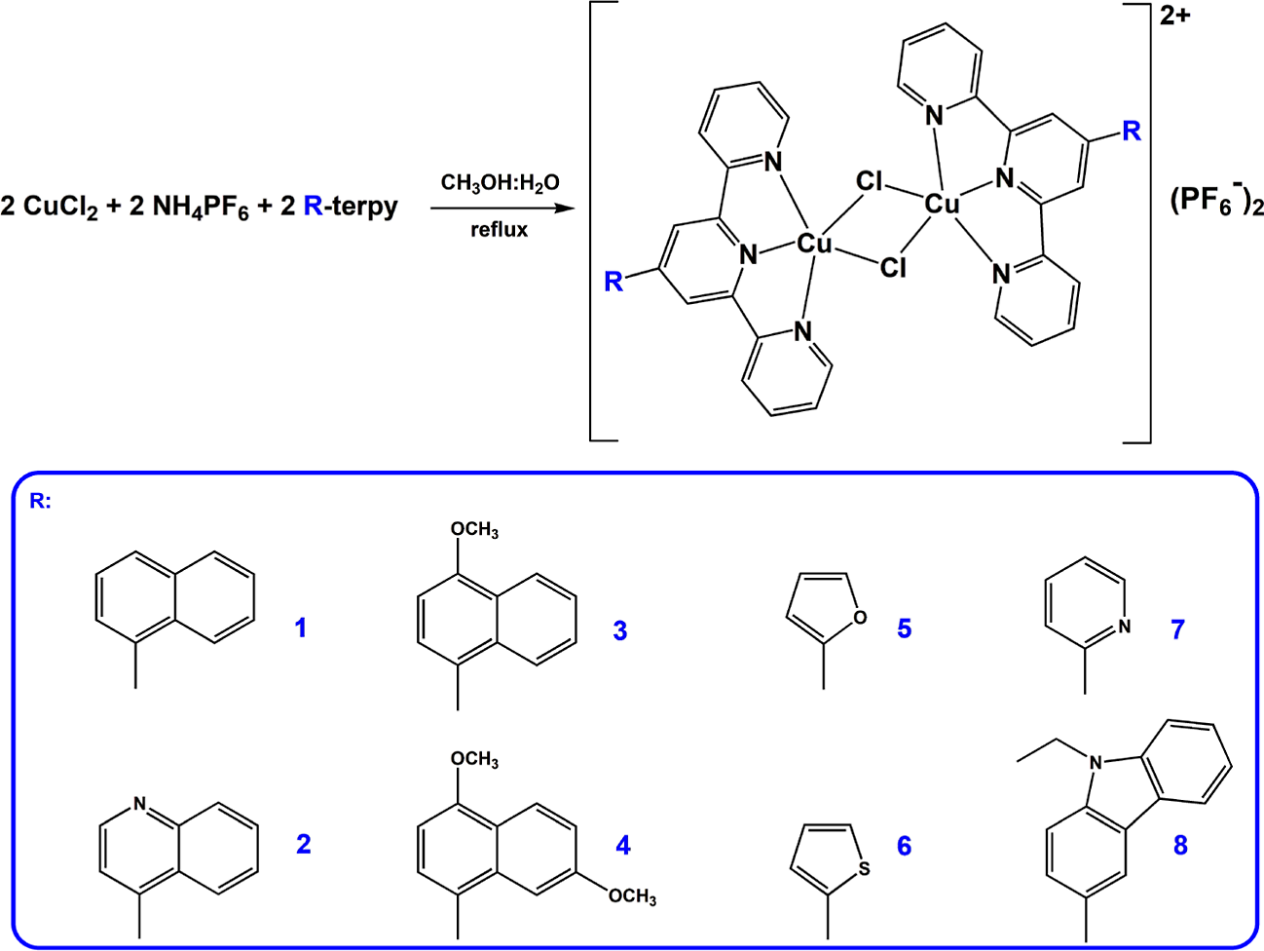
Schematic Route of Synthesis of the [Cu_2_Cl_2_(R-terpy)_2_](PF_6_)_2_ Coordination Compounds Designed in the Study Aromatic substituents R: 1-naphthyl, 4-quinolinyl, 4-methoxy-1-naphthyl, 4,7-dimethoxy-1-naphthyl, 2-furanyl, 2-thiophenyl, 2-pyridynyl, and *N*-ethyl-9*H*-carbazol-3-yl.

In the current paper, we report the comprehensive structural and anticancer characteristics of a series of [Cu_2_Cl_2_(R-terpy)_2_](PF_6_)_2_ (Scheme 1).

In the designed series [Cu_2_Cl_2_(R-terpy)_2_](PF_6_)_2_, the selection of substituents was aimed to determine the impact of the dihedral angle between the substituent plane and planar terpy framework, the introduction of additional methoxy groups, the size of the aromatic ring, and the presence of various heteroatoms in appended substituents on the therapeutic indices of Cu(II) systems.

Interestingly, the Cu(II) complexes exhibited a higher selectivity for colorectal carcinoma cell lines, with the most promising ones demonstrating enhanced selectivity toward the colorectal carcinoma resistant to doxorubicin (Dox). Both in vitro and in vivo studies were conducted to gain insights into the biological activity of the most promising complexes. These studies encompassed the assessment of the mechanism of cell death triggered by the Cu(II) complexes, their subcellular location, their cytostatic, metastatic, and pro- or antiangiogenic properties, as well as their ability to interact with proteins and DNA and induce reactive oxygen species (ROS).

## Results and Discussion

### Synthesis, Molecular Structure, and Spectroscopic Characterization

To synthesize Cu(II) coordination compounds [Cu_2_Cl_2_(R-terpy)_2_](PF_6_)_2_ (**1**–**8**, Scheme 1), the methodology is based on the reaction of a methanolic solution of the corresponding substituted 2,2′:6′,2″-terpyridine (R-terpy) with a water–methanol equimolar mixture of CuCl_2_ and NH_4_PF_6_ were employed.^40^ The resulting green and greenish-blue precipitates were recrystallized through slow evaporation from an acetonitrile–water solution at room temperature, yielding X-ray quality monocrystals for all investigated compounds. The purity of obtained complexes was evidenced by elemental analyses, HRMS, and UPLC techniques.

High-resolution mass spectrometry (HRMS) analysis conducted in positive ion mode (Figure S1) revealed that the analyzed compounds **1–8** are ionic complexes with a doubly cationic moiety and a double hexafluorophosphate anion, characterized by the simplified formula [Cu_2_Cl_2_(R-terpy)_2_]^2+^[PF_6_^–^]_2_. Accordingly, electrospray ionization-MS (ESI-MS) spectra exhibited *m*/*z* signals corresponding to the isotopic distributions of ions constituting half of the total cationic mass, conforming to the formula [CuCl(R-terpy)]^+^.

UPLC analysis, utilizing a PDA detector, was performed within the wavelength range of 210–400 nm. The spectra of the analyzed compounds **1**–**8** consist of several characteristic bands in the UV radiation range. Three main absorption regions were distinguished: the first, most distinctive, with maximum absorption at 210–226 nm; the second and third regions with bands at wavelengths of 286–288 and 323–338 nm, respectively. No other impurities were observed in the examined radiation range, as presented in Figure S2.

**Figure 1:**
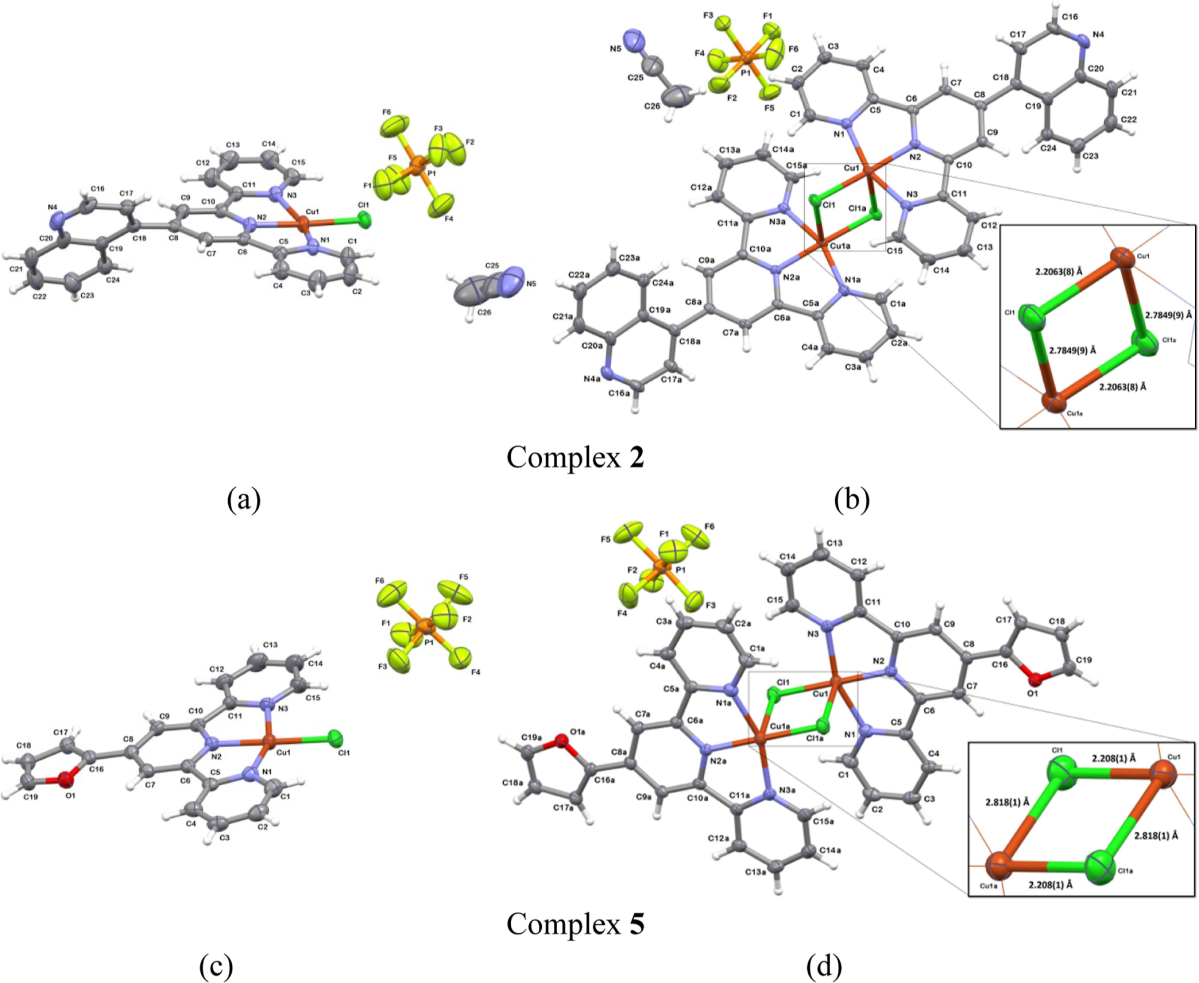
Representative structures of **2** and **5**. Asymmetric units (a,c) and dimeric structures (b,d). Symmetry operation: (1 – *x*,1 – *y*,1 – *z*). Close-ups display the bond lengths of the Cu(μ-Cl)_2_Cu core. For structures **1**, **3**, **4**, **6**, **7**, and **8**; see Figure S3.

The complexes **1**–**8** crystallize in the centrosymmetric monoclinic or triclinic space groups (Table S1), and their asymmetric units comprise the complex ion [CuCl(R-terpy)]^+^, counterion PF_6_^–^, and for structures **1**, **2**, **4**, and **8**—also solvent molecules. Except for compound **2**, however, solvent molecules (CH_3_OH, CH_3_CN, or H_2_O) could not be modeled satisfactorily, and they were removed from the electron density map using the Olex2 solvent mask command.^41^ As revealed by X-ray analysis, the chloride ion of [CuCl(R-terpy)]^+^ acts as a bridging ligand, simultaneously occupying the apical position in the neighboring complex ion [CuCl(R-terpy)]^+^. This implies the existence of the positively charged dimeric units [Cu_2_Cl_2_(R-terpy)_2_]^2+^ in the solid structures, counterbalanced by PF_6_^–^ ions (Figures 1 and S3).

The geometry of each copper(II) center in [Cu_2_Cl_2_(R-terpy)_2_]^2+^ is most accurately described as a distorted square pyramid, with the Addison parameter^42^ varying from 0.24 in **1** to 0.35 in **6** (Table S2). The pyramid base of **1**–**8** is formed by three nitrogen atoms of the R-terpy ligand and one of the bridging chloride ligands, while the axial position is defined by the other chloride bridging ion. The Cu(II) ion is slightly elevated above the basal plane toward the axial chloride ion (from 0.063 Å for **6** to 0.145 Å for **1**). In line with a Jahn–Teller distortion of Cu(II) ions, the Cu–Cl_apical_ bond length [2.7458(12)–2.896(1) Å] is considerably longer than the Cu–Cl_basal_ one [2.2047(10)–2.2243(12) Å] (Table S3). The significant difference in Cu–Cl_apical_ and Cu–Cl_basal_ distances results in a rhomboidal geometry of the Cu(μ-Cl)_2_Cu core in dimeric units [Cu_2_Cl_2_(R-terpy)_2_]^2+^, composed of two square pyramids connected through bridging chloride ligands. The intradimer Cu···Cu distance varies from 3.4920(7) Å in **5** to 3.6864(7) Å in **4**, while Cu–Cl–Cu and Cl–Cu–Cl bond angles of the Cu(μ-Cl)_2_Cu core fall within the ranges 87.13(3)–91.52(4)° and 88.48(4)–92.87(4)°, respectively.

In all Cu(II) complexes, the R-terpy ligand coordinates the Cu(II) ion through three nitrogen atoms of the terpy framework. The Cu–N_central_ bond lengths [1.919(3)–1.941(4) Å] are noticeably shorter compared to those of the peripheral pyridyl rings [2.008(3)–2.039(4) Å], and the bite angles N–Cu–N are much smaller than the ideal value of 90° [79.45(16)–80.54(12)°] due to κ^3^N-coordination of R-terpy and the formation of two fused five-member chelate rings with Cu(II) ions. The terpy framework is approximately planar, with dihedral angles between the mean planes of the central pyridine and terminal aromatic rings ranging from 1.23 to 10.14°. Considering the R-terpy ligand as a whole, noticeable differences among examined structures concern the twisting of the pendant substituent in relation to the central pyridine ring of terpy. The largest value of the dihedral angle between the central pyridine and appended group of 55.54° was found for **2**, while the pendant substituent of **5** maintains near coplanarity (5.17°) with the central pyridine plane (Table S2). Structural data regarding short intra- and intermolecular hydrogen bonds, π···π, and *X*–*Y*···π interactions in structures of **1**–**8** are available in Supporting Information (Tables S4–S6).

The FT-IR spectra of **1**–**8** exhibit bands attributable to vibrations of the coordinated R-terpy ligand and PF_6_^–^ counterion (Figure S4). The characteristic peaks of R-terpy occur in the ranges 3170–2840 cm^–1^ (aromatic C–H stretching vibrations), 1620–1510 cm^–1^ [ν(C=N) and ν(C=C) stretches], 1490–1000 cm^–1^ [ν(C–N) and ν(C–C) vibrations], and 800–600 cm^–1^ (aromatic C–H deformation vibrations), while intense bands at ∼40 and ∼558 cm^–1^ support the presence of PF_6_^–^ ions.^43^

DMSO solutions of the complexes **1**–**8** exhibit molar conductivities in the range 53.6–71.2 S·cm^2^·mol^–1^, and negligible variations in their molar conductivities are observed over a 5 h period (Figure S5 and Table S7). These values, typical for 1:1 electrolytes,^44^ indicate that the dinuclear arrangement observed in the solid-state structures of complexes **1**–**8**, corresponding to the 2:1 electrolyte nature in solution, is not preserved in DMSO.

To further explore the molecular structures of **1**–**8** in solution, UV–vis spectra of all Cu(II) complexes were recorded in DMSO and compared to the corresponding diffuse reflectance spectra (Table S8 and Figures S6,S7). The high similarity in the absorption profiles recorded in solution and the solid state allows us to assume a distorted tetragonal pyramidal environment around the Cu(II) ions in solution, likewise to the solid state. This implies that dissociation of [Cu_2_Cl_2_(R-terpy)_2_]^2+^ is followed by the instant coordination of the solvent molecule, forming [CuCl(solvent)(R-terpy)]^+^ counterbalanced by PF_6_^–^ in solution.

For square-pyramidal Cu(II) complexes,^45^ three spin-allowed d_*xy*_ → d_*x*^2^–*y*^2^_, d_*yz*_,d_*xz*_ → d_*x*^2^–*y*^2^_, and d_*z*^2^_ → d_*x*^2^–*y*^2^_ transitions are expected. However, due to their energy proximity, they generally remain unresolved, resulting in a weak and broad band observed in the UV–vis spectra of such systems. In the title Cu(II) complexes, the broad band corresponding to d–d transitions appears in the range of 550–900 nm in DMSO and 500–1000 nm in diffuse reflectance spectra. The absorption in the range 375–475 nm, observed for the DMSO solution of the complexes **3**, **4**, and **8** with π-conjugated and electron-donating substituents, is most likely assigned to an intraligand charge transfer transition (ILCT) originating from charge delocalization from the substituent to the terpy acceptor moiety.

The chelation of R-terpy to the Cu(II) ion enhances the electron-withdrawing character of the terpy moiety, promoting intramolecular charge transfer. The higher energy absorptions in the UV–vis spectra of **1**–**8** are contributed by ligand-to-metal charge-transfer (LMCT) and π → π* (IL) transitions (Figures S6 and S8).

Most importantly, all Cu(II) complexes remain stable in DMSO and 10 mM phosphate-buffered saline (PBS, pH 7.4) solutions. As demonstrated in Figures S9 and S10, there are no noticeable changes in the absorbance profiles of these systems in UV–vis spectra recorded at regular time intervals for 48 h. The stability of complexes **1**–**8** in the solution and physiological environment justifies their further use in biological studies.

### Cell Viability Assays in 2D Cell Cultures

The in vitro cytotoxic potential of Cu(II) complexes in tumor and healthy cells was assessed using the MTS assay, a colorimetric method that allows the quantification of viable cells in culture.^46^

**Figure 2:**
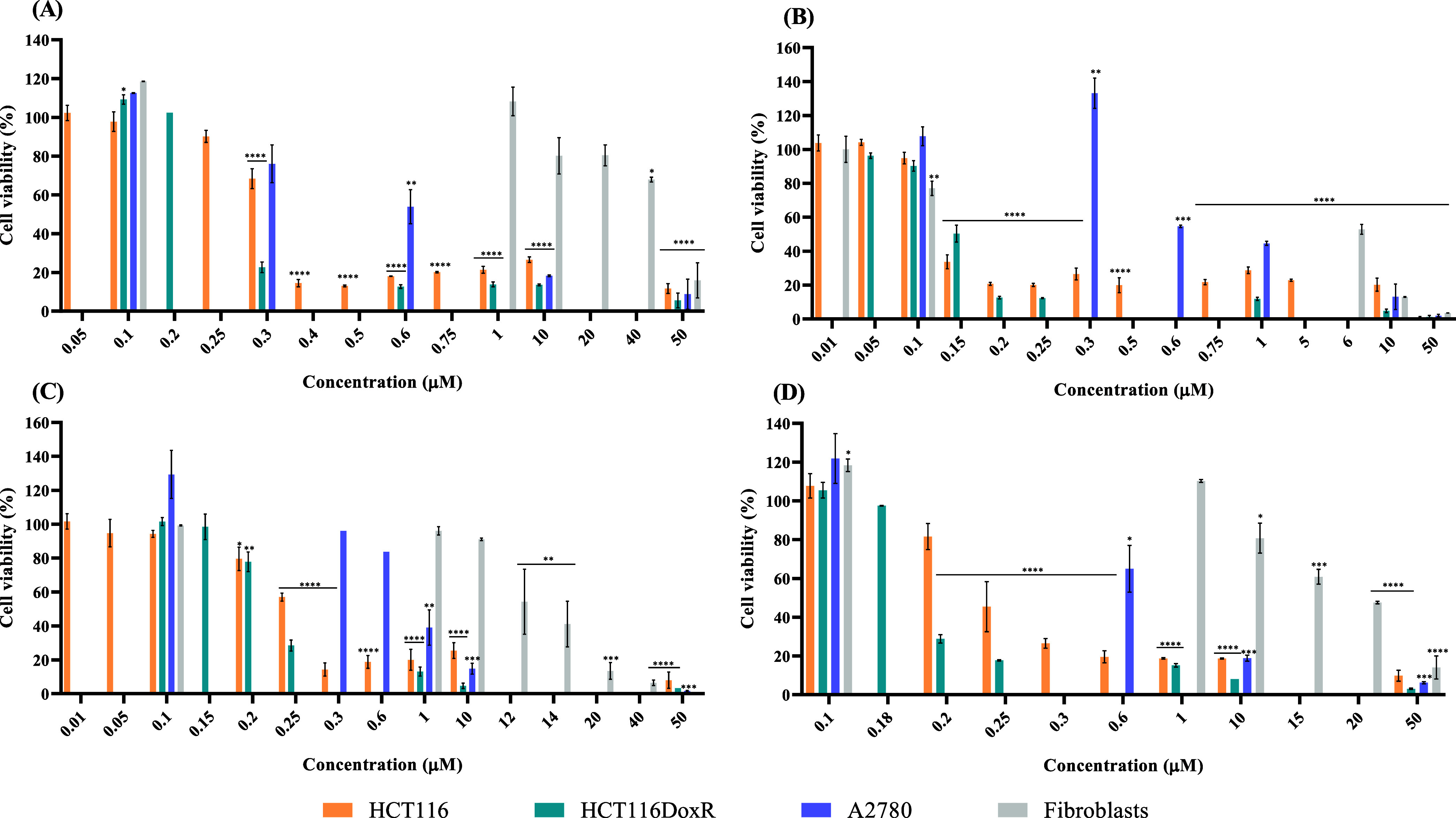
Cell viability of HCT116, HCT116DoxR, and A2780 tumor cell lines and primary normal fibroblasts after exposure to different concentrations of copper complexes **2** (A), **3** (B), **5** (C), and **7** (D) for 48 h. DMSO in the same % as in the complexes was used as the vehicle control. Data are expressed as the mean ± SEM of at least two biological independent assays. Statistical significance was assessed relative to control (DMSO) by the one-way ANOVA method (*p < 0.05; **p < 0.01; ***p < 0.001; ****p < 0.0001).

Cell viability was determined after incubation of complexes for 48 h in three tumor cell lines, namely, HCT116 (colorectal carcinoma cell line-sensitive), HCT116DoxR [Dox-resistant colorectal carcinoma cell line], A2780 (ovarian carcinoma cell line), and in a healthy cell line (normal human primary dermal fibroblasts). A loss of cell viability with the increase of complex concentrations is observed in Figures 2 and S11. The relative IC_50_ values of each complex (the complex concentration that induces a 50% loss of cell viability) were calculated in the respective cell line (Table 1). The IC_50_ values for the Cu(II) complexes are in the 0.1–0.3 μM range for the HCT116 and HCT116DoxR cell lines, while in the A2780 cell line are in the range of 0.3–1.1 μM, demonstrating the greatest cytotoxicity for the sensitive and resistant colorectal carcinoma lines (Table 1 and Figures 2 and S11). Interestingly, IC_50_ values for almost all Cu(II) complexes are higher in HCT116 line, compared to the Dox-resistant cell line (HCT116DoxR), indicating a greater antiproliferative activity in the latter (Table 1). Furthermore, the IC_50_ values for these complexes in the Dox-sensitive colorectal carcinoma cell line are lower than the IC_50_ values for the antitumor agent Dox (0.50 μM) or cisplatin (15.60 μM) (Table 1 and Figure S12). It is interesting to note that the IC_50_ values for these Cu(II) complexes for the HCT116DoxR cell line are significantly lower than the IC_50_ of Dox (>6 μM^47^), demonstrating interest in their application in this resistant cell line (Table 1 and Figure S12).

**Table 1:** Relative IC_50_ Values Obtained for Each Copper(II) Complexes in HCT116, HCT116DoxR, A2780, and Fibroblast Cell Lines and Respective SI after 48 h of Exposure^a^

complex	cell line	IC_50_ (μM)	SI
**1**	HCT116	0.16 ± 0.01	19.4
	HCT116DoxR	0.12 ± 0.01	24.5
	A2780	0.80 ± 0.05	3.8
	fibroblasts	3.04 ± 0.02	
**2**	HCT116	0.31 ± 0.01	101.6
	HCT116DoxR	0.25 ± 0.02	**124.7**
	A2780	0.54 ± 0.07	58.0
	fibroblasts	31.36 ± 0.02	
**3**	HCT116	0.13 ± 0.02	49.8
	HCT116DoxR	0.15 ± 0.01	**42.2**
	A2780	0.66 ± 0.07	9.5
	fibroblasts	6.24 ± 0.03	
**4**	HCT116	0.15 ± 0.04	17.1
	HCT116DoxR	0.16 ± 0.01	16.5
	A2780	0.49 ± 0.10	5.4
	fibroblasts	2.64 ± 0.10	
**5**	HCT116	0.26 ± 0.03	47.6
	HCT116DoxR	0.22 ± 0.01	**56.4**
	A2780	0.79 ± 0.05	15.9
	fibroblasts	12.57 ± 0.02	
**6**	HCT116	0.15 ± 0.08	55.2
	HCT116DoxR	0.34 ± 0.02	24.6
	A2780	1.00 ± 0.07	8.5
	fibroblasts	8.45 ± 0.03	
**7**	HCT116	0.23 ± 0.01	71.3
	HCT116DoxR	0.19 ± 0.01	**82.8**
	A2780	1.13 ± 0.20	14.2
	fibroblasts	16.08 ± 0.05	
**8**	HCT116	0.10 ± 0.03	75.1
	HCT116DoxR	0.19 ± 0.06	38.4
	A2780	0.37 ± 0.06	20.0
	fibroblasts	7.36 ± 0.04	
Dox	HCT116	0.50 ± 0.10	24.2
	HCT116DoxR	>6^47^	3.4
	A2780	0.10 ± 0.04	121
	fibroblasts	12.10 ± 0.20	
cisplatin	HCT116	15.60 ± 5.30	0.6
	HCT116DoxR		
	A2780	1.90 ± 0.20	4.6
	fibroblasts	8.80 ± 2.90	

a IC_50_ values are expressed as the mean ± SEM of at least two biological independent assays.

Since one of the main objectives of researching new complexes is to reduce their side effects on normal tissues, it is essential that these Cu(II) complexes present significantly higher IC_50_ values in healthy human cell lines compared to tumor cell lines. Therefore, their relative IC_50_ values were also determined in fibroblasts due to their importance in the tumor microenvironment^48^ (Table 1).

From the values obtained in fibroblasts, the selectivity index (SI), the ratio between the IC_50_ determined in fibroblasts, and the IC_50_ determined in the respective human tumor cell line, was determined, being a measure of the specificity of each complex for each tumor line (Table 1).

Analyzing the SI values, they are higher for the colorectal carcinoma lines compared to the ovarian carcinoma line (Table 1). When we compare the two colorectal carcinoma lines, we may observe that the SI values vary in the order **2** > **8** > **7** > **6** > **3** > **5** > **1** > **4** in the case of the HCT116 line and in the order **2** > **7** > **5** > **3** > **8** > **6** > **1** > **4** for HCT116DoxR (Table 1), but overall they are higher for complexes **2**, **5**, and **7** in the HCT116DoxR line (highlighted in bold in Table 1). Although complex **3** is more specific for the HCT116 line, the SI value obtained is only slightly higher than that obtained for the HCT116DoxR line (not significant). Thus, of the various complexes studied, it was concluded that complexes **2**, **3**, **5**, and **7** have a high therapeutic potential for the HCT116DoxR line, which is why the remaining biological assays were performed in this cell line.

It is important to analyze if the antiproliferative activities observed for the eight Cu(II) complexes are due to the respective ligands. In this regard, cell viability was performed for each of the ligands on HCT116DoxR and fibroblasts. The IC_50_ values obtained for HCT116DoxR and fibroblasts are described in Table 2, while the corresponding cell viability data are presented in Figures S13 and S14, respectively. The IC_50_ of the ligand **L3** in fibroblasts was previously obtained.^49^

**Table 2:** Relative IC_50_ and SI Values Obtained for Each Ligand in HCT116DoxR Cell Line and Fibroblasts after 48 h of Exposure^a^

	IC_50_ (μM)		
ligand	HCT116DoxR	fibroblasts	SI (HCT116DoxR)
**L1**	≈0.20	>50	285.1
**L2**	0.40 ± 0.02	>50	119.9
**L3**	0.20 ± 0.02	0.95 ± 0.08^49^	4.9
**L4**	0.40 ± 0.25	13.05 ± 3.18	36.1
**L5**	0.60 ± 0.02	>50	80.0
**L6**	0.70 ± 0.02	≈50	74.9
**L7**	0.50 ± 0.06	≈50	110.1
**L8**	0.40 ± 0.04	≈10	23.4

a IC_50_ values are expressed as the mean ± SEM of at least two biological independent assays.

Comparing the IC_50_ and SI values in HCT116DoxR obtained for each complex and the corresponding ligand (Tables 1 and 2), we can verify the following. (1) Excluding ligand **L1**, the ligands **L2**, **L7**, and **L5** are the ligands with the greatest specificity for the HCT116DoxR line (in this order), as was observed for their respective complexes **2**, **7**, and **5**, indicating that these ligands contribute directly to the antiproliferative potential observed for these complexes. (2) The coordination of ligands **L2**, **L3**, and **L8** with the two Cu(II) metal centers and the two chlorine atoms increases their selectivity for the HCT116DoxR line. (3) The ligands **L1**, **L4**, **L5**, **L6**, and **L7** are less cytotoxic to fibroblasts than their respective complexes, increasing their SI relative to the Cu(II) complexes.

Thus, based on the conclusions drawn above, we may see that regarding the SI for HCT116DoxR, the samples analyzed can be ordered as **L1** > **2** > **L2** > **L7** > **7** > **L5** > **L6** > **5** > **3** > **8** > **L4** > **6** > **1** > **L8** > **4** > **L3**. Although some ligands are more selective than the respective complexes **2**, **3**, **5**, and **7**, the IC_50_ values determined in the HCT116DoxR line after 48 h of exposure are higher than those found for the complexes. Although SI is an important parameter for determining the efficacy of a therapeutic agent, it is not the only characteristic that should be considered. A lower IC_50_ value indicates that the complex can exert the same effect (50% reduction in cell viability) at a lower concentration, reducing the likelihood of the development of systemic toxicity.^50^ Furthermore, since these complexes contain copper and copper is an essential micronutrient for the human body, the presence of copper transporters may facilitate the internalization of the complexes.^51^ Despite the ligands having cytotoxic activity in cells, we will not further explore their mechanism of action as it is much more complicated to track organic molecules within a cellular context, and as Cu is an essential metal (in low concentrations), cells already have mechanisms for their uptake, making them more suitable for further biological studies. This is an important reason for performing additional biological studies to better characterize the cellular effects of the most effective complexes.

### Cell Viability Assays in 3D HCT116DoxR Spheroids

**Figure 3:**
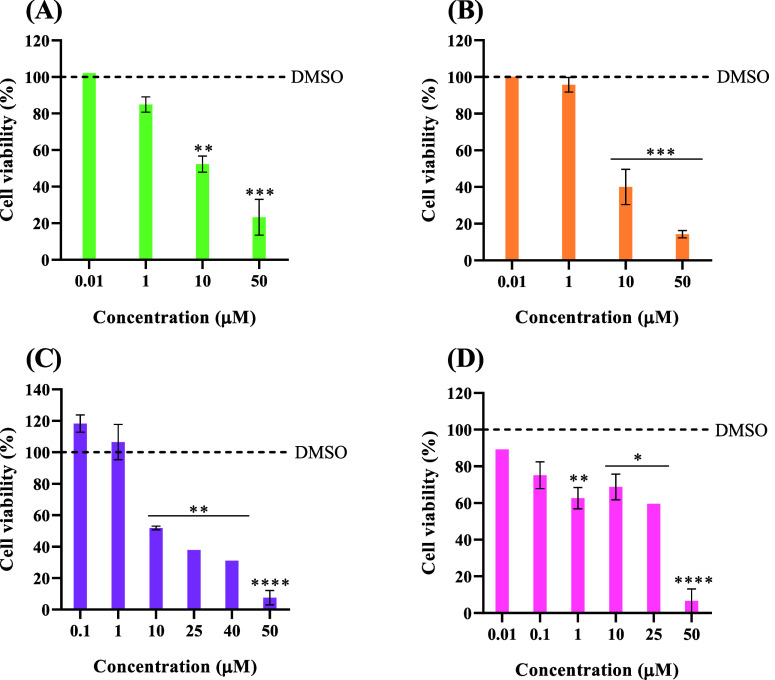
Cell viability of 6-day-old HCT116DoxR spheroids after 48 h of exposure to different concentrations of complexes **2** (A), **3** (B), **5** (C), and **7** (D) (8 days in total). DMSO in the same % as in the complexes was used as the vehicle control. Data are expressed as the mean ± SEM of at least two biological independent assays. Statistical significance was assessed relative to control (DMSO) by the one-way ANOVA method (*p < 0.05; **p < 0.01; ***p < 0.001; ****p < 0.0001).

To compare the cytotoxic potential of the copper complexes **2**, **3**, **5**, and **7** in 2D and 3D cultures, the MTS assay was also performed on HCT116DoxR spheroids with 6 days of growth and submitted to 48 h of exposure to the complexes (8 days in total) (Figure 3). Table 3 shows the comparison of the IC_50_ values obtained for these complexes in 2D and 3D cultures of HCT116DoxR cells.

**Table 3:** Comparison of IC_50_ Values Obtained for Complexes **2**, **3**, **5**, and **7** in 2D and 3D Cultures of HCT116DoxR Cells 48 h of Exposure^a^

	IC_50_ (μM)	
complex	HCT116DoxR 2D	HCT116DoxR 3D
**2**	0.25 ± 0.02	15.25 ± 0.80
**3**	0.15 ± 0.01	6.35 ± 0.12
**5**	0.22 ± 0.01	13.97 ± 0.60
**7**	0.19 ± 0.01	30.48 ± 0.19

a Relative IC_50_ values are expressed as the mean ± SEM of at least two biological independent assays.

As expected, it is possible to denote an increase in the IC_50_ values obtained in 3D spheroids compared to the IC_50_ values obtained in 2D cultures (Table 3). Indeed, the IC_50_ values in 3D models are approximately 61×, 43×, 63×, and 157× higher for complexes **2**, **3**, **5**, and **7**, respectively, compared to the values obtained for 2D cultures. The greater complexity of the cellular microenvironment in 3D structures results in the complexes facing several constraints before reaching each individual cell since they need to diffuse within the spheroid before cell penetration. Since the diffusion of the complex in the spheroid accompanies the increase in the size of this structure, spheroids with 6 days of growth were used in these studies so that their size was controlled.^52,53^ Interestingly, in spheroids, the order of cytotoxicity is **3** > **5** > **2** > **7**, while in 2D cultures it is **3** > **7** > **5** > **2** (Table 3), with complex **3** always being the most cytotoxic in this resistant cell line. Moreover, it is important to note that for complex **3**, the IC_50_ in 3D is still in the low micromolar range (Table 3). The results obtained (Table 3) are in agreement with other reports,^48,54^ with a higher concentration of complex being needed in the 3D structure to achieve the same biological effect seen in 2D. The antiproliferative potential values obtained in spheroids are more like those that would be verified in an in vivo model.

However, for simplicity, the remaining biological assays for determining the mechanisms of action of complexes **2**, **3**, **5**, and **7** were performed in 2D cultures.

### Cellular Internalization

After proving that the Cu(II) complexes induce a loss of cell viability and before determining their mode of action, it is important to understand their temporal internalization within cells and their subcellular accumulation.

**Figure 4:**
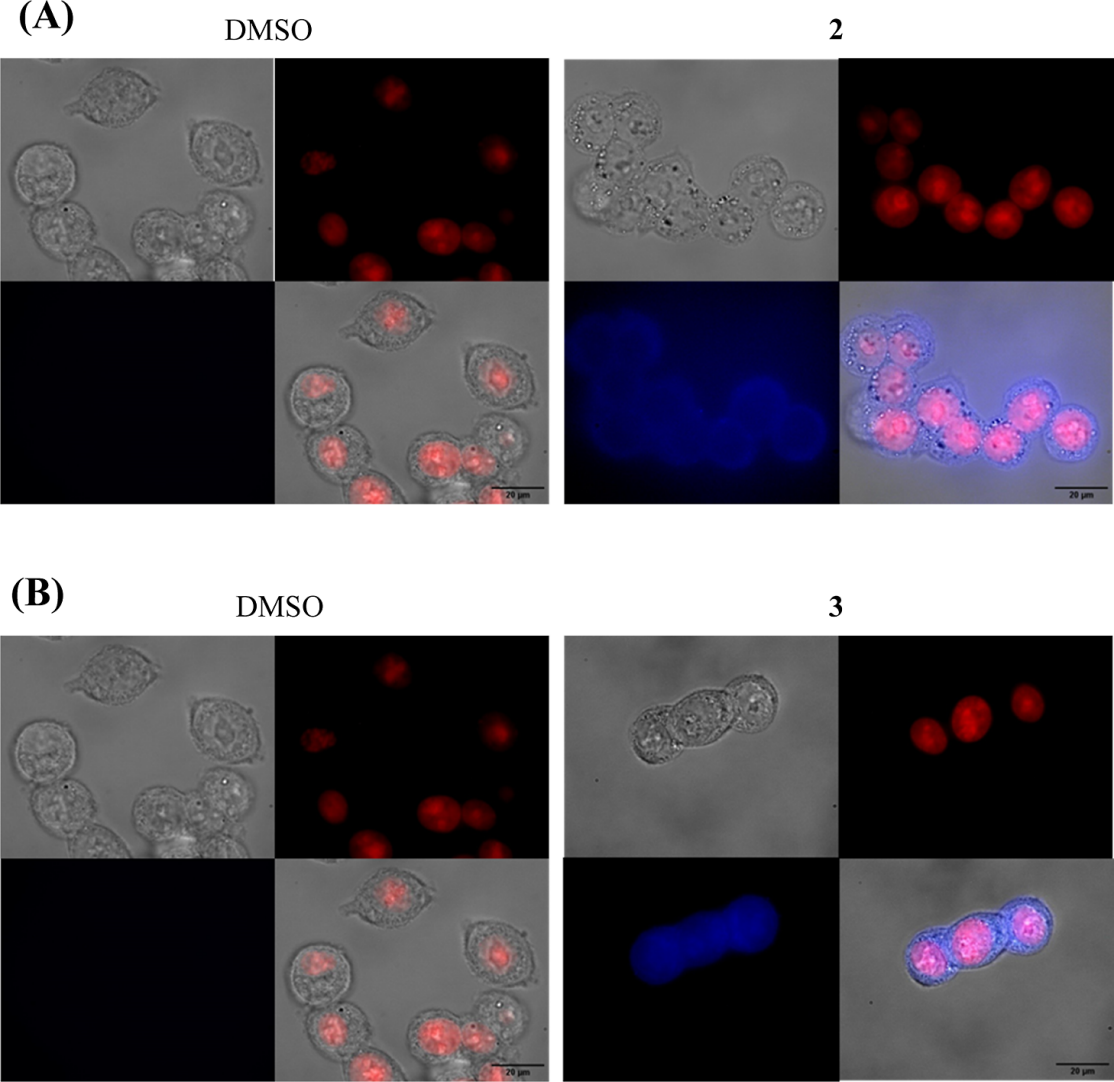
Fluorescence microscopy of HCT116DoxR cells incubated for 3 h with 10× the IC_50_ of the complexes **2** (A) and **3** (B) (right) or with 0.1% (v/v) DMSO (negative control, left). Nuclei have been labeled with PI (red), while the fluorescent complexes are in blue. Complexes present a maximum excitation at 290 nm in the UV region and their maximum emission at 410–420 nm in the blue region.

**Figure 5:**
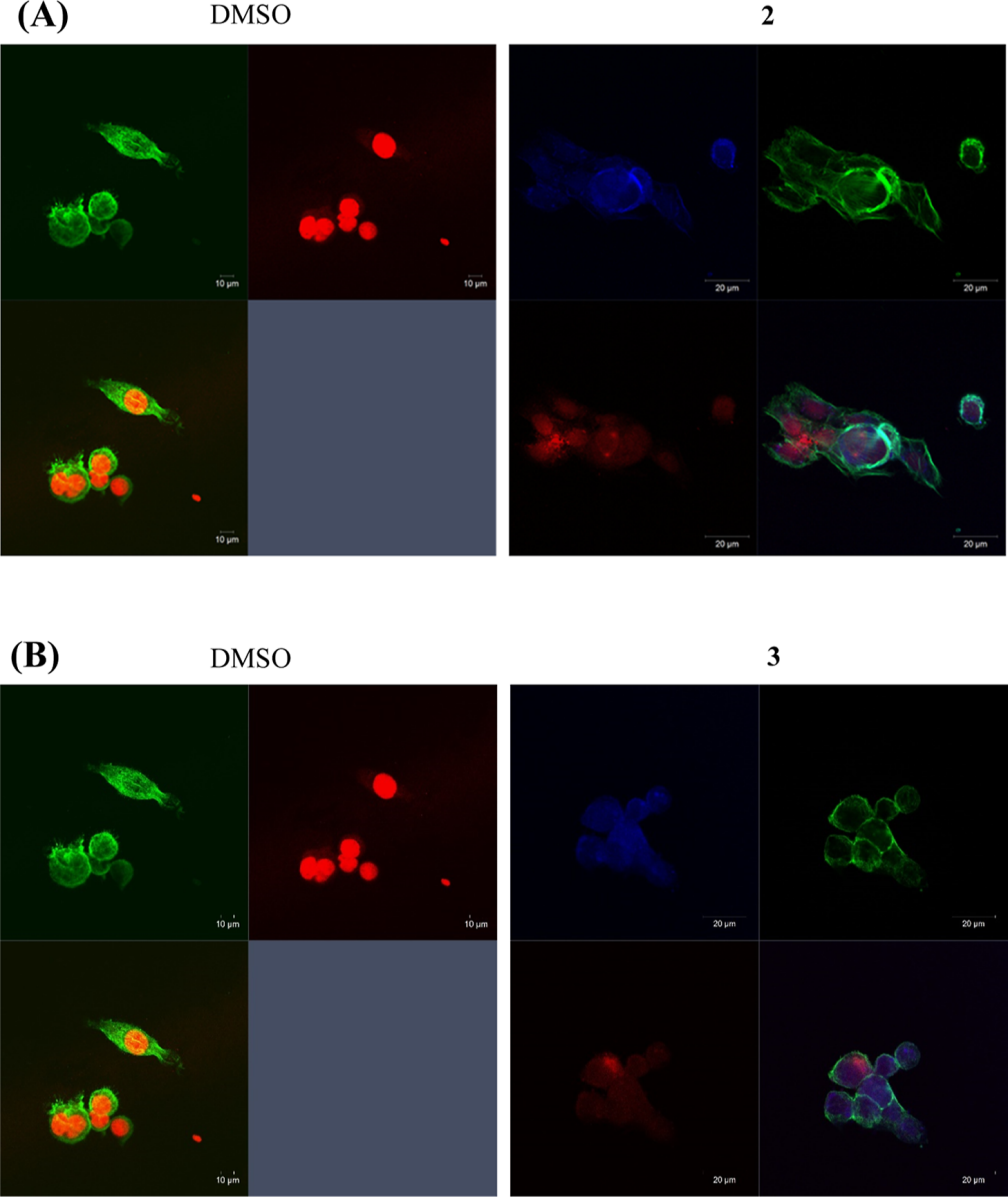
Confocal microscopy of HCT116DoxR cells incubated for 6 h with 10× the IC_50_ of the complexes **2** (A) and **3** (B) (right) or with 0.1% (v/v) DMSO (negative control, left). Nuclei were labeled with PI (red), the actin cytoskeleton was labeled with Alexa Fluor 488 phalloidin (green), while the fluorescent complexes are in blue. Complexes present a maximum excitation at 290 nm in the UV region and their maximum emission at 410–420 nm in the blue region.

Due to their fluorescent properties, complexes **2** and **3** internalization was studied by fluorescence and confocal microscopy in HCT116DoxR cells exposed for 3 and 6 h to each Cu(II) complex. As a negative control, images were also taken of HCT116DoxR cells exposed to 0.1% (v/v) DMSO. In this assay, a concentration of 10×, the IC_50_ of the complexes was used so that their intracellular fluorescence levels could be clearly analyzed. After incubation with the complexes or DMSO, the cells were labeled with PI to stain the nuclei and, in the case of confocal microscopy images, with Alexa Fluor 488 phalloidin to stain the actin cytoskeleton.^47,55^ The images obtained are shown in Figures 4, 5, and S15–S17.

Analyzing the images obtained (Figures 4 and 5), there is no blue fluorescence detected in HCT116DoxR cells incubated in the absence of the complexes (DMSO control), which confirms that all the blue fluorescence detected is due to their presence. Considering the fluorescence emitted by complex **2**, after 3 h of incubation with the HCT116DoxR cells, it is possible to verify that this complex can internalize into the cells (Figures 4A, S15,S17A), being also detected complex’ aggregates in their vicinity (Figure S17A), indicating that the internalization of the complex is not total after 3 h of incubation. In contrast, the image obtained after 6 h of incubation (Figure 5A) shows that the internalization of this complex is almost complete. In terms of intracellular localization, the images seem to indicate that this complex accumulates mainly in the cell membrane and cytosol, areas where the blue fluorescence is most intense, but is also present in other regions of the cell.

Regarding complex **3**, the fluorescence detected in the vicinity of the cells after 3 h of incubation is lower than that detected in the case of complex **2**, which seems to indicate that the amount of complex internalized is higher after this time (Figures 4 and S15–17). In addition, the fluorescence of this complex is detected throughout the cell, including in the nucleus (Figures 4B, 5B, S16,S17B). To better clarify these subcellular localizations regarding complexes **2** and **3** and to gather information regarding the internalization and subcellular localization of complexes **5** and **7** in HCT116DoxR cells, ICP-AES was used to quantify the metal (in this case, copper) present in each sample.^48^ As copper is an essential metal, the results of the cells exposed to the different complexes were normalized to the values of the cells exposed to the DMSO control.

**Figure 6:**
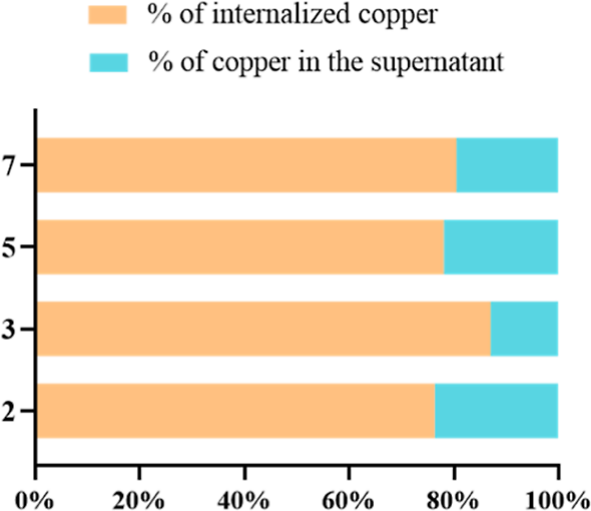
Percentage (%) of copper in the cellular fractions and in the supernatants after 6 h of exposure of HCT116DoxR cells to 20× the IC_50_ of the complexes **2**, **3**, **5**, and **7**.

In the first approach, the cellular fractions and the respective supernatants were separated after incubation of cells for 6 h with the Cu(II) complexes (**2**, **3**, **5**, and **7**), and the results are presented in Figure 6. Due to the limit of detection of the ICP-AES technique, in this assay we used a 20× IC_50_ of each copper complex. It is verified that after 6 h of exposure of HCT116DoxR cells to 20× the IC_50_ of the complexes, ∼90% of **3** is internalized, while the % of internalization for the other complexes is ∼80%. Thus, complex **3** has the highest % of internalization, followed by **7**, **5**, and **2** (in this order). These results can be correlated with the results obtained at 3 h by fluorescence microscopy (Figure 4), where complex **2** aggregates are detected outside the cell and are not observed in the complex **3** images, and with the cell viability results, where greater cytotoxicity (lower IC_50_ value) was seen for complex **3**, followed by the other complexes in the same order (Table 1).

To study in more detail the subcellular location of the complexes, the Abcam Standard cell fractionation kit (ab109719) was used to obtain the cytosolic, mitochondrial, and nuclear fractions after 6 h of exposure of the HCT116DoxR cells to 20× the IC_50_ of the complexes **2**, **3**, **5**, and **7**. However, according to the supplier, the fractions obtained are not completely pure, and there is contamination with other organelles: the mitochondrial fraction also includes the membrane and the endoplasmic reticulum, while the nuclear fraction includes the cytoskeleton and the Golgi complex.

Since, from the fluorescence images obtained, complex **2** appeared to be mostly in the membrane and it was not possible to clearly identify the organelles where it can internalize, the Cell Signaling cell fractionation kit (#9038) was also used so that the cell fractions obtained were the cytosolic fraction, the membrane and organelle fraction, and the nucleus and cytoskeleton fraction to complement the results obtained and better characterize the subcellular localization for this complex.

Table 4 shows the results obtained for complexes **3**, **5**, and **7**. These complexes are mostly detected in the nucleus, cytoskeleton, and Golgi complex, being also capable of internalizing in the mitochondria, membrane, and endoplasmic reticulum. The results for complex **3** agree with the previous fluorescence images (Figures 4 and 5).

**Table 4:** Percentage (%) of Copper after 6 h of Exposure of HCT116DoxR Cells to 20× the IC_50_ of the Complexes **3**, **5**, and **7** and Respective Subcellular Fractionation^a^

	copper %			
	supernatant	nucleus, cytoskeleton, and Golgi complex	mitochondria, membrane, and endoplasmic reticulum	cytosol
**3**	12.98	43.66	33.58	9.78
**5**	21.96	54.99	20.39	2.66
**7**	19.55	45.19	35.26	0.00

a For this analysis, the Abcam Standard cell fractionation kit (ab109719) was used.

The results obtained by integrating the information acquired by the 2 kits after the exposure of the cells to complex **2** are shown in Table 5. The % of complex **2** in the nucleus and cytoskeleton is similar to the % in the mitochondria, membrane, and endoplasmic reticulum, results that agree with the fluorescence and confocal microscopy images, where it was detected that this complex accumulates in the membrane, being also present in other regions of the cell (Figures 4A, 5A, S15,S17A). It should be noted that the % of complex **2** in the Golgi complex is also significant (∼10%).

**Table 5:** Percentage (%) of Copper after 6 h of Exposure of HCT116DoxR Cells to 20× the IC_50_ of Complex **2** and Respective Subcellular Fractionation^a^

	copper %					
	supernatant	nucleus and cytoskeleton	Golgi complex	mitochondria, membrane, and endoplasmic reticulum	other organelles	cytosol
**2**	23.68	33.13	9.59	31.00	0.00	2.60

a The Abcam Standard cell fractionation kit (ab109719) and the Cell Signaling cell fractionation kit (#9038) were used for this analysis.

### Stability of Copper Complexes in Biological Medium

Before evaluating the mechanisms of action of the selected complexes (**2**, **3**, **5**, and **7**), it is crucial to ensure that they are stable and soluble in a biological medium.

For all biological assays, complexes were first dissolved in 100% (v/v) DMSO solution, a polar organic molecule with an amphipathic nature and a high capacity to dissolve a wide range of both polar and apolar molecules. Since it has been shown that concentrations of DMSO higher than 1% (v/v) can induce toxicity in cells, in all biological assays, DMSO concentration was kept at 0.1% (v/v) to ensure no cellular toxicity.^56^

The stability of complexes **2**, **3**, **5**, and **7** was assessed by UV–vis spectroscopy at 0, 24, and 48 h of incubation at 37 °C. The complexes were solubilized in 100% (v/v) DMSO and then diluted in colorless RPMI medium (without phenol red and FBS) to a final concentration of 50 μM.

In Figure S18, it is possible to observe the spectra of all Cu(II) complexes, where high-energy absorption bands correspond to π → π* and *n* → π* transitions with peaks in the 230–330 and 330–400 nm ranges, respectively, that are associated with the aromatic rings of terpyridines.^39,49^ Analyzing the spectra of complexes **2** and **3**, it is possible to detect the existence of 3 bands (maximum ∼240, ∼ 290, and between 320 and 340 nm). The spectrum of complex **5** shows, in addition to the 3 bands mentioned above, a smaller band at 270 nm. Complex **7** only has 2 bands of maximum absorbance at 290 nm and between 320 and 340 nm. Over the 48 h period, there were no changes in these bands, indicating that the four copper complexes are stable in the biological medium (Figure S18).

Despite no structural changes being observed, it is possible to see, for complexes **2**, **3**, and **5**, a slight decrease in the maximum absorbances after 24 or 48 h of incubation compared to the absorbance measured in the freshly prepared solution (0 h), indicating a reduction of their solubility, this effect being more pronounced for **3** (Figure S18). However, we should keep in mind that the internalization of these Cu(II) complexes in cells occurs in the first 6 h of incubation (Figure 6), meaning that this decrease in solubility might not pose a significant concern. Nevertheless, all Cu(II) complex solutions were freshly prepared and added immediately to cells.

### Evaluation of Apoptosis Induction in HCT116DoxR Cell Line by Flow Cytometry

To evaluate the cell death mechanism involved in the cytotoxic effect of the selected complexes, a double-staining with Annexin V-FITC and PI was performed. Annexin V is a protein with a high affinity for phosphatidylserine (PS), which is present on the inner surface of the cell membrane in viable cells.^57^ In an early phase of apoptosis, PS is translocated to the outer layer of the cell membrane, being available for interaction with Annexin V.^57,58^ Additionally, Annexin V is also able to bind to the inner layer of the cell membrane at a later stage of apoptosis, when membrane integrity is lost, making it possible to identify cells undergoing late apoptosis.^58^ The use of Annexin V conjugated with the fluorophore FITC allows the quantification of this interaction and subsequent identification of cells in apoptosis by flow cytometry. Furthermore, PI is a fluorophore that can intercalate with DNA and that can only enter the cell when the membrane is either compromised or ruptured, making it possible to identify cells undergoing late apoptosis and necrosis. Therefore, the double staining makes it possible to distinguish between viable cells (FITC^–^; IP^–^), those undergoing initial apoptosis (FITC^+^; IP^–^), those undergoing late apoptosis (FITC^+^; IP^+^), and those undergoing necrosis (FITC^–^; IP^+^).^55,58^

**Figure 7:**
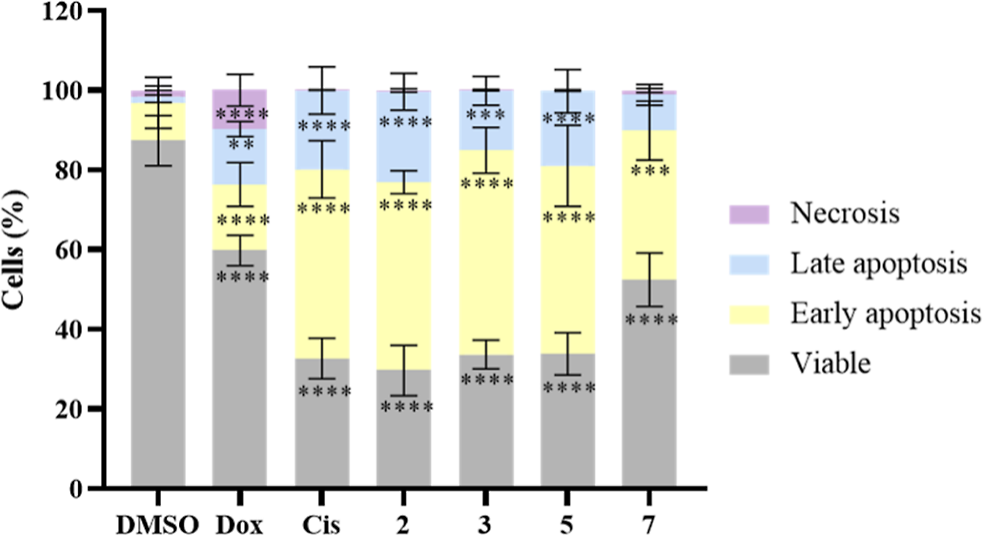
Percentage (%) of viable HCT116DoxR cells (gray), in early apoptosis (yellow), late apoptosis (blue), and necrosis (purple) after 48 h of exposure to the IC_50_ concentrations of complexes **2**, **3**, **5**, and **7**. 0.1% (v/v) DMSO was used as a negative control, while Dox (6 μM) and cisplatin (Cis, 5 μM) were used as positive controls. Data are represented as the mean ± SEM of at least two independent biological assays. Statistical significance was determined relative to the DMSO control by the one-way ANOVA method (*p < 0.05; **p < 0.01; ***p < 0.001; ****p < 0.0001).

Figure 7 and Table 6 show the results obtained in HCT116DoxR cells after 48 h of exposure to the IC_50_ concentrations of complexes **2**, **3**, **5**, and **7**. 0.1% (v/v) DMSO was used as the vehicle control, and Dox (6 μM) and Cis (5 μM) were used as positive controls. The results show that cells exposed to complexes **2**, **3**, and **5** and cisplatin show a high percentage of apoptosis (∼65 to 70%), while cells exposed to complex **7** present ∼46% apoptosis. All the complexes show a low percentage of necrosis (<1.1%). In turn, as expected, the cells exposed to the negative control (DMSO) were mostly viable, showing ∼11% of cell death by apoptosis (Figure 7). Regarding Dox, ∼60% of the cells are viable, while ∼31% are in apoptosis and ∼10% in necrosis. Following normalization of the results obtained for each sample with those obtained for DMSO, it was found that cisplatin and complexes **2**, **3**, and **5** show a ∼6.4-fold increase in apoptotic cells, while complex **7** shows a 5-fold increase of cell death by apoptosis. Thus, these Cu(II) complexes are capable of inducing cell death by apoptosis, with apoptosis values higher than those seen for the positive control Dox and similar to the values observed for the positive control cisplatin.

**Table 6:** Percentage (%) of Viable HCT116DoxR Cells, in Early Apoptosis, in Late Apoptosis, and in Necrosis after 48 h of Exposure to the IC_50_ Concentrations of Complexes **2**, **3**, **5**, and **7**, to DMSO, Dox, and Cisplatin Controls

	cells (%)			
sample	viable	early apoptosis	late apoptosis	necrosis
DMSO	87.4 ± 6.3	9.5 ± 6.4	1.6 ± 1.5	1.5 ± 1.1
Dox	59.8 ± 3.8	16.6 ± 5.5	13.9 ± 1.9	9.8 ± 4.0
cisplatin	32.7 ± 5.1	47.5 ± 7.2	19.8 ± 5.9	0.1 ± 0.1
**2**	29.7 ± 6.3	47.3 ± 2.9	22.7 ± 4.6	0.3 ± 0.4
**3**	33.7 ± 3.6	51.3 ± 5.7	14.9 ± 3.6	0.2 ± 0.2
**5**	33.9 ± 5.3	47.2 ± 10.2	18.7 ± 5.4	0.2 ± 0.2
**7**	52.5 ± 6.7	37.4 ± 7.4	9.0 ± 2.6	1.1 ± 0.5

The activation of cell death via the apoptotic pathway by copper complexes has been previously described.^39,55,59^ Based on Figure 7 and Table 6, it can also be observed that the % of necrotic cells was relatively low in all the conditions studied, except in the presence of Dox (∼10%), a result that is also in accordance with the literature.^60^

To further understand if the Cu(II) complexes were able to trigger apoptosis via the intrinsic pathway (namely, via BAX), the BAX/BCL-2 ratio was determined by Western Blot in HCT116DoxR cells after 48 h of exposure to the IC_50_ concentrations of complexes **2**, **3**, **5**, and **7** or 0.1% (v/v) DMSO (negative control). Since BAX is a pro-apoptotic protein, an increase in its expression compared to BCL-2 expression results in cell death by intrinsic apoptosis. Conversely, an increased expression of BCL-2 compared to BAX expression leads to cell survival.^61^ Thus, when the BAX/BCL-2 ratio is higher than 1, there is induction of apoptosis via BAX, and there is no involvement of this protein in this process when the ratio is equal to or lower than 1.^55^

**Figure 8:**
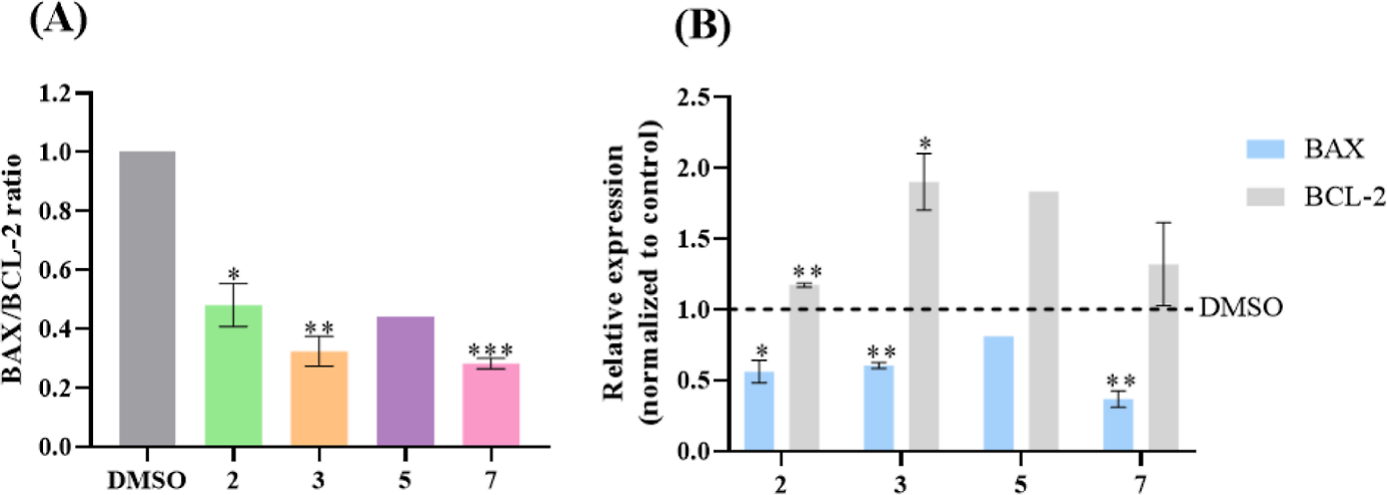
BAX and BCL-2 protein expression in HCT116DoxR cells after 48 h of incubation with the IC_50_ of the complexes **2**, **3**, **5**, and **7** or with 0.1% (v/v) DMSO. (A) Relative protein expression levels of BAX and BCL-2. (B) BAX/BCL-2 ratio. Results obtained were normalized against the DMSO control after an initial normalization with β-actin. Data are represented as the mean ± SEM of at least two independent biological assays. Statistical significance was determined relative to the DMSO control using the *t*-test (*p < 0.05; **p < 0.01; ***p < 0.001).

The expression levels of BAX, BCL-2, and β-actin for each Cu(II) complex are shown in Figure S19. The expression levels of BAX and BCL-2 in cells incubated with the Cu(II) complexes were first normalized to the respective β-actin levels and then compared to cells exposed only to DMSO control, being the ratios obtained as presented in Figure 8A.

It is possible to observe that the expression of BAX after exposure to complexes **2**, **3**, **5**, and **7** decreases compared to the DMSO control, while the expression of BCL-2 increases (Figure 8B). In this regard, the BAX/BCL-2 ratio is lower than 1 for all the complexes, indicating that apoptosis triggered by the complexes does not occur via the intrinsic pathway (via BAX), leading to the possibility that it may occur via the extrinsic pathway.

### Caspase-8 Activity

To assess whether apoptosis induction occurs by the extrinsic pathway, the activity of caspase-8, an initiator of this pathway, was analyzed in HCT116DoxR cells after 48 h of exposure to the IC_50_ of complexes **2**, **3**, **5**, and **7**. 0.1% (v/v) DMSO was used as the negative control and cisplatin (5 μM) as the positive control.

In this assay, the chromogenic substrate IETD-pNA, resulting from the conjugation between the peptide IETD (Ile-Glu-Thr-Asp) and the chromophore pNA (*p*-nitroanilide), is cleaved by caspase-8, releasing the chromophore, which is detected by measuring absorbance at 400 nm.^48^ The results obtained for each Cu(II) complex and cisplatin were normalized to those obtained for DMSO to assess whether there is an increase in caspase-8 activity in its presence.

**Figure 9:**
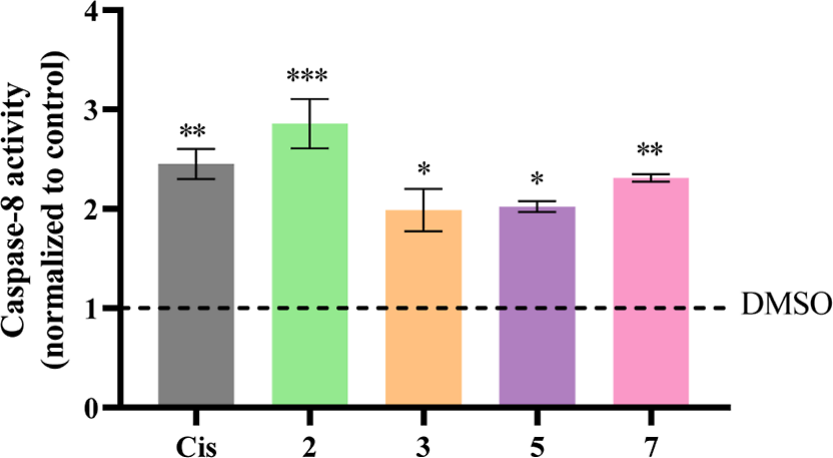
Caspase-8 activity in HCT116DoxR cells after 48 h of exposure to the IC_50_ concentrations of complexes **2**, **3**, **5**, and **7**. 0.1% (v/v) DMSO was used as a negative control and cisplatin (Cis, 5 μM) as a positive control. Results were normalized against the DMSO control (dashed). Data are represented as the mean ± SEM of two independent biological assays. Statistical significance was determined relative to the DMSO control by the one-way ANOVA method (*p < 0.05; **p < 0.01; ***p < 0.001).

According to the results obtained in Figure 9, the exposure of HCT116DoxR cells to the Cu(II) complexes or cisplatin results in a 2- to 3-fold increase in the levels of caspase-8 activity compared to the vehicle control (DMSO). These results seem to indicate that apoptosis induction occurred by the extrinsic pathway, which agrees with the previous results (Figures 7 and 8).

### Evaluation of Mitochondrial Membrane Potential (Δ**Ψ**_m_) in HCT116DoxR Cell Line by Flow Cytometry

To confirm the previous conclusions, the effect of the complexes on the Δ**Ψ**_m_ was analyzed. The induction of apoptosis by the intrinsic pathway or by the extrinsic pathway via BID can lead to the permeabilization of the mitochondrial membrane and subsequent release of cytochrome *c* into the cytoplasm, with a loss of Δ**Ψ**_m_.^62^ Thus, the study of this potential is an indicator of the functional state of the mitochondria and is important to determine whether the induction of apoptosis via the extrinsic pathway previously detected, occurs via BID and therefore with an impairment of mitochondrial function.

**Figure 10:**
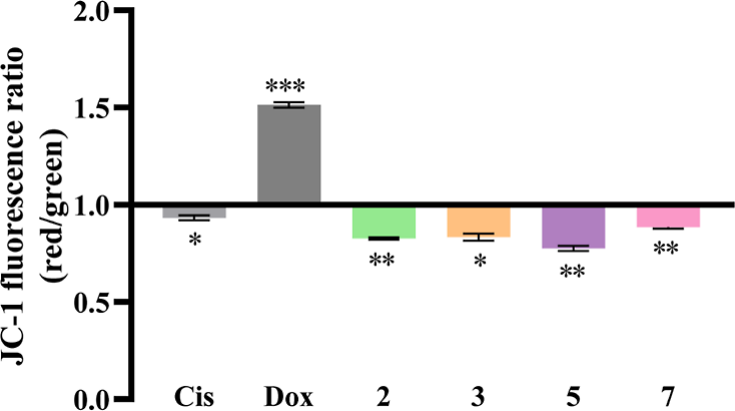
Ratio of JC-1 fluorescence (red/green) in HCT116DoxR cells after 48 h of exposure to the IC_50_ concentrations of complexes **2**, **3**, **5**, and **7**. 0.1% (v/v) DMSO was used as a negative control and cisplatin (Cis, 5 μM) and Dox (6 μM) as positive controls. Results were normalized against the DMSO control. Data are represented as the mean ± SEM of at least two independent biological assays. Statistical significance was determined relative to the DMSO control using the *t*-test (*p < 0.05; **p < 0.01; ***p < 0.001).

Therefore, the fluorescent probe JC-1 (5,5′,6,6′-tetrachloro-1,1′,3,3′-tetra-ethylbenzimidazolylcarbocyanine iodide) was used to study this potential since its aggregation state changes depending on the polarization state of the mitochondrial membrane. In healthy cells, with a high Δ**Ψ**_m_, this cationic probe enters and accumulates in the mitochondria, where it forms complexes and red fluorescence is emitted. However, in cases of loss of potential, the probe loses its ability to remain in the mitochondria and exits to the cytoplasm, where it accumulates in its monomeric form and emits green fluorescence.^55^ Thus, the ratio of red/green fluorescence intensities indicates repolarization (ratio > 1) or depolarization (ratio < 1) of the mitochondrial membrane (Figure 10).

Figure 10 shows that the exposure of HCT116DoxR cells to Dox results in a hyperpolarization of the mitochondrial membrane, an effect previously observed in this cancer cell line by other authors.^48^ It is also possible to observe that all complexes and cisplatin induce depolarization of the mitochondrial membrane, indicating that the loss of membrane potential occurs due to its permeabilization, resulting in the release of cytochrome *c* into the cytoplasm and activation of apoptosis. Intercalating this result with the results previously obtained, it is possible to conclude that apoptosis induction may occur by crosstalk between intrinsic and extrinsic pathways. When the extrinsic pathway is activated, caspase-8 cleaves and activates the pro-apoptotic protein BID, which becomes capable of activating the BAK protein, which induces the permeabilization of the mitochondrial membrane, releasing cytochrome *c* into the cytoplasm, which culminates in the activation of cell death by apoptosis.^63^

### Evaluation of Autophagy Induction in HCT116DoxR Cell Line by Flow Cytometry

In addition to analyzing the induction of apoptosis and necrosis in the presence of the Cu(II) complexes, the induction of autophagy was also studied. Autophagy is a type II programmed cell death mechanism characterized by the formation of autophagosomes, vesicles capable of fusing with lysosomes so that the cytosolic material they contain is degraded.^64,65^

In this assay, HCT116DoxR cells were exposed to the IC_50_ concentrations of complexes **2**, **3**, **5**, and **7** for 48 h. 0.1% (v/v) DMSO was used as the negative control and cisplatin (5 μM), Dox (6 μM), and rapamycin (1.5 μM) as positive controls. To analyze the induction of autophagy, a solution (green stain solution) with the ability to stain autophagic vesicles was used.

**Figure 11:**
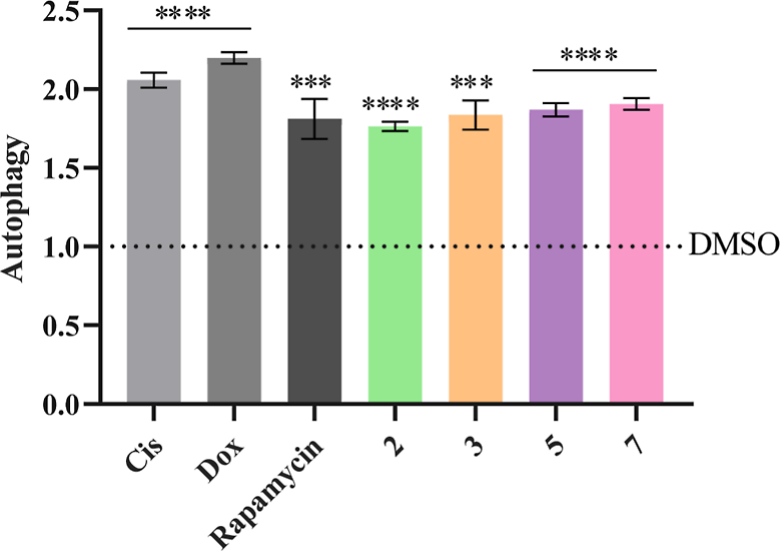
Induction of autophagy in HCT116DoxR cells after 48 h of exposure to the IC_50_ of complexes **2**, **3**, **5**, and **7**. 0.1% (v/v) DMSO was used as a negative control, while cisplatin (Cis, 5 μM), Dox (6 μM), and rapamycin (1.5 μM) were used as positive controls. Results were normalized against the DMSO control (dashed). Data are represented as the mean ± SEM of at least two independent biological assays. Statistical significance was determined relative to the DMSO control using the *t*-test (***p < 0.001; ****p < 0.0001).

The results obtained are shown in Figure 11 where it can be seen that both the positive controls and the Cu(II) complexes are capable of inducing autophagy, increasing the number of autophagic vesicles by ∼2× compared to the DMSO control. These results demonstrate that these four Cu(II) complexes induce cell death both by apoptosis (Figures 7–10) and autophagy (Figure 11), as already described for other Cu(II) complexes.^39^

### Production of Reactive Oxygen Species

Exposure to metal complexes can induce oxidative stress in cells by increasing the number of ROS, leading to disruption of normal cell function and activation of programmed cell death pathways such as apoptosis and autophagy.^39,48,55,66^

#### Quantification of Fluorescent Molecule DCF by Flow Cytometry

To quantify intracellular ROS levels by flow cytometry, the H_2_DCF-DA (2′,7′-dichlorohydrofluorescein diacetate) probe was used, which, after diffusing into the cells, is deacetylated by cellular esterases, originating a nonfluorescent compound, which, in the presence of ROS, is oxidized, resulting in the fluorescent molecule DCF (2′,7′-dichlorofluorescein), which can be detected by flow cytometry.^48^

**Figure 12:**
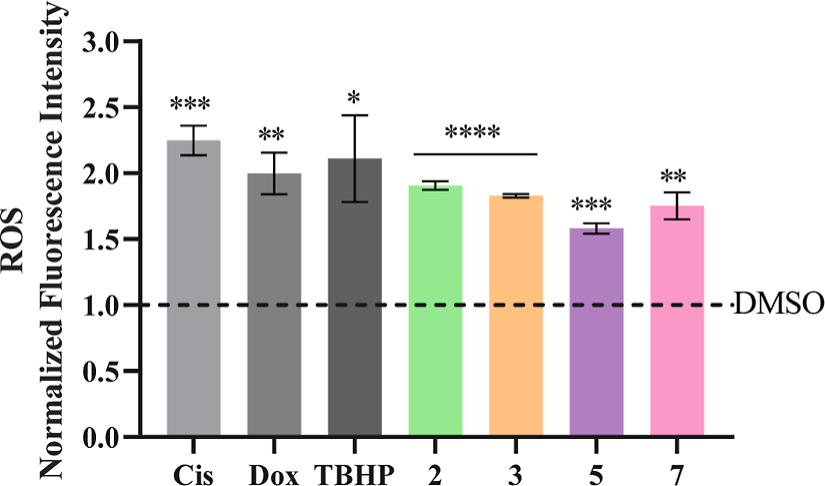
ROS production in HCT116DoxR cells after 48 h of exposure to the IC_50_ concentrations of complexes **2**, **3**, **5**, and **7**. 0.1% (v/v) DMSO was used as a negative control, while cisplatin (Cis, 5 μM), Dox (6 μM), and TBHP (42 μM) were used as positive controls. Results were normalized against the DMSO control (dashed). Data are represented as the mean ± SEM of at least two independent biological assays. Statistical significance was determined relative to the DMSO control using the *t*-test (*p < 0.05; **p < 0.01; ***p < 0.001; ****p < 0.0001).

Therefore, HCT116DoxR cells were incubated with H_2_DCF-DA after 48 h of exposure to the IC_50_ of complexes **2**, **3**, **5**, and **7**. 0.1% (v/v) DMSO was used as a negative control, while cisplatin (5 μM), Dox (6 μM), and TBHP (42 μM) were used as positive controls (Figure 12).

As observed in Figure 12, these Cu(II) complexes induce the production of ROS in HCT116DoxR cells, with an increase of at least 1.5× compared to the vehicle control (DMSO) at levels comparable to Dox control. The increased production of these species after exposure to copper complexes has been described by other authors^39,55^ and may be involved in the activation of cell death by apoptosis and autophagy seen above (Figures 7–11).

#### Determination of the DNA Cleavage Mechanisms by Copper Complexes In Vitro

Since the Cu(II) complexes were found to accumulate in the nucleus (Tables 4 and 5) and are capable of inducing the production of intracellular ROS (Figure 12), their ability to cleave pDNA in vitro was also assessed. For that, 100 ng of pUC18 were incubated for 24 h with increasing concentrations of each complex (5, 25, 50, 75, and 100 μM). Controls were performed with pUC18 in Tris-HCl buffer, pUC18 exposed to 1% (v/v) DMSO, and pUC18 incubated for 2 h with the restriction enzyme *Hin*dIII.

**Figure 13:**
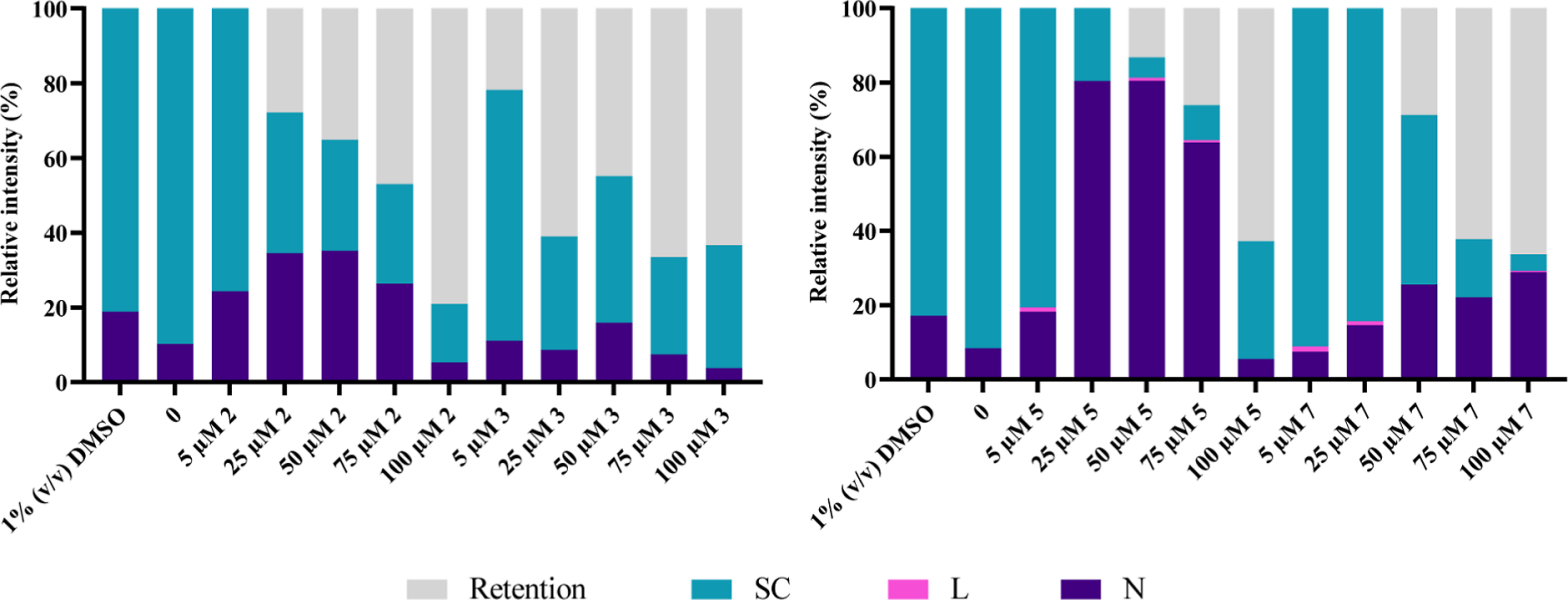
Relative intensity (as %) of pDNA isoforms [supercoiled (SC, blue bars), linear (L, pink bars), and nicked (N, purple bars)] or in the gel wells (gray bars) after 24 h of exposure to increasing concentrations (5–100 μM) of complexes **2**, **3**, **5**, and **7**. 0 in figure refers to pUC18 control (in 5 mM Tris-HCl and 50 mM NaCl pH = 7.0 buffer solution) and 1% (v/v) DMSO refers to pUC18 incubated in the presence of the complexes’ vehicle control (negative control).

Figure S20 shows the bands obtained after the samples were subjected to 0.8% (w/v) agarose gel electrophoresis, while Figure 13 shows the relative intensity of each pUC18 isoform obtained.

By analyzing Figure S20, three bands corresponding to the isoforms of the pUC18 plasmid were observed nicked (N), linear (L), and supercoiled (SC). The control samples (labeled 2, 3, and 14 in Figure S20) show bands corresponding to the N and SC isoforms, while the pUC18 sample exposed to *Hin*dIII activity (labeled 1) only shows a band corresponding to the L isoform, as expected due to the hydrolytic cleavage of the phosphodiester bond and used as a positive control for the L isoform.

Analyzing the pDNA samples exposed to increasing concentrations of each complex, some alterations can be detected in the plasmid isoforms relative to the controls. Looking at the relative intensity of each band, in Figure 13, we may observe that, in the case of complexes **2** and **3**, exposure to a concentration of 5 μM of complex results in a decrease in the relative intensity of the band corresponding to the SC isoform and an increase in the intensity of the band corresponding to the N isoform. It can therefore be concluded that these complexes can cleave pDNA into single strands, an interaction that reaches saturation at concentrations of 25 μM for complex **2** and 50 μM for complex **3**. On the contrary, exposure to a concentration of 5 μM of complexes **5** or **7**, apart from a decrease in the relative intensity of the band corresponding to the SC isoform and an increase of intensity of the band corresponding to the N isoform, results in the appearance of a band corresponding to the L isoform. Therefore, it can be concluded that these complexes are able to interact with pDNA and cleave it into single and double strands, an interaction that reaches saturation at concentrations of 50 μM complex **5** and 100 μM complex **7**. It can also be seen that increasing the concentration of each complex results in greater retention in the well, which is why the bands corresponding to the higher concentrations are more faded.

**Figure 14:**
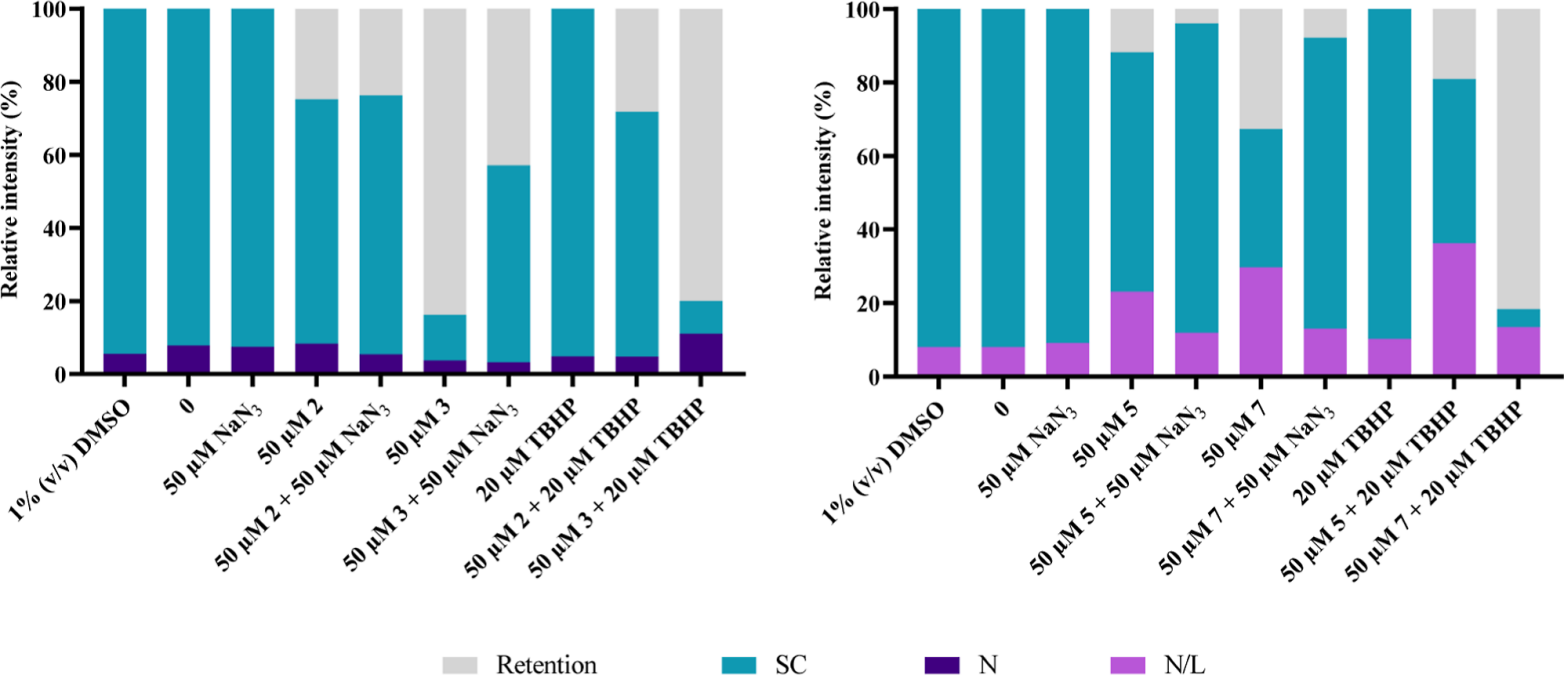
Relative intensity (%) of retention in the gel and of the supercoiled (SC), linear (L), and nicked (N) isoforms of pDNA after 24 h of exposure to 50 μM of NaN_3_, 20 μM of TBHP, 50 μM of complexes **2**, **3**, **5**, and **7**, and 50 μM of each complex and 50 μM of NaN_3_ or 20 μM of TBHP. 0 in figure refers to pUC18 control (in 5 mM Tris-HCl and 50 mM NaCl pH = 7.0 buffer solution) and 1% (v/v) DMSO refers to pUC18 incubated in the presence of the complexes’ vehicle control (negative control).

Having verified that the 4 complexes studied are capable of cleaving pDNA in vitro, the mechanism by which this cleavage occurs was determined by using sodium azide (NaN_3_), a singlet oxygen scavenger.^55^ TBHP was used as a positive control since it can interact with DNA and cleave it by oxidative mechanisms due to ROS.^67^ In this assay, a concentration of 50 μM of each complex was used since it was the concentration at which the greatest cleavage of the pDNA was detected with the lowest % of retention in the well. The gels obtained after the samples were subjected to 1% (w/v) agarose gel electrophoresis are shown in Figure S21, while Figure 14 shows the relative intensity of each pUC18 isoform obtained.

Analyzing Figures S21 and 14, we may observe that contrary to expectations as a positive control, TBHP was unable to induce pUC18 cleavage, showing an electrophoretic profile similar to the controls. This result can be explained by the fact that the concentration used (20 μM) was not sufficient for its effect. As expected, NaN_3_ had no effect on the pDNA, and the electrophoretic profile was similar to that of the controls. Given the high retention in the wells, it was not possible to distinguish the N and L isoforms in the gel for complexes **5** and **7**, the reason why only the changes in the intensity of the SC isoform were analyzed.

Analyzing the combination of each complex with NaN_3_, in the case of complexes **3**, **5**, and **7**, this combination results in an increase in the intensity of the band corresponding to the SC isoform compared to the complex alone, with the effect of the complexes on the pDNA being partially reversed for complex **3** and almost completely reversed for complexes **5** and **7** (Figure 14). Since NaN_3_ is a singlet oxygen scavenger, this result seems to indicate that the pDNA cleavage exerted by complexes **3**, **5**, and **7** occurs through the production of singlet oxygen. These results are in line with Cu(II) complexes’ ability to cleave DNA by oxidative mechanisms via a Fenton mechanism, as previously described.^68,69^ On the other hand, the electrophoretic profiles of complex **2** alone and in combination with NaN_3_ are practically identical. Therefore, we can conclude that NaN_3_ has no effect on the cleavage exerted by complex **2**, making it possible that the cleavage mechanism of this complex is associated with another type of oxygen radical, such as hydroxyl radicals or superoxide anions. These hypotheses could be confirmed by studying the effect of the combination of l-histidine, a scavenger of singlet oxygen and hydroxyl radicals, and ascorbic acid, a scavenger of superoxide anions, on the cleavage of pUC18 by complex **2**.^55^

The pDNA studies allow us to understand if a complex can interact with pDNA and is able to cleave it, usually by oxidative or hydrolytic mechanisms. Based on our gels, we can see that the supercoiled isoform disappears in the presence of the complexes, and the circular isoform or even the linear one appears—which is an indication of pDNA cleavage. The complexes, once reaching the cellular nucleus, can bind to DNA (noncovalently) and promote this cleavage directly via a Fenton mechanism^70^ or indirectly via the action of topoisomerase.^71^

### Cell Cycle Progression Analysis

The previous results and the ability of our Cu(II) complexes to enter the cell nucleus (Tables 4 and 5) and perform in vitro cleavage of pDNA (Figures 13 and 14) lead to the hypothesis that this damage could induce the activation of cell cycle checkpoints and the consequent arrest of the cell cycle for repair mechanisms. Ultimately, if cells are not able to repair this damage, cell death will be triggered (as already shown above, Figures 7–11). Therefore, we have assessed the cytostatic potential of complexes **2**, **3**, **5**, and **7**, and their ability to interfere with the progression of the cell cycle using the fluorescent marker PI. Since this marker can intercalate with DNA, the measurement of the fluorescence emitted by flow cytometry makes it possible to assess the DNA content at each stage of the cell cycle (G_0_/G_1_, S, and G_2_/M).^67^ To ensure that all the cells were in the same phase before their exposure to the complexes, a double block was carried out with thymidine, a DNA synthesis inhibitor.^67^

Thus, after being blocked in the S phase with thymidine, the HCT116DoxR cells were exposed for 9, 12, 18, and 24 h to the IC_50_ concentrations of complexes **2**, **3**, **5**, and **7**. 0.1% (v/v) DMSO (negative control) and 5 μM of cisplatin and 6 μM of Dox (positive controls).

**Figure 15:**
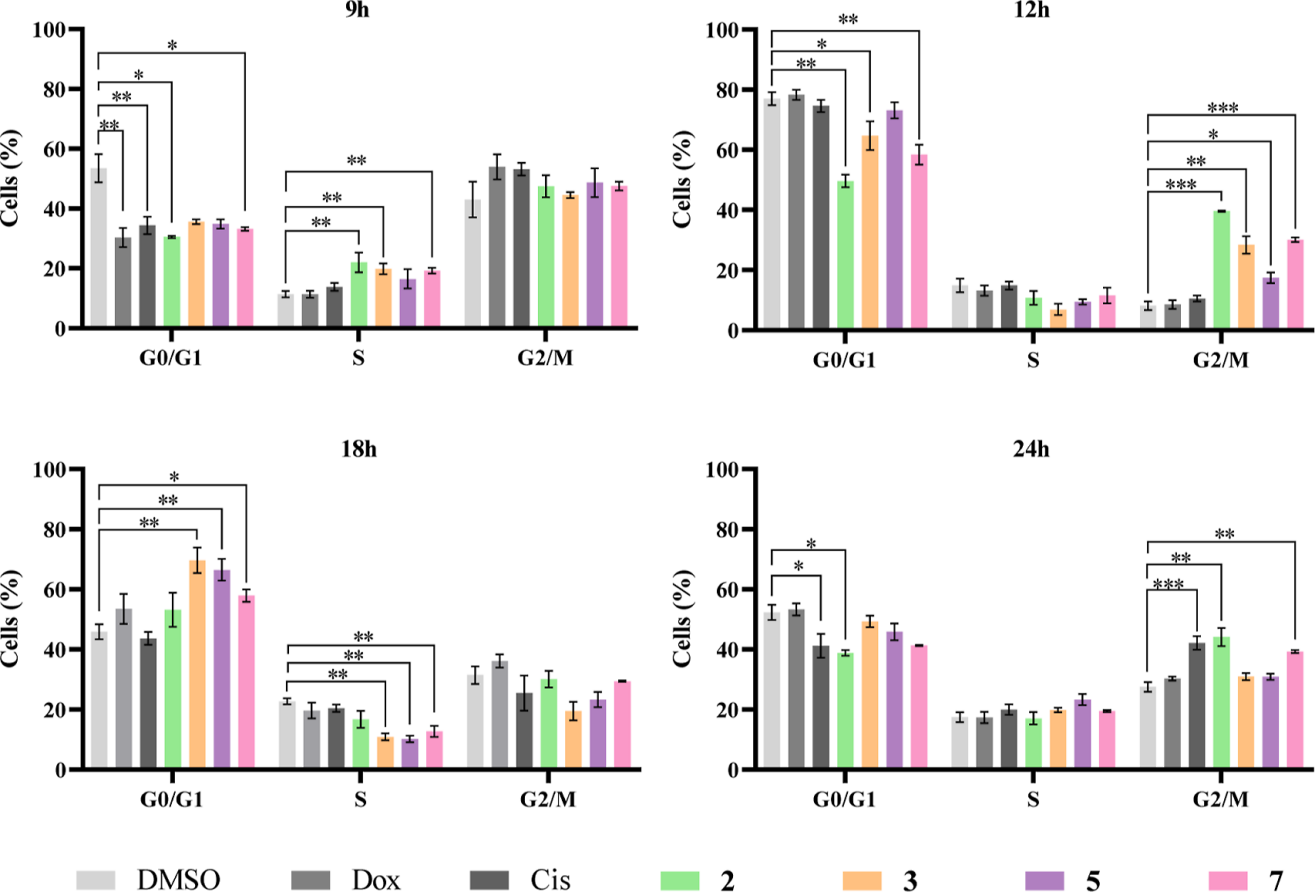
Cell cycle progression in HCT116DoxR cells after 9, 12, 18, and 24 h of exposure to the IC_50_ of complexes **2**, **3**, **5**, and **7**. 0.1% (v/v) DMSO was used as a negative control, while cisplatin (Cis, 5 μM) and Dox (6 μM) were used as positive controls. Data are represented as the mean ± SEM of at least two independent biological assays. Statistical significance was determined relative to the DMSO control using the *t*-test (*p < 0.05; **p < 0.01; ***p < 0.001).

Analyzing Figure 15, the exposure of the HCT116DoxR cells to complexes **2**, **3**, **5**, and **7** for 12 h delays cell cycle progression in the G_2_/M phase, resulting in a significantly higher % of cells in this phase than that observed for the negative control DMSO. At 18 h, the % of cells in the S phase after exposure to complexes **3**, **5**, and **7** is significantly lower than the value obtained for DMSO, indicating that these complexes induce a delay in cycle progression in the G_0_/G_1_ phase, in which the % of cells is higher than the control. Lastly, at 24 h, cisplatin and complexes **2** and **7** were found to delay the cycle in the G_2_/M phase. This shows that the complexes have a cytostatic effect, being able to delay the normal progression of the cell cycle in both the G_0_/G_1_ and G_2_/M phases, results that are in line with the hypothesis raised above and another evidence of their accumulation in the cell nucleus. Regarding the Dox control, it was expected that it would induce cell cycle arrest in the G_2_/M^72^ phase, which was not verified. This result may be a consequence of the resistance to Dox of the cell line under study, where it induced an increase of expression of the P-glycoprotein (PgP) efflux pump,^47^ being that the concentration used (6 μM) was not sufficient for the cycle arrest to be seen.

### Cellular Senescence Analysis

**Figure 16:**
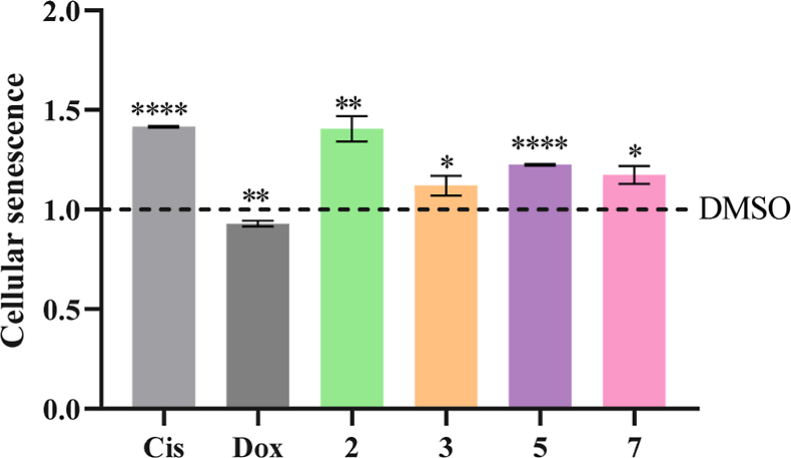
Induction of cellular senescence in HCT116DoxR cells after 48 h of exposure to the IC_50_ of complexes **2**, **3**, **5**, and **7**. 0.1% (v/v) DMSO was used as a negative control, while cisplatin (Cis, 5 μM) and Dox (6 μM) were used as positive controls. Results were normalized against the DMSO control (dashed). Data are represented as the mean ± SEM of two independent biological assays. Statistical significance was determined relative to the DMSO control using the *t*-test (*p < 0.05; **p < 0.01; ****p < 0.0001).

Given the ability of the Cu(II) complexes to interfere with the progression of the cell cycle, the senescence of HCT116DoxR cells was analyzed after 48 h of exposure to their IC_50_ concentrations. Premature cellular senescence is an irreversible state of cell cycle arrest that occurs in response to cellular stimuli that cause oxidative stress, DNA damage, or lack of nutrients.^73,74^ The induction of senescence can function as an antitumor mechanism as it prevents the proliferation of potentially carcinogenic cells.^74^ To study the senescence-inducing capacity of the complexes, the activity of β-galactosidase, whose increased expression is associated with senescence, was quantified by flow cytometry (Figure 16).^73^

Figure 16 shows that cisplatin and the Cu(II) complexes induce cellular senescence compared to the DMSO control. Contrary to expectations,^73^ exposure of HCT116DoxR cells to 6 μM of Dox did not induce an increase in cellular senescence, confirming the previous result, in which it was found that this antitumor agent did not induce cell cycle arrest, indicating that the concentration used (6 μM) is not sufficient for Dox to be considered a positive control in these studies.

### BSA Binding Studies

Considering a future in vivo systemic delivery to tumor cells, human plasma is enriched in proteins, namely, albumin.^75^ In this regard, the binding capability of Cu(II) complexes with albumin (namely, bovine serum albumin, BSA) was studied since it is a more easily accessible protein and has a structure with 76% similarity to human serum albumin, the protein generally used in these studies due to its high abundance in blood plasma and affinity for different ligands, drugs, and metabolites.^75^

**Figure 17:**
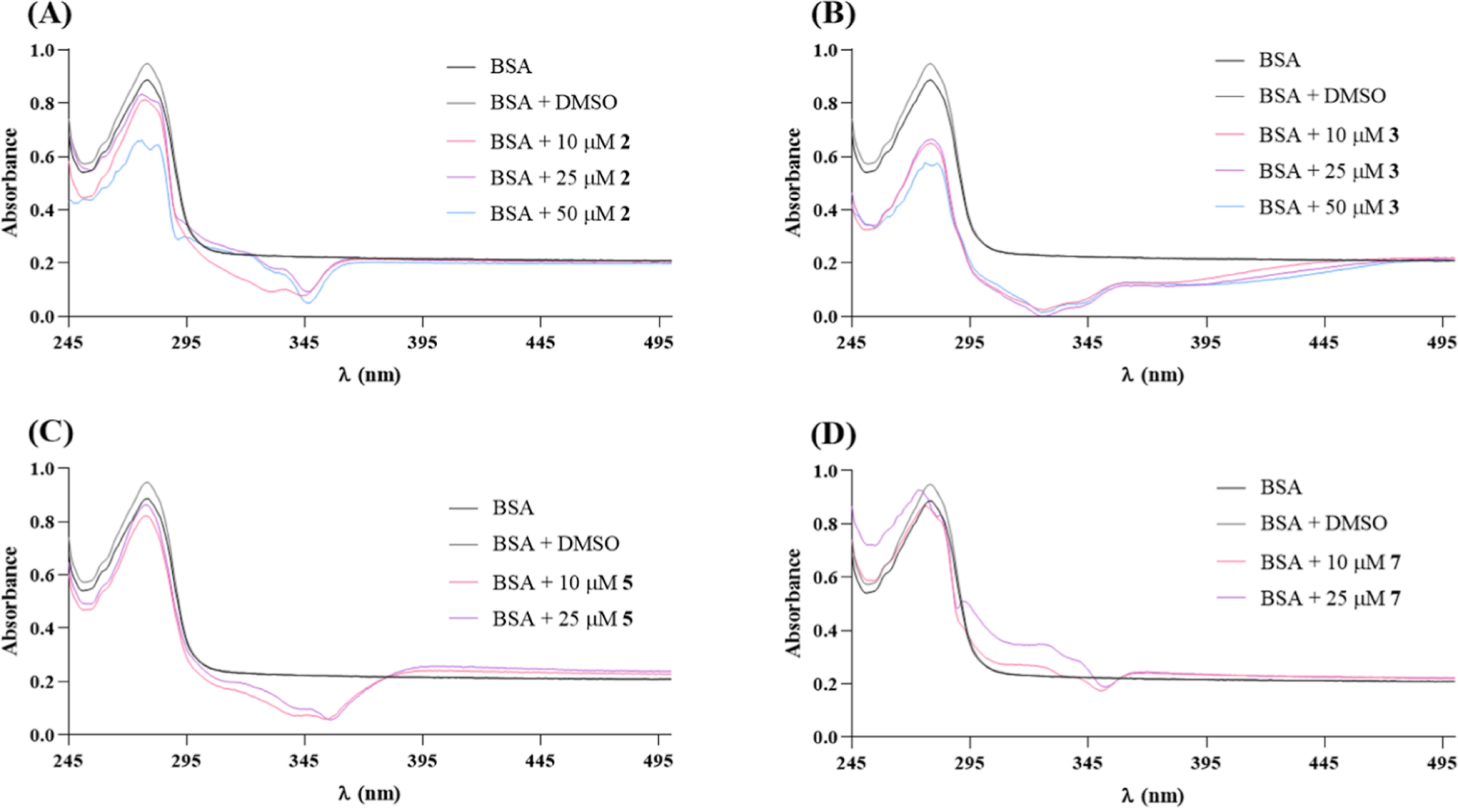
UV–visible spectra of BSA in the absence and presence of DMSO or increasing concentrations of copper complexes **2** (A), **3** (B), **5** (C), and **7** (D) after 24 h of exposure. Results were normalized to the absorbance of each complex alone and so that there were no negative absorbance values.

**Figure 18:**
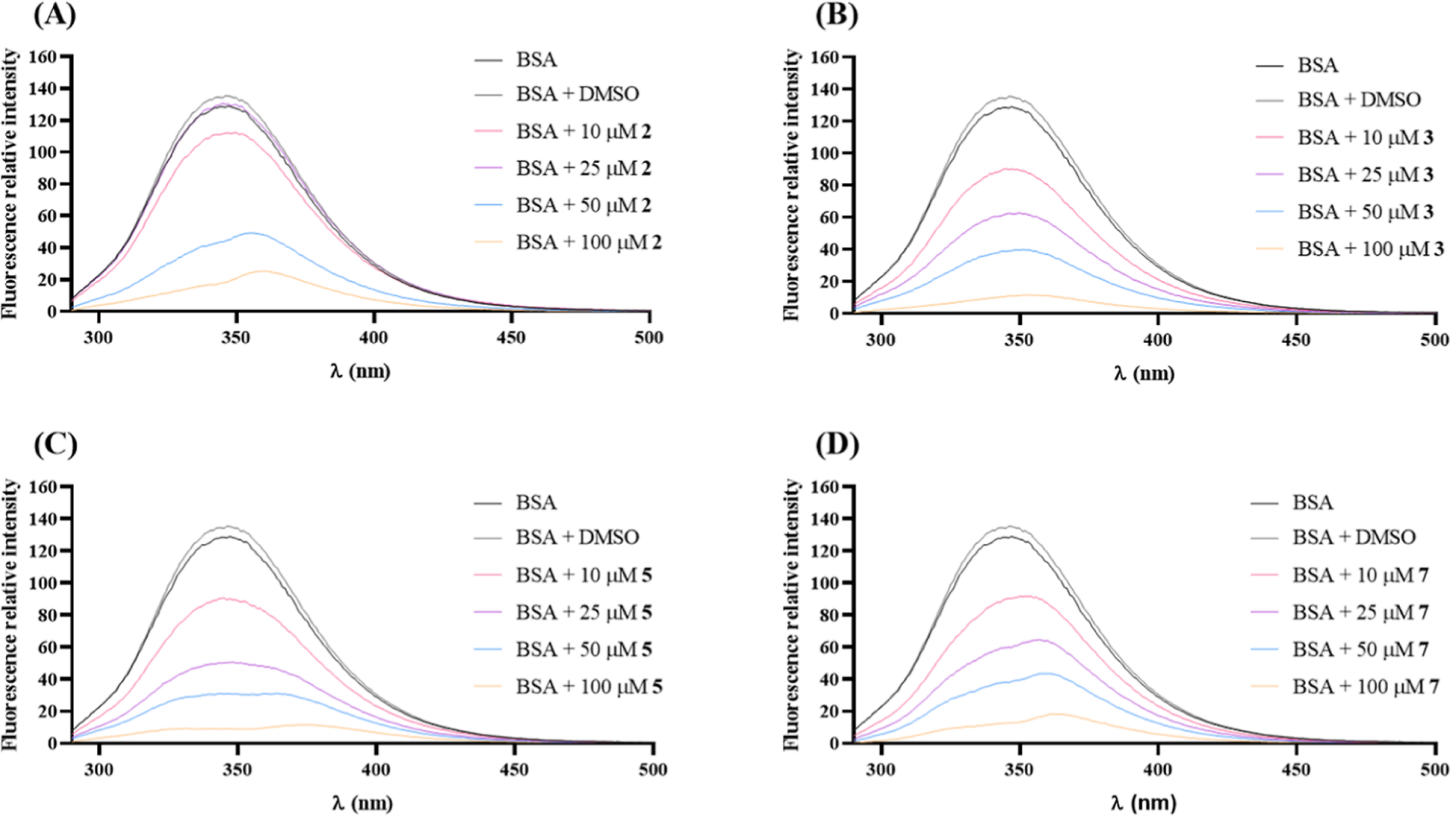
Effect of complexes **2** (A), **3** (B), **5** (C), and **7** (D) in BSA fluorescence after 24 h of exposure. Emission spectra (λ_exc_ = 278 nm) of BSA in the absence and presence of 1% (v/v) DMSO or increasing concentrations of copper complexes in 1% (v/v) DMSO and filtered buffer (10 mM phosphate and 150 mM NaCl) pH = 7.0.

Given that BSA has two tryptophan residues that possess intrinsic fluorescence,^75^ the interaction between the complexes **2**, **3**, **5**, and **7**, and this protein was characterized in vitro by UV–visible (Figure 17) and fluorescence spectroscopies (Figure 18).

As can be seen in Figure 17, BSA presents an absorption peak at 280 nm that reflects the absorbance of its aromatic amino acids (tryptophan, tyrosine, and phenylalanine).^75^ The complexes **2**, **3**, **5**, and **7** show absorbance peaks at the same wavelength. However, exposure to increasing concentrations of complexes results in lower absorbance, which seems to reveal the ability of these complexes to interact with BSA. Regarding complex **7**, there is a shift in the absorption peak at the maximum concentration studied, meaning that the microenvironment around the aromatic amino acids of the BSA protein has been affected.

To confirm the occurrence of interactions between the complexes and BSA, a fluorescence spectroscopy test was also carried out. Figure 18 shows that the complexes cause an intense decrease in the fluorescence emitted by BSA, proving their ability to interact with this protein. For instance, the binding constant (*K*_b_) for **2** is 1.23 × 10^7^ M^–1^ using the Stern–Volmer equation,^76^ which relates the fluorescence of protein in the absence and presence of the complexes (considering a 1:1 binding). The binding value for the complex **2** is comparable to others already seen in the literature.^77,78^ Given that BSA is an important transporter of ligands and drugs, these results may indicate that this protein will be able to transport these complexes in the bloodstream until they reach their target.

### Calf Thymus DNA Binding Studies

As previously demonstrated in the cell internalization and DNA cleavage studies, the tested Cu(II) systems are detected in the cells’ nucleus and can cleave pDNA due to their redox activity. To further determine the interaction mode of complexes **2**, **3**, **5**, and **7** with the DNA helix, UV–visible calf thymus DNA titration and ethidium bromide (EB) displacement fluorescence assays were performed.

**Figure 19:**
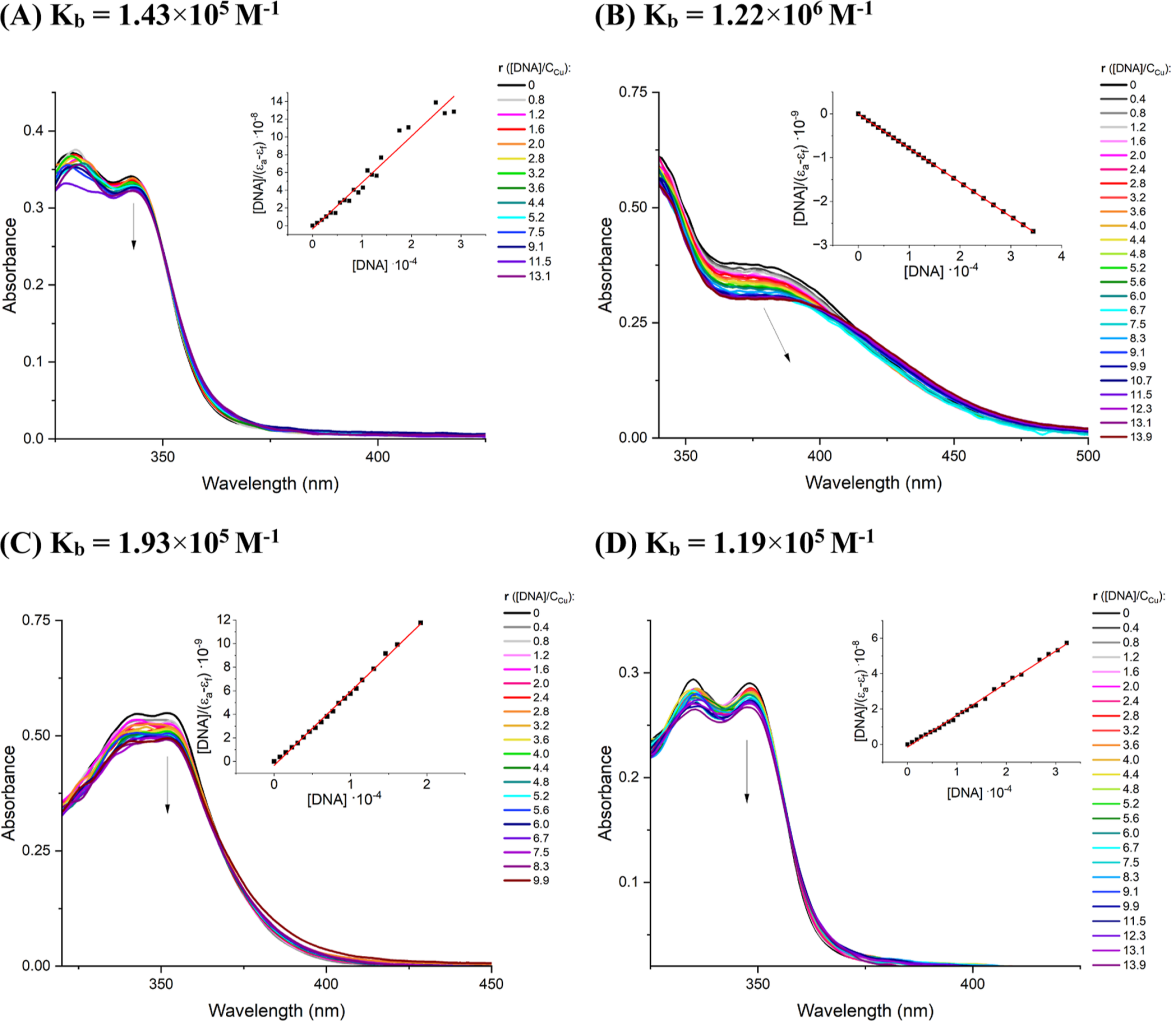
UV–vis spectra of PBS solution of complexes **2** (10 μM) (A), **3** (25 μM) (B), **5** (10 μM) (C), and **7** (10 μM) (D) in the absence and presence of increasing amounts of ct-DNA. Arrows show the changes in the Cu-based drug bands upon increasing amounts of ct-DNA. Inset shows the [DNA]/(ε_a_ – ε_f_) vs [DNA] plots for corresponding complexes.

The titration plots of **2**, **3**, **5**, and **7** versus ct-DNA are presented in Figure 19. The successive addition of ct-DNA to solutions of all examined Cu(II) complexes in PBS (pH 7.4, 130 mM NaCl) results in a decrease in the absorbance of Cu-based drug bands in the range 300–500 nm, with no alternations in the wavelength maxima for **2**, **5**, and **7** and a slight bathochromic shift in the absorption band for **3**. The hypochromic effect, accompanied by possible red-shift in absorption maxima, is typical of intercalative interactions of the Cu(II) complexes with DNA,^79,80^ likely due to the presence of the planar 2,2′:6′,2″-terpyridine moiety and aromatic substituent rings.

The magnitude of metallodrug/DNA interactions was estimated by employing the Wolfe–Shimer equation^81^ and plotting [DNA]/ε_a_ – ε_f_ versus [DNA] (insets linear plots in Figure 19). [DNA] represent the concentration of DNA, and ε_a_, ε_f_, and ε_b_ are extinction coefficients of the apparent, free, and bound metal complex, respectively. The intrinsic binding constants *K*_b_, obtained from the linear fit of the plot [DNA]/[ε_a_ – ε_f_] versus [DNA], decrease in the order: **3** (1.22 × 10^6^ M^–1^) ≫ **5** (1.93 × 10^5^ M^–1^) > **2** (1.43 × 10^5^ M^–1^) > **7** (1.19 × 10^5^ M^–1^), indicating a strong intercalative mode of binding to ct-DNA for **3** and weaker interactions in cases of **2**, **5**, and **7**.

To further confirm the intercalative interactions of complexes **2**, **3**, **5**, and **7** with DNA, EB displacement studies were conducted. EB is a phenanthradine derivative that efficiently intercalates into DNA, forming the adduct EB–ct-DNA with a strong emission at ∼620 nm. Equimolar solutions of ct-DNA and EB in PBS buffer were incubated and then titrated with increasing amounts of Cu-based drugs (from 0 to 40 μM). Changes in the fluorescence emission of the adduct EB–ct-DNA were monitored by fluorescence spectroscopy.

**Figure 20:**
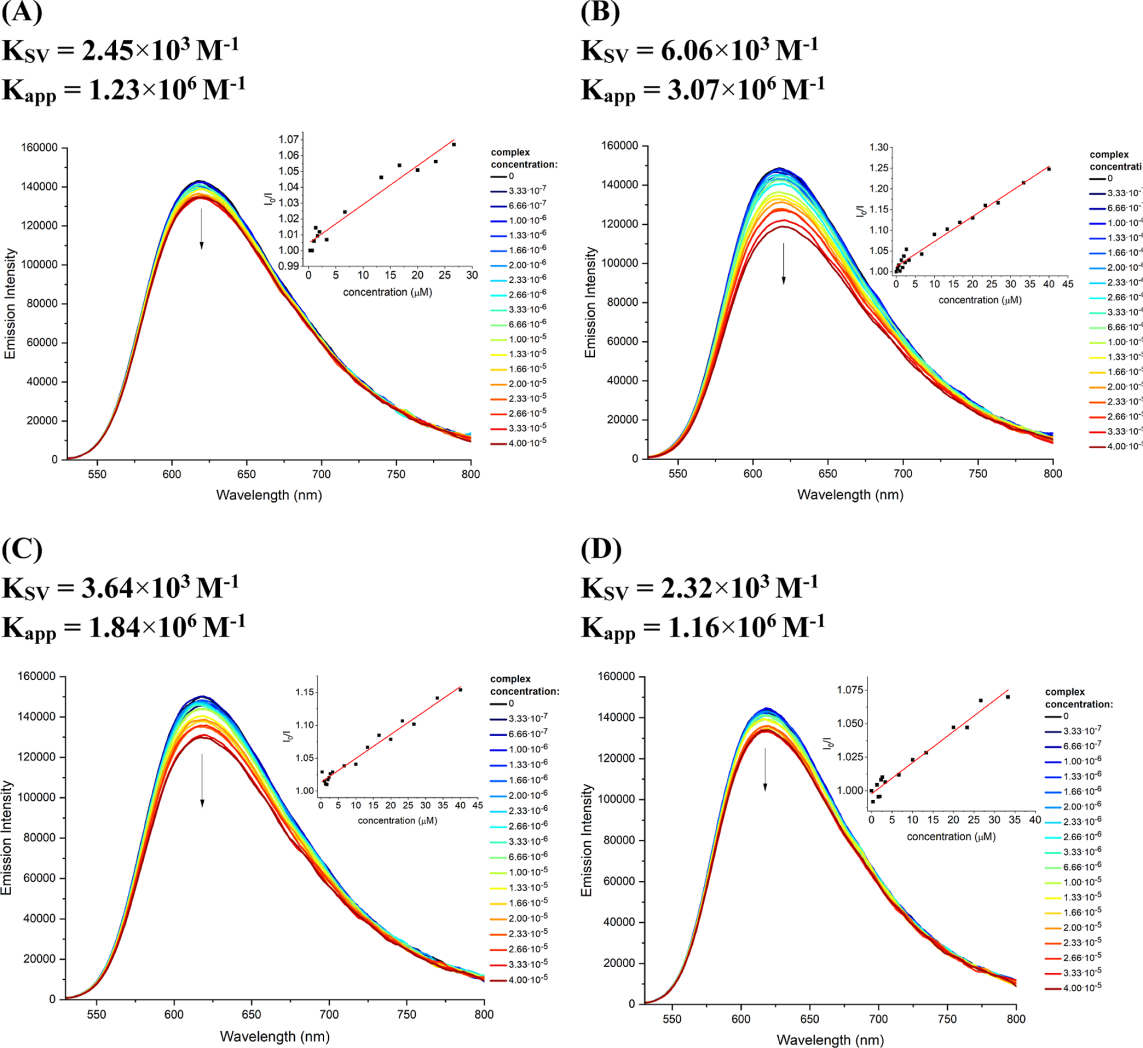
Emission spectra (λ_ex_ = 515 nm) of EB–ct-DNA in PBS buffer with the increasing concentration of **2** (A), **3** (B), **5** (C), and **7** (D) (0–40 μM). Insets show the Stern–Volmer plots for the corresponding complexes.

For all examined complexes, a noticeable decrease in the fluorescence intensity of the adduct EB–ct-DNA was observed upon the addition of Cu(II) complexes (Figure 20), and the fluorescence quenching is consistent with the linear Stern–Volmer equation^82,83^*I*_0_/(*I* = 1+ *K*_SV_ [*Q*]), where *I*_0_ and *I* are the fluorescence intensity in the absence and presence of the complexes and *K*_SV_ is the Stern–Volmer quenching constant. These spectral features are rationalized by the ability of complexes **2**, **3**, **5**, and **7** to release EB from the EB–ct-DNA adduct, supporting their intercalative interactions with ct-DNA. Regarding the apparent binding constants (*K*_app_), estimated by employing equation *K*_app_ [*Q*_1/2_] = *K*_EB_ [EB], where [*Q*_1/2_] is the concentration of the complex causing a 50% reduction in the fluorescence intensity, and *K*_EB_ = 1 × 10^7^ M^–1^, the tendency to replace EB from EB–ct-DNA adduct decreases in the order: **3** (3.07 × 10^6^ M^–1^) ≫ **5** (1.84 × 10^6^ M^–1^) > **2** (1.23 × 10^6^ M^–1^) > **7** (1.16 × 10^6^ M^–1^). This sequence aligns with those observed for the intrinsic binding constants *K*_b_ and Stern–Volmer quenching constant *K*_SV_. All these values clearly indicate that [Cu_2_Cl_2_(R-terpy)_2_](PF_6_)_2_ with a more π-conjugated 4-methoxy-1-naphthyl substituent (**3**) intercalates into DNA more efficiently than complexes **2**, **5**, and **7**. The stronger intercalative behavior of **3** compared to other tested drugs (**2**, **5**, and **7**) correlates well with its higher internalization and cytotoxicity toward HCT116DoxR in both 2D and 3D cell cultures.

### Cell Migration Assay

Cell migration is essential for the progression of a tumor as it is one of the main events leading to metastasis, the main cause of cancer death. Therefore, there is an intense search for new drugs that have good antimetastatic potential and do not enhance cell migration or, ideally, inhibit it.^84^

**Figure 21:**
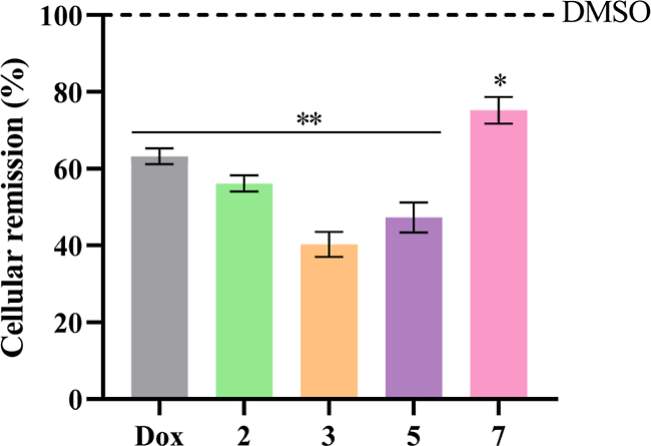
Fibroblast cell migration (%) after 24 h of exposure to the IC_50_ of complexes **2**, **3**, **5**, and **7** or 0.4 μM of Dox. 0.1% (v/v) DMSO was used as a vehicle control. Results were normalized against the DMSO control (dashed). Data are represented as the mean ± SEM of two independent biological assays. Statistical significance was determined relative to the DMSO control using the *t*-test (*p < 0.05; **p < 0.01).

A wound healing assay was therefore carried out on fibroblasts to study the influence of the Cu(II) complexes on cell migration in vitro. Skin fibroblasts were the model chosen for this assay as they are essential for regenerating damage in this tissue.^85^ In this study, immediately before replacing the medium in the wells with the IC_50_ of complexes **2**, **3**, **5**, and **7**, 0.1% (v/v) DMSO (vehicle control) or 0.4 μM of Dox, a scratch was made in the cell layer to form a cell-free region. The width of the scratch was measured at 0 h (when the complexes, DMSO or Dox were added) and after 24 h of incubation, and the % of cell remission was calculated (Figure 21).

The results obtained (Figure 21) suggest that all the Cu(II) complexes are capable of delaying fibroblast migration, in the order **3** > **5** > **2** > **7**, resulting in a lower % of remission compared to the vehicle control (DMSO). Moreover, the % of remission of **2**, **3**, and **5** is even lower compared to the positive control Dox. This result is in line with the literature, where the ability of other copper complexes to delay the migration of cells derived from human breast carcinoma was verified.^86,87^

### Ex-ovo Chick Chorioallantoic Membrane (CAM) Assay

Another relevant characteristic of an anticancer drug is its ability to inhibit angiogenesis, the process of forming new blood vessels from pre-existing ones, as this is crucial for the development and growth of a tumor. An increase in the number of blood vessels in the vicinity of a tumor allows cancer cells to acquire the oxygen and nutrients essential for their development and invade adjacent sites, developing metastases.^88^

**Figure 22:**
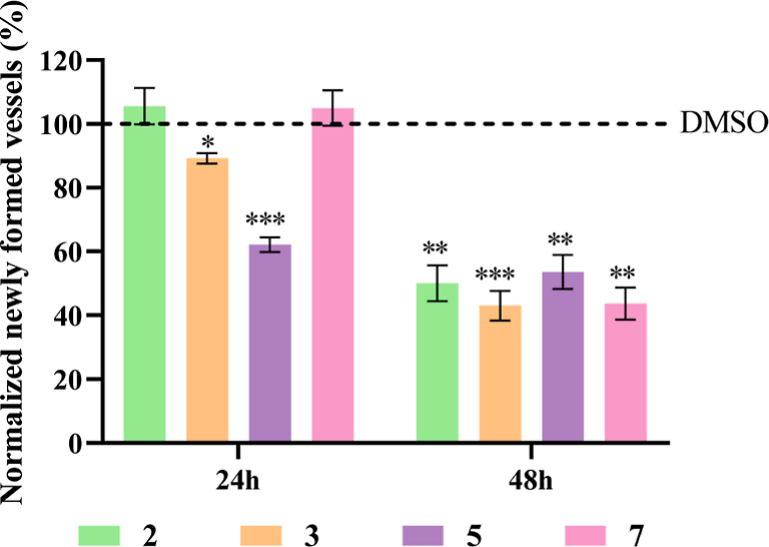
Formation of new blood vessels (%) after exposure of chicken embryos to the IC_50_ of complexes **2**, **3**, **5**, and **7** for 24 and 48 h. 0.1% (v/v) DMSO in PBS 1× was used as a negative control. Results were normalized to the number of tertiary veins at 0 h and the number obtained after incubation with the DMSO control at the respective incubation time and in the same embryo. Dashed line represents the value of the DMSO sample normalized to the respective number of blood vessels at 0 h. Data are represented as the mean ± SEM of at least four chicken embryos (independent biological assays). Statistical significance was determined relative to the DMSO control using the *t*-test (*p < 0.05; **p < 0.01; ***p < 0.001).

The ex-ovo CAM assay was therefore used to verify the interference of the Cu(II) complexes in angiogenesis. The CAM is an extraembryonic membrane with a high density of blood and lymphatic vessels, serving as a gas exchange surface.^89^ The results of the quantification of the blood vessels in this membrane before and after exposure to the IC_50_ concentrations of the complexes and 0.1% (v/v) DMSO in PBS 1× (negative control) for 24 and 48 h are shown in Figure 22.

After 24 h of exposure, complexes **3** and **5** present an antiangiogenic potential, reducing the % of blood vessels formed compared to the control. Interestingly, after 48 h of exposure, this potential is maintained, with a greater decrease in the % of blood vessels formed. On the other hand, complexes **2** and **7** showed no pro- or antiangiogenic effect after 24 h of exposure. However, at 48 h, there was an observed antiangiogenic effect from these complexes. Together with the results obtained before (Figure 21), these results reveal the potential of these complexes, particularly **3** and **5** as antimetastatic and antiangiogenic.

Importantly, this in vivo model is highly relevant as it also allows us to study the in vivo embryotoxicity of our complexes.^39,48,90^ Indeed, after 48 h of incubation of the complexes with the chicken embryo, no lethality was observed in any of the biological replicates, demonstrating that the complexes do not appear to be toxic (at these IC_50_ concentrations) to the chicken embryo.

## Conclusions

Within this work, the anticancer properties of Cu(II) complexes with the general formula [Cu_2_Cl_2_(R-terpy)_2_](PF_6_)_2_ were optimized through the incorporation of aromatic substituents (R) differing in π-conjugation, ring size, and presence or absence of various heteroatoms and methoxy groups. Among the studied complexes, those with 4-quinolinyl (**2**), 4-methoxy-1-naphthyl (**3**), 2-furanyl (**5**), and 2-pyridynyl (**7**) substituents exhibited high therapeutic potential for the HCT116DoxR cell line, with IC_50_ values significantly lower than the IC_50_ of Dox and high selectivity for this cell line. Interestingly, when the same study was performed in 3D models (spheroids), the IC_50_ values increased compared to those obtained for the 2D cultures due to the higher spheroid complexity and probable diffusion constraints. The observed IC_50_ values are particularly important when translating to mice xenograft studies, suggesting a more effective in vivo response. The IC_50_ values obtained for these complexes can be correlated with their internalization percentage, as they decrease as this percentage increases. The Cu(II) complexes were found to distribute throughout the cell, from the membrane and cytoskeleton to various organelles (mitochondria, endoplasmic reticulum, Golgi complex, and nucleus). While complex **2** showed high accumulation in the cell membrane, the other complexes were predominantly found in the nucleus, cytoskeleton, and Golgi complex.

These Cu(II) complexes induce cell death through both autophagy and apoptosis, involving the production of ROS. The activation of apoptosis may occur by crosstalk between intrinsic and extrinsic pathways. When the extrinsic pathway is activated, caspase-8 cleaves and activates the pro-apoptotic protein BID, which becomes capable of activating the BAK protein, inducing mitochondrial membrane permeabilization, and releasing cytochrome *c* into the cytoplasm, which culminates in the activation of cell death by apoptosis. The complexes showed cytostatic potential, delaying the cell cycle in the G_0_/G_1_ and G_2_/M phases and inducing senescence. Moreover, they demonstrated antimetastatic and antiangiogenic potential and were able to interact with BSA. Since albumin is a highly abundant protein in the blood, the ability of the complexes to interact with BSA implies that, once in the bloodstream, the complexes may bind to HSA (human serum albumin) and be delivered to tumor cells. No toxicity was observed in chicken embryos after 48 h of exposure to the IC_50_ of the complexes.

Based on these results, the four Cu(II) complexes exhibit promising antiproliferative potential in HCT116DoxR cells with no observed in vivo toxicity, making them suitable for further preclinical studies with other in vivo models.

## Experimental Section

### Materials and Methods

The chemicals and solvents used for the synthesis were of reagent grade, while the solvents for spectroscopic measurements were of HPLC grade. Copper(II) chloride dihydrate and ammonium hexafluorophosphate were purchased from Merck and used without further purification. 4′-Substituted 2,2′:6′,2″-terpyridines (R-terpy) were all previously reported^91−95^ and were prepared, according to the methods presented earlier,^96,97^ reacting 2-acetylpyridine and appropriate aldehyde (2:1 molar ratio) in the presence of aqueous ammonia. Detailed experimental conditions and methodology are described in the Supporting Information.

### Synthesis of Cu(II) Complexes **1–8**

CuCl_2_·2H_2_O (0.1 g, 0.6 mmol) dissolved in 5 mL of methanol was mixed with 0.1 g (0.6 mmol) of NH_4_PF_6_ dissolved in 5 mL of distilled water. The mixture was heated under reflux for 1 h to obtain a clear blue solution. After that, a methanolic solution of appropriate R-terpy ligand (0.6 mmol) was added, and the mixture was heated under reflux for another 6 h. The solution was allowed to evaporate in a hood at room temperature. The compounds were obtained as microcrystalline solids with varying colors of green—from deep dark green to green-blue in shade. Recrystallization from acetonitrile or methanol gave crystals suitable for X-ray analysis. All reported Cu(II) compounds **1**–**8** were >95% pure, as evidenced by elemental analysis, UPLC, and HRMS.

#### [Cu_2_Cl_2_(4′-(1-Naphthyl)-terpy)_2_](PF_6_)_2_ (**1**)

Yield: 30%. HR-ESI-MS calcd for C_25_H_17_N_3_ClCu^+^, 457.0407; found, 457.0415. Anal. Calcd for C_50_H_34_Cl_2_Cu_2_N_6_P_2_F_12_ (1206.77 g/mol): C, 49.76; H, 2.84; N, 6.96%. Found: C, 49.44; H, 3.04; N, 6.77%. IR (cm^–1^): 3114, 3078 (ν_C–H_); 1606, 1571, 1556 (ν_C=C_, ν_C=N_); 841 (ν_PF6_-), 558 (δ_PF6_-). UV–vis (DMSO 10^–5^ M/nm) (ε × 10^4^/M^–1^ × cm^–1^): 706 (0.02); 480 (0.2); 345 (2.8); 333 (2.8); 290 (4.4); 281 (4.0).

#### [Cu_2_Cl_2_(4′-(4-Quinolyl)-terpy)_2_](PF_6_)_2_ (**2**)

Yield: 31%. HR-ESI-MS calcd for C_24_H_16_N_4_ClCu^+^, 458.0359; found, 458.0367. Anal. Calcd for C_48_H_32_Cl_2_Cu_2_N_8_P_2_F_12_ (1208.75 g/mol): C, 47.70; H, 2.67; N, 9.27%. Found: C, 47.66; H, 2.93; N, 9.15%. IR (cm^–1^): 3117, 3070, 2923, 2851 (ν_C–H_); 1616, 1606, 1571, 1555 (ν_C=C_, ν_C=N_); 840 (ν_PF6_-), 558 (δ_PF6_-). UV–vis (DMSO 10^–5^ M/nm) (ε × 10^4^/M^–1^ × cm^–1^): 710 (0.02); 345 (2.4); 332 (2.7); 320 (2.3); 291 (3.9); 282 (3.7).

#### [Cu_2_Cl_2_(4′-(4-Methoxy-1-naphthyl)-terpy)_2_](PF_6_)_2_ (**3**)

Yield: 53%. HR-ESI-MS calcd for C_26_H_19_N_3_OClCu^+^, 487.0513; found, 487.0517. Anal. Calcd for C_52_H_38_Cl_2_Cu_2_N_6_O_2_P_2_F_12_·CH_3_CN (1307.87 g/mol): C, 49.59; H, 3.16; N, 7.50%. Found: C, 49.39; H, 3.22; N, 7.49%. IR (cm^–1^): 3115, 3076, 2965, 2940, 2843 (ν_C–H_); 1608, 1576, 1554 (ν_C=C_, ν_C=N_); 839 (ν_PF6_-), 558 (δ_PF6_-). UV–vis (DMSO 10^–5^ M/nm) (ε × 10^4^/M^–1^ × cm^–1^): 706 (0.02); 382 (1.1); 340 (2.2); 326 (2.7); 316 (2.6); 290 (3.6).

#### [Cu_2_Cl_2_(4′-(4,7-Dimethoxy-1-naphthyl)-terpy)_2_](PF_6_)_2_ (**4**)

Yield: 46%. HR-ESI-MS calcd for C_27_H_21_N_3_O_2_ClCu^+^, 517.0618; found, 517.0612. Anal. Calcd for C_54_H_42_Cl_2_Cu_2_N_6_O_4_P_2_F_12_ (1326.87 g/mol): C, 48.88; H, 3.19; N, 6.33%. Found: C, 48.60; H, 3.29; N, 6.67%. IR (cm^–1^): 3115, 3017, 2940, 2843 (ν_C–H_); 1612, 1570, 1549 (ν_C=C_, ν_C=N_); 839 (ν_PF6_-), 558 (δ_PF6_-). UV–vis (DMSO 10^–5^ M/nm) (ε × 10^4^/M^–1^ × cm^–1^): 709 (0.02); 392 (1.3); 328 (3.0); 290 (4.2); 278 (3.8); 267 (4.2).

#### [Cu_2_Cl_2_(4′-(2-Furanyl)-terpy)_2_](PF_6_)_2_ (**5**)

Yield: 30%. HR-ESI-MS calcd for C_19_H_13_N_3_OClCu^+^, 397.0043; found, 397.0053. Anal. Calcd for C_38_H_26_Cl_2_Cu_2_N_6_O_2_P_2_F_12_ (1086.58 g/mol): C, 42.00; H, 2.41; N, 7.73%. Found: C, 41.91; H, 2.40; N, 7.98%. IR (cm^–1^): 3167, 3120, 3086 (ν_C–H_); 1615, 1607, 1583, 1571, 1557 (ν_C=C_, ν_C=N_); 832 (ν_PF6_-), 556 (δ_PF6_-). UV–vis (DMSO 10^–5^ M/nm) (ε × 10^4^/M^–1^ × cm^–1^): 708 (0.02); 356 (4.0); 343 (4.0); 328 (3.0); 310 (2.9); 289 (3.7); 269 (3.2).

#### [Cu_2_Cl_2_(4′-(2-Thiophenyl)-terpy)_2_](PF_6_)_2_ (**6**)

Yield: 39%. HR-ESI-MS calcd for C_19_H_13_N_3_SClCu^+^, 412.9815; found, 412.9817. Anal. Calcd for C_38_H_26_Cl_2_Cu_2_N_6_S_2_P_2_F_12_ (1118.71 g/mol): C, 40.80; H, 2.34; N, 7.51%. Found: C, 40.77; H, 2.56; N, 7.76%. IR (cm^–1^): 3117, 3092, 3076 (ν_C–H_); 1606, 1574, 1558, 1525 (ν_C=C_, ν_C=N_); 836 (ν_PF6_-), 556 (δ_PF6_-). UV–vis (DMSO 10^–5^ M/nm) (ε × 10^4^/M^–1^ × cm^–1^): 708 (0.02); 354 (4.2); 343 (4.2); 290 (4.3); 271 (3.7).

#### [Cu_2_Cl_2_(4′-(2-Pyridynyl)-terpy)_2_](PF_6_)_2_ (**7**)

Yield: 45%. HR-ESI-MS calcd for C_20_H_14_N_4_ClCu^+^, 408.0203; found, 408.0205. Anal. Calcd for C_40_H_28_Cl_2_Cu_2_N_8_P_2_F_12_ (1108.63 g/mol): C, 43.34; H, 2.55; N, 10.11%. Found: C, 43.45; H, 2.75; N, 9.98%. IR (cm^–1^): 3103, 3070, 3015 (ν_C–H_); 1618, 1606, 1588, 1572, 1561 (ν_C=C_, ν_C=N_); 837 (ν_PF6_-), 556 (δ_PF6_-). UV–vis (DMSO 10^–5^ M/nm) (ε × 10^4^/M^–1^ × cm^–1^): 706 (0.02); 352 (1.9); 337 (2.2); 306 (3.1); 290 (6.2); 284 (5.9); 272 (4.6).

#### [Cu_2_Cl_2_(4′-(*N*-Ethyl-9*H*-carbazol-3-yl)-terpy)_2_](PF_6_)_2_ (**8**)

Yield: 31%. HR-ESI-MS calcd for C_29_H_22_N_4_ClCu^+^, 524.0829; found, 524.0825. Anal. Calcd for C_58_H_44_Cl_2_Cu_2_N_8_P_2_F_12_ (1340.95 g/mol): C, 51.95; H, 3.31; N, 8.36%. Found: C, 51.66; H, 3.26; N, 8.46%. IR (cm^–1^): 3120, 3051, 2981, 2931 (ν_C–H_); 1606, 1591, 1572, 1560 (ν_C=C_, ν_C=N_); 841 (ν_PF6_-), 558 (δ_PF6_-). UV–vis (DMSO 10^–5^ M/nm) (ε × 10^4^/M^–1^ × cm^–1^): 703 (0.02); 406 (3.5); 335 (4.0); 324 (4.3); 291 (8.4); 281 (7.9).

## Supporting Information Available

Experimental details; HRMS; UPLC traces; X-ray data; IR spectra of compounds and ligands; electronic absorption spectra in solution and in solid state, UV–vis stability, and solubility in DMSO, PBS, and RPMI; conductivity measurements; cell viability of HCT116, HCT116-DoxR, and A2780 and fibroblasts cell lines after exposure to different concentrations of the copper complexes, ligands, Dox, and cisplatin; fluorescence and confocal microscopy; Western Blot bands; and electrophoresis data (PDF)
Molecular formula strings (CSV)
Crystallographic Information file of complex **1** (CIF)
Crystallographic Information file of complex **2** (CIF)
Crystallographic Information file of complex **3** (CIF)
Crystallographic Information file of complex **4** (CIF)
Crystallographic Information file of complex **5** (CIF)
Crystallographic Information file of complex **6** (CIF)
Crystallographic Information file of complex **7** (CIF)
Crystallographic Information file of complex **8** (CIF)

## Supplementary Material

jm4c00119_si_002.csv

jm4c00119_si_003.cif

jm4c00119_si_004.cif

jm4c00119_si_005.cif

jm4c00119_si_006.cif

jm4c00119_si_007.cif

jm4c00119_si_008.cif

jm4c00119_si_009.cif

jm4c00119_si_010.cif

## Abbreviations

ΔΨ_m_: mitochondrial membrane potential
A2780: ovarian carcinoma cell line
CAM: chick chorioallantoic membrane
Cis: cisplatin
ct-DNA: calf thymus DNA
DCF: 2′7′-dichlorofluorescein
DMEM: Dulbecco’s Modified Eagle Medium
Dox: doxorubicin
EB: ethidium bromide
FBS: fetal bovine serum
H_2_DCF-DA: 2′,7′-dichlorohydrofluorescein diacetate
HCT116: colorectal carcinoma cell line
HCT116-DoxR: doxorubicin-resistant colorectal carcinoma cell line
HR-ESI-MS: high-resolution electrospray ionization mass spectrometry
ICP-AES: inductively coupled plasma atomic emission spectrometry
IETD: Ile-Glu-Thr-Asp
IL: intraligand π → π* transitions
ILCT: intraligand charge transfer transitions
JC-1: 5,5′,6,6′-tetrachloro-1,1′,3,3′ tetraethylbenzimidazolcarbocyanine iodide
LMCT: ligand-to-metal charge transfer
MTS: (3-(4,5-dimethylthiazol-2-yl)-5-(3-carboxymethoxyphenyl)-2-(4-sulfophenyl)-2*H*-tetrazolium)
PBS: Dulbecco’s Modified Phosphate Buffered Saline
pDNA: pUC18 plasmid DNA
Pen/Strep: penicillin/streptomycin
PgP: P-glycoprotein
PI: propidium iodide
pNA: *p*-nitroanilide
RPMI: Roswell Park Memorial Institute
R-terpy: 4′-substituted 2,2′:6′,2″-terpyridines
SI: selectivity index
terpy: 2,2′:6′,2″-terpyridine
UPLC: ultra performance liquid chromatography

## References

[ref1] Tardito S.; Marchio L. Copper Compounds in Anticancer Strategies. Curr. Med. Chem. 2009, 16 (11), 1325–1348. 10.2174/092986709787846532. 19355889

[ref2] Tabti R.; Tounsi N.; Gaiddon C.; Bentouhami E.; Desaubry L. Progress in Copper Complexes as Anticancer Agents. Med. Chem. 2017, 07 (05), 875–879. 10.4172/2161-0444.1000445.

[ref3] Nelson J. Copper. Annu. Rep. Sect. Inorg. Chem. 2010, 106 (0), 235–254. 10.1039/b918386h.

[ref4] Tisato F.; Marzano C.; Porchia M.; Pellei M.; Santini C. Copper in Diseases and Treatments, and Copper-Based Anticancer Strategies. Med. Res. Rev. 2010, 30 (4), 708–749. 10.1002/med.20174. 19626597

[ref5] Gupte A.; Mumper R. J. Elevated Copper and Oxidative Stress in Cancer Cells as a Target for Cancer Treatment. Cancer Treat Rev. 2009, 35 (1), 32–46. 10.1016/j.ctrv.2008.07.004. 18774652

[ref6] Xie H.; Kang Y. J. Role of Copper in Angiogenesis and Its Medicinal Implications. Curr. Med. Chem. 2009, 16 (10), 1304–1314. 10.2174/092986709787846622. 19355887

[ref7] Zuazo-Gaztelu I.; Casanovas O. Unraveling the Role of Angiogenesis in Cancer Ecosystems. Front. Oncol. 2018, 8, 248 10.3389/fonc.2018.00248. 30013950 PMC6036108

[ref8] Santini C.; Pellei M.; Gandin V.; Porchia M.; Tisato F.; Marzano C. Advances in Copper Complexes as Anticancer Agents. Chem. Rev. 2014, 114 (1), 815–862. 10.1021/cr400135x. 24102434

[ref9] McGivern T. J. P.; Afsharpour S.; Marmion C. J. Copper Complexes as Artificial DNA Metallonucleases: From Sigman’s Reagent to next Generation Anti-Cancer Agent?. Inorg. Chim. Acta 2018, 472, 12–39. 10.1016/j.ica.2017.08.043.

[ref10] Wu S.; Wu Z.; Ge Q.; Zheng X.; Yang Z. Antitumor Activity of Tridentate Pincer and Related Metal Complexes. Org. Biomol. Chem. 2021, 19 (24), 5254–5273. 10.1039/D1OB00577D. 34059868

[ref11] Musiol R.; Malecki P.; Pacholczyk M.; Mularski J. Terpyridines as Promising Antitumor Agents: An Overview of Their Discovery and Development. Expert Opin. Drug Discovery 2022, 17 (3), 259–271. 10.1080/17460441.2022.2017877. 34928186

[ref12] Gu Y.-Q.; Zhong Y.-J.; Hu M.-Q.; Li H.-Q.; Yang K.; Dong Q.; Liang H.; Chen Z.-F. Terpyridine Copper(Ii) Complexes as Potential Anticancer Agents by Inhibiting Cell Proliferation, Blocking the Cell Cycle and Inducing Apoptosis in BEL-7402 Cells. Dalton Trans. 2022, 51 (5), 1968–1978. 10.1039/D1DT02988F. 35023532

[ref13] Rani J J.; Roy S. Recent Development of Copper (II) Complexes of Polypyridyl Ligands in Chemotherapy and Photodynamic Therapy. ChemMedChem 2023, 18 (8), e202200652 10.1002/cmdc.202200652. 36773314

[ref14] Wang C.; Yang X.; Dong C.; Chai K.; Ruan J.; Shi S. Cu-Related Agents for Cancer Therapies. Coord. Chem. Rev. 2023, 487, 215156 10.1016/j.ccr.2023.215156.

[ref15] Abhijnakrishna R.; Magesh K.; Ayushi A.; Velmathi S. Advances in the Biological Studies of Metal-Terpyridine Complexes: An Overview From 2012 to 2022. Coord. Chem. Rev. 2023, 496, 215380 10.1016/j.ccr.2023.215380.

[ref16] Maity B.; Roy M.; Banik B.; Majumdar R.; Dighe R. R.; Chakravarty A. R. Ferrocene-Promoted Photoactivated DNA Cleavage and Anticancer Activity of Terpyridyl Copper(II) Phenanthroline Complexes. Organometallics 2010, 29 (16), 3632–3641. 10.1021/om100524x.

[ref17] Mahendiran D.; Gurumoorthy P.; Gunasekaran K.; Senthil Kumar R.; Rahiman A. K. Structural Modeling, in Vitro Antiproliferative Activity, and the Effect of Substituents on the DNA Fastening and Scission Actions of Heteroleptic Copper(II) Complexes with Terpyridines and Naproxen. New J. Chem. 2015, 39 (10), 7895–7911. 10.1039/C5NJ01059D.

[ref18] Deka B.; Sarkar T.; Banerjee S.; Kumar A.; Mukherjee S.; Deka S.; Saikia K. K.; Hussain A. Novel Mitochondria Targeted Copper(II) Complexes of Ferrocenyl Terpyridine and Anticancer Active 8-Hydroxyquinolines Showing Remarkable Cytotoxicity, DNA and Protein Binding Affinity. Dalton Trans. 2017, 46 (2), 396–409. 10.1039/C6DT03660K. 27929173

[ref19] Chu W.; Wang Y.; Liu S.; Yang X.; Wang S.; Li S.; Zhou G.; Qin X.; Zhou C.; Zhang J. Synthesis, Cytotoxicity and DNA-Binding Properties of Pd(II), Cu(II) and Zn(II) Complexes with 4′-(4-(2-(Piperidin-1-Yl)Ethoxy)Phenyl)-2,2′:6′,2″-Terpyridine. Bioorg. Med. Chem. Lett. 2013, 23 (18), 5187–5191. 10.1016/j.bmcl.2013.07.003. 23927970

[ref20] Liang J.-W.; Wang Y.; Du K.-J.; Li G.-Y.; Guan R.-L.; Ji L.-N.; Chao H. Synthesis, DNA Interaction and Anticancer Activity of Copper(II) Complexes with 4′-Phenyl-2,2′:6′,2″-Terpyridine Derivatives. J. Inorg. Biochem. 2014, 141, 17–27. 10.1016/j.jinorgbio.2014.08.006. 25172994

[ref21] Manikandamathavan V. M.; Thangaraj M.; Weyhermuller T.; Parameswari R. P.; Punitha V.; Murthy N. N.; Nair B. U. Novel Mononuclear Cu (II) Terpyridine Complexes: Impact of Fused Ring Thiophene and Thiazole Head Groups towards DNA/BSA Interaction, Cleavage and Antiproliferative Activity on HepG2 and Triple Negative CAL-51 Cell Line. Eur. J. Med. Chem. 2017, 135, 434–446. 10.1016/j.ejmech.2017.04.030. 28475971

[ref22] Tummalapalli K.; C.s V.; Munusami P.; Pathak M.; M.m B. Evaluation of DNA/Protein Interactions and Cytotoxic Studies of Copper(II) Complexes Incorporated with N, N Donor Ligands and Terpyridine Ligand. Int. J. Biol. Macromol. 2017, 95, 1254–1266. 10.1016/j.ijbiomac.2016.11.022. 27838416

[ref23] Zhang D.-Y.; Nie Y.; Sang H.; Suo J.-J.; Li Z.-J.; Gu W.; Tian J.-L.; Liu X.; Yan S.-P. Three Structurally Related Copper Complexes with Two Isomers: DNA/BSA Binding Ability, DNA Cleavage Activity and Excellent Cytotoxicity. Inorg. Chim. Acta 2017, 457, 7–18. 10.1016/j.ica.2016.12.002.

[ref24] Ponya Utthra P.; Kumaravel G.; Senthilkumar R.; Raman N. Heteroleptic Schiff Base Complexes Containing Terpyridine as Chemical Nucleases and Their Biological Potential: A Study of DNA Binding and Cleaving, Antimicrobial and Cytotoxic Tendencies. Appl. Organomet. Chem. 2017, 31 (6), e3629 10.1002/aoc.3629.

[ref25] Glišić B. D̵.; Nikodinovic-Runic J.; Ilic-Tomic T.; Wadepohl H.; Veselinović A.; Opsenica I. M.; Djuran M. I. Synthesis, Cytotoxic Activity and DNA-Binding Properties of Copper(II) Complexes with Terpyridine. Polyhedron 2018, 139, 313–322. 10.1016/j.poly.2017.11.008.

[ref26] Erxleben A. Interactions of Copper Complexes with Nucleic Acids. Coord. Chem. Rev. 2018, 360, 92–121. 10.1016/j.ccr.2018.01.008.

[ref27] Jain S.; Bhar K.; Kumar S.; Bandyopadhyaya S.; Tapryal S.; Mandal C. C.; Sharma A. K. Homo- and Heteroleptic Trimethoxy Terpyridine-Cu(Ii) Complexes: Synthesis, Characterization, DNA/BSA Binding, DNA Cleavage and Cytotoxicity Studies. Dalton Trans. 2020, 49 (13), 4100–4113. 10.1039/D0DT00209G. 32141470

[ref28] Maity B.; Gadadhar S.; Goswami T. K.; Karande A. A.; Chakravarty A. R. Impact of Metal on the DNA Photocleavage Activity and Cytotoxicity of Ferrocenyl Terpyridine 3d Metal Complexes. Dalton Trans. 2011, 40 (44), 11904–11913. 10.1039/c1dt11102g. 21975663

[ref29] Manikandamathavan V. M.; Unni Nair B. DNA Binding and Cytotoxicity of Copper (II) Imidazole Terpyridine Complexes: Role of Oxyanion, Hydrogen Bonding and π-π Interaction. Eur. J. Med. Chem. 2013, 68, 244–252. 10.1016/j.ejmech.2013.07.051. 23981531

[ref30] Grau J.; Brissos R. F.; Salinas-Uber J.; Caballero A. B.; Caubet A.; Roubeau O.; Korrodi-Gregório L.; Pérez-Tomás R.; Gamez P. The Effect of Potential Supramolecular-Bond Promoters on the DNA-Interacting Abilities of Copper-Terpyridine Compounds. Dalton Trans. 2015, 44 (36), 16061–16072. 10.1039/C5DT02211H. 26287737

[ref31] Shobha Devi C.; Thulasiram B.; Aerva R. R.; Nagababu P. Recent Advances in Copper Intercalators as Anticancer Agents. J. Fluoresc. 2018, 28 (5), 1195–1205. 10.1007/s10895-018-2283-7. 30171479

[ref32] Shao J.; Li M.; Guo Z.; Jin C.; Zhang F.; Ou C.; Xie Y.; Tan S.; Wang Z.; Zheng S.; Wang X. TPP-Related Mitochondrial Targeting Copper (II) Complex Induces P53-Dependent Apoptosis in Hepatoma Cells through ROS-Mediated Activation of Drp1. Cell Commun. Signal. 2019, 17 (1), 149 10.1186/s12964-019-0468-6. 31744518 PMC6862763

[ref33] Malarz K.; Zych D.; Kuczak M.; Musioł R.; Mrozek-Wilczkiewicz A. Anticancer Activity of 4′-Phenyl-2,2′:6′,2″-Terpyridines - behind the Metal Complexation. Eur. J. Med. Chem. 2020, 189, 112039 10.1016/j.ejmech.2020.112039. 31962262

[ref34] Roy S.; Saha S.; Majumdar R.; Dighe R. R.; Chakravarty A. R. Photo-Activated Cytotoxicity of a Pyrenyl-Terpyridine Copper(II) Complex in HeLa Cells. Polyhedron 2010, 29 (17), 3251–3256. 10.1016/j.poly.2010.09.002.

[ref35] Rajalakshmi S.; Weyhermüller T.; Dinesh M.; Nair B. U. Copper(II) Complexes of Terpyridine Derivatives: A Footstep towards Development of Antiproliferative Agent for Breast Cancer. J. Inorg. Biochem. 2012, 117, 48–59. 10.1016/j.jinorgbio.2012.08.010. 23078774

[ref36] Mahendiran D.; Kumar R. S.; Viswanathan V.; Velmurugan D.; Rahiman A. K. Targeting of DNA Molecules, BSA/c-Met Tyrosine Kinase Receptors and Anti-Proliferative Activity of Bis(Terpyridine)Copper(II) Complexes. Dalton Trans. 2016, 45 (18), 7794–7814. 10.1039/C5DT03831F. 27063595

[ref37] Karges J.; Xiong K.; Blacque O.; Chao H.; Gasser G. Highly Cytotoxic Copper(II) Terpyridine Complexes as Anticancer Drug Candidates. Inorg. Chim. Acta 2021, 516, 120137 10.1016/j.ica.2020.120137.

[ref38] Czerwińska K.; Machura B.; Kula S.; Krompiec S.; Erfurt K.; Roma-Rodrigues C.; Fernandes A. R.; Shul’pina L. S.; Ikonnikov N. S.; Shul’pin G. B. Copper(II) Complexes of Functionalized 2,2′:6′,2″-Terpyridines and 2,6-Di(Thiazol-2-Yl)Pyridine: Structure, Spectroscopy, Cytotoxicity and Catalytic Activity. Dalton Trans. 2017, 46 (29), 9591–9604. 10.1039/C7DT01244F. 28702618

[ref39] Choroba K.; Machura B.; Szlapa-Kula A.; Malecki J. G.; Raposo L.; Roma-Rodrigues C.; Cordeiro S.; Baptista P. V.; Fernandes A. R. Square Planar Au(III), Pt(II) and Cu(II) Complexes with Quinoline-Substituted 2,2′:6′,2″-Terpyridine Ligands: From in Vitro to in Vivo Biological Properties. Eur. J. Med. Chem. 2021, 218, 113404 10.1016/j.ejmech.2021.113404. 33823390

[ref40] Banerjee I.; Samanta P. N.; Das K. K.; Ababei R.; Kalisz M.; Girard A.; Mathonière C.; Nethaji M.; Clérac R.; Ali M. Air Oxygenation Chemistry of 4-TBC Catalyzed by Chloro Bridged Dinuclear Copper(II) Complexes of Pyrazole Based Tridentate Ligands: Synthesis, Structure, Magnetic and Computational Studies. Dalton Trans. 2013, 42 (5), 1879–1892. 10.1039/C2DT30983A. 23172025

[ref41] Spek A. L. PLATON SQUEEZE: A Tool for the Calculation of the Disordered Solvent Contribution to the Calculated Structure Factors. Acta Crystallogr., Sect. C: Struct. Chem. 2015, 71 (1), 9–18. 10.1107/S2053229614024929. 25567569

[ref42] Addison A. W.; Rao T. N.; Reedijk J.; van Rijn J.; Verschoor G. C. Synthesis, Structure, and Spectroscopic Properties of Copper(II) Compounds Containing Nitrogen-Sulphur Donor Ligands; the Crystal and Molecular Structure of Aqua[1,7-Bis(N -Methylbenzimidazol-2′-Yl)-2,6-Dithiaheptane]Copper(II) Perchlorate. J. Chem. Soc., Dalton Trans. 1984, 1349–1356. 10.1039/DT9840001349.

[ref43] Heyns A. M.; van Schalkwyk G. J. A Study of the Infrared and Raman Spectra of Ammonium Hexafluorophosphate NH4PF6 over a Wide Range of Temperatures. Spectrochim. Acta, Part A 1973, 29 (6), 1163–1175. 10.1016/0584-8539(73)80154-0.

[ref44] Ali I.; Wani W. A.; Saleem K. Empirical Formulae to Molecular Structures of Metal Complexes by Molar Conductance. Synth. React. Inorg., Met.-Org., Nano-Met. Chem. 2013, 43 (9), 1162–1170. 10.1080/15533174.2012.756898.

[ref45] Todorović T.; Grubišić S.; Pregelj M.; Jagodič M.; Misirlić-Denčić S.; Dulović M.; Marković I.; Klisurić O.; Malešević A.; Mitić D.; And̵elković K.; Filipović N. Structural, Magnetic, DFT, and Biological Studies of Mononuclear and Dinuclear CuII Complexes with Bidentate N-Heteroaromatic Schiff Base Ligands. Eur. J. Inorg. Chem. 2015, 2015 (23), 3921–3931. 10.1002/ejic.201500349.

[ref46] Stockert J. C.; Horobin R. W.; Colombo L. L.; Blázquez-Castro A. Tetrazolium Salts and Formazan Products in Cell Biology: Viability Assessment, Fluorescence Imaging, and Labeling Perspectives. Acta Histochem. 2018, 120 (3), 159–167. 10.1016/j.acthis.2018.02.005. 29496266

[ref47] Pedrosa P.; Mendes R.; Cabral R.; Martins L. M. D. R. S.; Baptista P. V.; Fernandes A. R. Combination of Chemotherapy and Au-Nanoparticle Photothermy in the Visible Light to Tackle Doxorubicin Resistance in Cancer Cells. Sci. Rep. 2018, 8 (1), 11429 10.1038/s41598-018-29870-0. 30061701 PMC6065399

[ref48] Choroba K.; Filipe B.; Świtlicka A.; Penkala M.; Machura B.; Bieńko A.; Cordeiro S.; Baptista P. V.; Fernandes A. R. In Vitro and In Vivo Biological Activities of Dipicolinate Oxovanadium(IV) Complexes. J. Med. Chem. 2023, 66, 8580–8599. 10.1021/acs.jmedchem.3c00255. 37311060 PMC10350923

[ref49] Maroń A.; Czerwińska K.; Machura B.; Raposo L.; Roma-Rodrigues C.; Fernandes A. R.; Małecki J. G.; Szlapa-Kula A.; Kula S.; Krompiec S. Spectroscopy, Electrochemistry and Antiproliferative Properties of Au(Iii), Pt(Ii) and Cu(Ii) Complexes Bearing Modified 2,2′:6′,2″-Terpyridine Ligands. Dalton Trans. 2018, 47 (18), 6444–6463. 10.1039/C8DT00558C. 29688241

[ref50] Bukowski K.; Kciuk M.; Kontek R. Mechanisms of Multidrug Resistance in Cancer Chemotherapy. Int. J. Mol. Sci. 2020, 21 (9), 3233 10.3390/ijms21093233. 32370233 PMC7247559

[ref51] Ji P.; Wang P.; Chen H.; Xu Y.; Ge J.; Tian Z.; Yan Z. Potential of Copper and Copper Compounds for Anticancer Applications. Pharmaceuticals 2023, 16 (2), 234 10.3390/ph16020234. 37259382 PMC9960329

[ref52] Roma-Rodrigues C.; Pombo I.; Fernandes A. R.; Baptista P. V. Hyperthermia Induced by Gold Nanoparticles and Visible Light Photothermy Combined with Chemotherapy to Tackle Doxorubicin Sensitive and Resistant Colorectal Tumor 3D Spheroids. Int. J. Mol. Sci. 2020, 21 (21), 8017–8113. 10.3390/ijms21218017. 33126535 PMC7672550

[ref53] Valente R.; Cordeiro S.; Luz A.; Melo M. C.; Rodrigues C. R.; Baptista P. V.; Fernandes A. R. Doxorubicin-Sensitive and -Resistant Colorectal Cancer Spheroid Models: Assessing Tumor Microenvironment Features for Therapeutic Modulation. Front. Cell Dev. Biol. 2023, 11, 1310397 10.3389/fcell.2023.1310397. 38188017 PMC10771845

[ref54] Zanoni M.; Piccinini F.; Arienti C.; Zamagni A.; Santi S.; Polico R.; Bevilacqua A.; Tesei A. 3D Tumor Spheroid Models for in Vitro Therapeutic Screening: A Systematic Approach to Enhance the Biological Relevance of Data Obtained. Sci. Rep. 2016, 6 (1), 19103 10.1038/srep19103. 26752500 PMC4707510

[ref55] Sequeira D.; Baptista P. V.; Valente R.; Piedade M. F. M.; Garcia M. H.; Morais T. S.; Fernandes A. R. Cu(i) Complexes as New Antiproliferative Agents against Sensitive and Doxorubicin Resistant Colorectal Cancer Cells: Synthesis, Characterization, and Mechanisms of Action. Dalton Trans. 2021, 50 (5), 1845–1865. 10.1039/D0DT03566A. 33470993

[ref56] Verheijen M.; Lienhard M.; Schrooders Y.; Clayton O.; Nudischer R.; Boerno S.; Timmermann B.; Selevsek N.; Schlapbach R.; Gmuender H.; Gotta S.; Geraedts J.; Herwig R.; Kleinjans J.; Caiment F. DMSO Induces Drastic Changes in Human Cellular Processes and Epigenetic Landscape in Vitro. Sci. Rep. 2019, 9 (1), 4641 10.1038/s41598-019-40660-0. 30874586 PMC6420634

[ref57] Sharma B.; Kanwar S. S. Phosphatidylserine: A Cancer Cell Targeting Biomarker. Semin. Cancer Biol. 2018, 52, 17–25. 10.1016/j.semcancer.2017.08.012. 28870843

[ref58] Kumar R.; Saneja A.; Panda A. K. An Annexin V-FITC-Propidium Iodide-Based Method for Detecting Apoptosis in a Non-Small Cell Lung Cancer Cell Line. Methods Mol. Biol. 2021, 2279, 213–223. 10.1007/978-1-0716-1278-1_17. 33683697

[ref59] Mahendiran D.; Amuthakala S.; Bhuvanesh N. S. P.; Kumar R. S.; Rahiman A. K. Copper Complexes as Prospective Anticancer Agents: In Vitro and in Vivo Evaluation, Selective Targeting of Cancer Cells by DNA Damage and S Phase Arrest. RSC Adv. 2018, 8 (30), 16973–16990. 10.1039/C8RA00954F. 35540520 PMC9080330

[ref60] Meredith A. M.; Dass C. R. Increasing Role of the Cancer Chemotherapeutic Doxorubicin in Cellular Metabolism. J. Pharm. Pharmacol. 2016, 68 (6), 729–741. 10.1111/jphp.12539. 26989862

[ref61] Galluzzi L.; López-Soto A.; Kumar S.; Kroemer G. Caspases Connect Cell-Death Signaling to Organismal Homeostasis. Immunity 2016, 44 (2), 221–231. 10.1016/j.immuni.2016.01.020. 26885855

[ref62] Strasser A.; Vaux D. L. Cell Death in the Origin and Treatment of Cancer. Mol. Cell 2020, 78 (6), 1045–1054. 10.1016/j.molcel.2020.05.014. 32516599

[ref63] Pecorino L. Molecular Biology of Cancer; Oxford University Press, 2012.

[ref64] Yu L.; Chen Y.; Tooze S. A. Autophagy Pathway: Cellular and Molecular Mechanisms. Autophagy 2018, 14 (2), 207–215. 10.1080/15548627.2017.1378838. 28933638 PMC5902171

[ref65] Jung S.; Jeong H.; Yu S.-W. Autophagy as a Decisive Process for Cell Death. Exp. Mol. Med. 2020, 52 (6), 921–930. 10.1038/s12276-020-0455-4. 32591647 PMC7338414

[ref66] Polloni L.; Seni Silva A. C. D.; Teixeira S. C.; Azevedo F. V. P. D. V.; Zóia M. A. P.; Da Silva M. S.; Lima P. M. A. P.; Correia L. I. V.; Do Couto Almeida J.; Da Silva C. V.; Rodrigues Ávila V. d. M.; Goulart L. R. F.; Morelli S.; Guerra W.; Oliveira Júnior R. J. d. Action of copper(II) complex with β-diketone and 1,10-phenanthroline (CBP-01) on sarcoma cells and biological effects under cell death. Biomed. Pharmacother. 2019, 112, 108586 10.1016/j.biopha.2019.01.047. 30784909

[ref67] Maadi H.; Soheilifar M. H.; Wang Z. Analysis of Cell Cycle by Flow Cytometry. Methods Mol. Biol. 2022, 2579, 183–195. 10.1007/978-1-0716-2736-5_14. 36045207

[ref68] Zehra S.; Roisnel T.; Arjmand F. Enantiomeric Amino Acid Schiff Base Copper(II) Complexes as a New Class of RNA-Targeted Metallo-Intercalators: Single X-Ray Crystal Structural Details, Comparative in Vitro DNA/RNA Binding Profile, Cleavage, and Cytotoxicity. ACS Omega 2019, 4 (4), 7691–7705. 10.1021/acsomega.9b00131.

[ref69] Machado P. H. A.; Paixão D. A.; Lino R. C.; De Souza T. R.; De Souza Bontempo N. J.; Sousa L. M.; Van Petten De Vasconcelos Azevedo F.; Orsolin P. C.; Lima P. M. A. P.; Martins I. C.; Da Costa Guerra J. F.; Teixeira S. C.; Araújo T. G.; Goulart L. R.; Morelli S.; Guerra W.; de Oliveira Júnior R. J. A Selective CuII Complex with 4-Fluorophenoxyacetic Acid Hydrazide and Phenanthroline Displays DNA-Cleaving and pro-Apoptotic Properties in Cancer Cells. Sci. Rep. 2021, 11 (1), 24450 10.1038/s41598-021-03909-1. 34961767 PMC8712526

[ref70] Pitie M.; Burrows C. J.; Meunier B. Mechanisms of DNA Cleavage by Copper Complexes of 3-Clip-Phen and of Its Conjugate with a Distamycin Analogue. Nucleic Acids Res. 2000, 28 (24), 4856–4864. 10.1093/nar/28.24.4856. 11121476 PMC115237

[ref71] Deweese J. E.; Osheroff N. The DNA Cleavage Reaction of Topoisomerase II: Wolf in Sheep’s Clothing. Nucleic Acids Res. 2009, 37 (3), 738–748. 10.1093/nar/gkn937. 19042970 PMC2647315

[ref72] Kim H. S.; Lee Y. S.; Kim D. K. Doxorubicin Exerts Cytotoxic Effects through Cell Cycle Arrest and Fas-Mediated Cell Death. Pharmacology 2009, 84 (5), 300–309. 10.1159/000245937. 19829019

[ref73] Lenis-Rojas O. A.; Roma-Rodrigues C.; Carvalho B.; Cabezas-Sainz P.; Fernández Vila S.; Sánchez L.; Baptista P. V.; Fernandes A. R.; Royo B. In Vitro and In Vivo Biological Activity of Ruthenium 1,10-Phenanthroline-5,6-Dione Arene Complexes. Int. J. Mol. Sci. 2022, 23 (21), 13594 10.3390/ijms232113594. 36362381 PMC9656482

[ref74] Kumari R.; Jat P. Mechanisms of Cellular Senescence: Cell Cycle Arrest and Senescence Associated Secretory Phenotype. Front. Cell Dev. Biol. 2021, 9, 645593 10.3389/fcell.2021.645593. 33855023 PMC8039141

[ref75] Liu J.; He Y.; Liu D.; He Y.; Tang Z.; Lou H.; Huo Y.; Cao X. Characterizing the Binding Interaction of Astilbin with Bovine Serum Albumin: A Spectroscopic Study in Combination with Molecular Docking Technology. RSC Adv. 2018, 8 (13), 7280–7286. 10.1039/C7RA13272G. 35540350 PMC9078437

[ref76] Jahanban-Esfahlan A.; Panahi-Azar V.; Sajedi S. Spectroscopic and Molecular Docking Studies on the Interaction between *N* -acetyl Cysteine and Bovine Serum Albumin. Biopolymers 2015, 103 (11), 638–645. 10.1002/bip.22697. 26139573

[ref77] İnci D.; Aydın R.; Vatan O. ¨.; Zorlu Y.; Çinkılıç N. New Binary Copper(II) Complexes Containing Intercalating Ligands: DNA Interactions, an Unusual Static Quenching Mechanism of BSA and Cytotoxic Activities. J. Biomol. Struct. Dyn. 2018, 36 (15), 3878–3901. 10.1080/07391102.2017.1404936. 29132253

[ref78] Acharya P.; Maity R.; Kuila A.; Maity T.; Maity S.; Sepay N.; Samanta B. C. Hydrophobicity-Induced DNA, BSA Binding, and Biomaterial Applications of a Heteroleptic Cu(II) Complex. Appl. Organomet. Chem. 2022, 36 (5), e6640 10.1002/aoc.6640.

[ref79] Sirajuddin M.; Ali S.; Badshah A. Drug-DNA Interactions and Their Study by UV-Visible, Fluorescence Spectroscopies and Cyclic Voltametry. J. Photochem. Photobiol., B 2013, 124, 1–19. 10.1016/j.jphotobiol.2013.03.013. 23648795

[ref80] Rehman S. U.; Sarwar T.; Husain M. A.; Ishqi H. M.; Tabish M. Studying Non-Covalent Drug-DNA Interactions. Arch. Biochem. Biophys. 2015, 576, 49–60. 10.1016/j.abb.2015.03.024. 25951786

[ref81] Wolfe A.; Shimer G. H.; Meehan T. Polycyclic Aromatic Hydrocarbons Physically Intercalate into Duplex Regions of Denatured DNA. Biochemistry 1987, 26 (20), 6392–6396. 10.1021/bi00394a013. 3427013

[ref82] Lakowicz J. R.; Weber G. Quenching of Fluorescence by Oxygen. Probe for Structural Fluctuations in Macromolecules. Biochemistry 1973, 12 (21), 4161–4170. 10.1021/bi00745a020. 4795686 PMC6959846

[ref83] Lakowicz J. R. Principles of Fluorescence Spectroscopy, 3rd ed.; Springer US, 2006.

[ref84] Anderson R. L.; Balasas T.; Callaghan J.; Coombes R. C.; Evans J.; Hall J. A.; Kinrade S.; Jones D.; Jones P. S.; Jones R.; Marshall J. F.; Panico M. B.; Shaw J. A.; Steeg P. S.; Sullivan M.; Tong W.; Westwell A. D.; Ritchie J. W. A. A Framework for the Development of Effective Anti-Metastatic Agents. Nat. Rev. Clin. Oncol. 2019, 16 (3), 185–204. 10.1038/s41571-018-0134-8. 30514977 PMC7136167

[ref85] Gomes R. N.; Manuel F.; Nascimento D. S. The Bright Side of Fibroblasts: Molecular Signature and Regenerative Cues in Major Organs. npj Regener. Med. 2021, 6 (1), 43 10.1038/s41536-021-00153-z. PMC8355260 34376677

[ref86] Balsa L. M.; Ruiz M. C.; Santa Maria de la Parra L.; Baran E. J.; León I. E. Anticancer and Antimetastatic Activity of Copper(II)-Tropolone Complex against Human Breast Cancer Cells, Breast Multicellular Spheroids and Mammospheres. J. Inorg. Biochem. 2020, 204, 110975 10.1016/j.jinorgbio.2019.110975. 31911364

[ref87] Asghariazar V.; Amini M.; Pirdel Z.; Fekri R.; Asadi A.; Nejati-Koshki K.; Baradaran B.; Panahi Y. The Schiff Base Hydrazine Copper(II) Complexes Induce Apoptosis by P53 Overexpression and Prevent Cell Migration through Protease-Independent Pathways. Med. Oncol. 2023, 40 (9), 271 10.1007/s12032-023-02150-2. 37594547

[ref88] Castaneda M.; den Hollander P.; Kuburich N. A.; Rosen J. M.; Mani S. A. Mechanisms of Cancer Metastasis. Semin. Cancer Biol. 2022, 87, 17–31. 10.1016/j.semcancer.2022.10.006. 36354098

[ref89] Naik M.; Brahma P.; Dixit M. A Cost-Effective and Efficient Chick Ex-Ovo Cam Assay Protocol to Assess Angiogenesis. Methods Protoc. 2018, 1 (2), 19 10.3390/mps1020019. 31164562 PMC6526448

[ref90] Fraguas-Sánchez A. I.; Martín-Sabroso C.; Torres-Suárez A. I. The Chick Embryo Chorioallantoic Membrane Model: A ResearchApproach for Ex Vivo and In Vivo Experiments. Curr. Med. Chem. 2022, 29 (10), 1702–1717. 10.2174/0929867328666210625105438. 34176455

[ref91] Maroń A.; Szlapa A.; Klemens T.; Kula S.; Machura B.; Krompiec S.; Małecki J. G.; Świtlicka-Olszewska A.; Erfurt K.; Chrobok A. Tuning the Photophysical Properties of 4′-Substituted Terpyridines - an Experimental and Theoretical Study. Org. Biomol. Chem. 2016, 14 (15), 3793–3808. 10.1039/C6OB00038J. 27005327

[ref92] Choroba K.; Machura B.; Kula S.; Raposo L. R.; Fernandes A. R.; Kruszynski R.; Erfurt K.; Shul’pina L. S.; Kozlov Y. N.; Shul’pin G. B. Copper(II) Complexes with 2,2′:6′,2″-Terpyridine, 2,6-Di(Thiazol-2-Yl)Pyridine and 2,6-Di(Pyrazin-2-Yl)Pyridine Substituted with Quinolines. Synthesis, Structure, Antiproliferative Activity, and Catalytic Activity in the Oxidation of Alkanes and Alcohols with Peroxides. Dalton Trans. 2019, 48 (33), 12656–12673. 10.1039/C9DT01922G. 31384866

[ref93] Małecka M.; Machura B.; Świtlicka A.; Kotowicz S.; Szafraniec-Gorol G.; Siwy M.; Szalkowski M.; Maćkowski S.; Schab-Balcerzak E. Towards Better Understanding of Photophysical Properties of Rhenium(I) Tricarbonyl Complexes with Terpy-like Ligands. Spectrochim. Acta, Part A 2020, 231, 118124 10.1016/j.saa.2020.118124. 32062513

[ref94] Szlapa-Kula A.; Małecka M.; Machura B. Insight into Structure-Property Relationships of Aryl-Substituted 2,2′:6′,2″-Terpyridines. Dyes Pigments 2020, 180, 108480 10.1016/j.dyepig.2020.108480.

[ref95] Choroba K.; Maroń A.; Świtlicka A.; Szłapa-Kula A.; Siwy M.; Grzelak J.; Maćkowski S.; Pedzinski T.; Schab-Balcerzak E.; Machura B. Carbazole Effect on Ground- and Excited-State Properties of Rhenium(I) Carbonyl Complexes with Extended Terpy-like Ligands. Dalton Trans. 2021, 50 (11), 3943–3958. 10.1039/D0DT04340K. 33645614

[ref96] Cooke M. W.; Wang J.; Theobald I.; Hanan G. S. Convenient One-Pot Procedures for the Synthesis of 2,2′:6′,2″-Terpyridine. Synth. Commun. 2006, 36 (12), 1721–1726. 10.1080/00397910600616750.

[ref97] Toledo D.; Brovelli F.; Soto-Delgado J.; Peña O.; Pivan J.-Y.; Moreno Y. Influence of Structural Changes on Photophysical Properties of Terpyridine Derivates: Experimental Studies and Theoretical Calculations. J. Mol. Struct. 2018, 1153, 282–291. 10.1016/j.molstruc.2017.10.011.

