# Supplementary material for: Copper(II) Complexes with 2,2′:6′,2″-Terpyridine Derivatives Displaying Dimeric Dichloro−μ–Bridged Crystal Structure: Biological Activities from 2D and 3D Tumor Spheroids to In Vivo Models
Source: J Med Chem. 2024 Mar 22;67(7):5813–36. doi: 10.1021/acs.jmedchem.4c00119 (PMC11017252; doi:10.1021/acs.jmedchem.4c00119)
Supplement: Supplementary file 1 — jm4c00119_si_001.pdf [file jm4c00119_si_001.pdf]

SUPPLEMENTARY INFORMATION

Copper(II) complexes with 2,2′:6′,2″-terpyridine derivatives displaying dimeric dichloro–µ–bridged crystal structure: biological activities from 2D and 3D tumor spheroids to *in vivo* models

Katarzyna Choroba[a*], Barbara Machura[a], Karol Erfurt[b], Ana Rita Casimiro[c,d], Sandra Cordeiro[c,d], Pedro V. Baptista[c,d], Alexandra R. Fernandes[c,d*]

[a] *Institute of Chemistry, University of Silesia, Szkolna 9, 40-006 Katowice, Poland.*
[b] *Department of Chemical Organic Technology and Petrochemistry, Silesian University of Technology, Krzywoustego 4, 44-100 Gliwice, Poland*
[c] *Associate Laboratory i4HB - Institute for Health and Bioeconomy, NOVA School of Science and Technology, NOVA University Lisbon, 2819-516 Caparica, Portugal*
[d] *UCIBIO, Departamento de Ciências da Vida, NOVA School of Science and Technology, Campus de Caparica, 2829-516 Caparica, Portugal.*

*Corresponding authors: katarzyna.choroba@us.edu.pl (KC) and ma.fernandes@fct.unl.pt (ARF)

Table of Contents

Experimental section		page S2-S9
HR-ESI-MS spectra of the complexes **1-8**	**Figure S1**	page S10-S16
UPLC of the complexes **1-8**	**Figure S2**	page S16-S19
Crystal data and structure refinement	**Table S1**	page S20
Dimer units of compounds **1, 3, 4, 6, 7** and **8**	**Figure S3**	page S21-S22
The selected structural data of compounds **1-8**	**Table S2**	page S23
Selected bond lengths [Å] and angles [°] of compounds **1-8**	**Table S3**	page S23
Short intra- and intermolecular hydrogen bonds detected in complexes **1-8**	**Table S4**	page S24
Short π•••π interactions for complexes **1-8**	**Table S5**	page S25
X–Y•••Cg(J)(π-ring) interactions for complexes **1-8**	**Table S6**	page S26
FT-IR spectra of the complexes **1-8**	**Figure S4**	page S27-S30
Molar conductivity values of the complexes in DMSO in 25 °C	**Table S7**	page S31
Plot of the molar conductivities vs time of the representative samples of **1, 3, 5** and **7.**	**Figure S5**	page S31
UV-Vis properties of Cu(II) compounds in DMSO and in solid samples	**Table S8**	page S32
UV-Vis spectra of complexes **1**–**8** in DMSO	**Figure S6**	page S32
Comparison of the d–d range of the UV-Vis spectra of **1-8** in DMSO and their reflectance solid spectra	**Figure S7**	page S33
UV-Vis spectra of compounds **1-8** and respective ligands **L1-L8** in DMSO	**Figure S8**	page S34
Spectra of compounds **1-8** in DMSO by UV-Visible spectroscopy over 48 h	**Figure S9**	page S35
Spectra of compounds **1-8** in PBS by UV-Visible spectroscopy over 48 h	**Figure S10**	page S36
Cell viability of HCT116, HCT116DoxR, and A2780 tumor cell lines and primary normal fibroblasts after exposure to different concentrations of copper complexes **1 (A)**, **4 (B)**, **6 (C)**, and **8 (D)** for 48 h	**Figure S11**	page S37
Viability of HCT116 cancer cell line after exposure to different concentrations of doxorubicin **(A)** and cisplatin **(B)** for 48 h. Viability of A2780 cancer cell line after exposure to different concentrations of doxorubicin **(C)** and cisplatin **(D)** for 48 h	**Figure S12**	page S38
Cell viability of HCT116DoxR tumor cell line after exposure to different concentrations of ligands **L1-L8** for 48 h. DMSO in the same % as in the ligands was used as the vehicle control	**Figure S13**	page S39
Cell viability of fibroblasts after exposure to different concentrations of ligands **L1-L8** for 48 h. DMSO in the same % as in the ligands was used as	**Figure S14**	page S40

the vehicle control		
Fluorescence microscopy of HCT116DoxR cells incubated for 3 h with 10x the IC_{50} of complex **2**	**Figure S15**	page S41
Fluorescence microscopy of HCT116DoxR cells incubated for 3 h with 10x the IC_{50} of complex **3**	**Figure S16**	page S41
Confocal microscopy of HCT116DoxR cells incubated for 3 h with 10x the IC_{50} concentrations of complexes **2 (A)** and **3 (B)**	**Figure S17**	page S42
Evaluation of the stability and solubility of complexes **2 (A)**, **3 (B)**, **5 (C)**, and **7 (D)** by UV-Visible spectroscopy over 48 h	**Figure S18**	page S42
Western blot bands used to quantify BAX and BCL-2 proteins in HCT116DoxR cells after 48 h exposure to the complexes **2**, **3**, **5**, and **7** or 0.1% (v/v) DMSO	**Figure S19**	page S43
Electrophoresis in 0.8% (w/v) agarose gel to analyse the electrophoretic mobility of the pDNA samples after 24 h of exposure to increasing concentrations of complexes **2**, **3**, **5**, and **7**	**Figure S20**	page S44
Electrophoresis in 1% (w/v) agarose gel to determine the mechanisms of pDNA cleavage by complexes **2, 3, 5,** and **7**	**Figure S21**	page S45
References		page S46

Experimental section

Instrumentation

The IR spectra were performed with use of the KBr pellets technique on a Nicolet iS5 spectrophotometer (Thermo Fischer Scientific, Waltham, MA, USA) in the range 4000-400 cm^{-1}.

Elemental analyses of C, H and N were recorded on a Vario EL Cube analyser (Elementar Analysensysteme GmbH, Langenselbold, Germany).

HRMS analyses were recorded on a Xevo G2 Q-TOF mass spectrometer (Waters Corporation, Milford, MA, USA) with an ESI ion source in positive ion mode. Full-scan MS data were collected from 100 to 1000 Da and scan time 0.5 s. The collection of data was performed in centroid mode, with correction of mass using external reference (Lock-Spray™ leucine enkephalin solution with reference ion $[M+H]^+$ at m/z 556.2771 Da). The obtained data was analysed with the MassLynx software (Waters) incorporated with the instrument.

UPLC analysis was performed with a Waters Acquity UPLC system (Waters Corporation, Milford, USA), equipped with autosampler and diode-array detector (PDA). Chromatographic separations were performed on a Waters Acquity UPLC™ BEH C8 column (2.1 mm x 100mm, 1.7µm particle size). The eluted compounds were monitored in the range of 210-400 nm. The column temperature was maintained at 30°C. The mobile phase consisted of acetonitrile–water (80:20, v/v). The flow rate was 0.30 ml/min, the injection volume was 2 µL and the duration of the run was 3 min.

Single X-ray diffraction measurements were performed at room temperature using Gemini A Ultra diffractometer (Oxford Diffraction Ltd, Abingdon, United Kingdom) with CCD Atlas detector and graphite monochromated MoKα radiation (λ = 0.71073 Å). CrysAlisPro software was used for data collection, cell refinement and data reduction. The structures were solved and refined with Olex2 1.5 employing SHELXS-2014 and SHELXL-2014[1–3] package using Least Squares minimisation. The non-hydrogen atoms were refined anisotropically while hydrogen atoms were calculated and refined using the riding-model idealized positions, with fixed isotropic displacement parameters (for aromatic hydrogens: Uiso(H) = 1.2 Ueq(C), d(C–H) = 0.93 Å; for methyl and ethyl groups: Uiso(H) = 1.5 Ueq(C), d(C–H) = 0.96 Å). The methyl groups were allowed to rotate around its local threefold axis. The structures are deposited at CCDC numbers: 2295797-2295801 (**1–5**), 2296785 (**6**), 2295802 and 2295803 (**7**, **8**) and can be obtained free of charge from The Cambridge Crystallographic Data Center at www.ccdc.cam.ac.uk/getstructures.

The electrical conductivities, κ (S cm^{-1}) of all complexes were measured with a CX-505 multifunction meter (ELMETRON, Zabrze, Poland) equipped with EC-60 electrode. Freshly prepared DMSO solutions (c = 1 mM) were measured at 25.0 ± 0.1 °C. The molar conductivities Λ_M (S cm^2 mol^{-1}) of the complexes were calculated according to the equation: $\Lambda_M = \frac{\kappa}{c} \cdot 10^3$.

The UV-Vis spectroscopic measurements were performed on Nicolet Evolution 220 (Thermo Fischer Scientific, Waltham, MA, USA) spectrophotometer in the range 220–1000 nm. The spectra of compounds were obtained in powdered solid, DMSO and Dulbecco's Modified Phosphate Buffered Saline (PBS, pH 7.4, 130 mM NaCl, Sigma Aldrich) in 10 μM solutions. The stability of the compounds was checked by measuring the spectra of the sample (50 μM) once every four hours for 48 h at room temperature.

Biological assays

Cell culture and maintenance

The cancer cell lines HCT116 (colorectal carcinoma) and A2780 (ovarian carcinoma) were obtained from the American Type Culture Collection (ATCC®, Manassas, VA, USA), as well as the normal human primary dermal fibroblasts. The HCT116 Dox-resistant cell line (HCT116DoxR) was derived from HCT116 parental cells through exposure to increasing concentrations of Dox, as described in Pedrosa, P., et al., (2018)[4].

Apart from the A2780 cell line, which was cultured in Roswell Park Memorial Institute (RPMI) medium, all cells were cultured in Dulbecco’s modified Eagle medium (DMEM). Both media were supplemented with 10% (v/v) fetal bovine serum (FBS) and 1% (v/v) of a Pen/Strep (Penicillin/Streptomycin) solution. To maintain the resistant phenotype of the HCT116DoxR cell line, the medium was additionally supplemented with 3.6 μM Dox[4]. All media and supplements were purchased from Thermo Fischer Scientific (Waltham, MA, USA).

Cells were maintained in 25 cm^2 T-flasks (T25) in a humidified atmosphere at 37ºC and 5% (v/v) CO_2 (SANYO CO_2 Incubator, Electric Biomedical Co., Osaka, Japan).

Cell viability assays

The effect of Cu(II) complexes on cell viability was studied in HCT116, HCT116DoxR, and A2780 cancer cell lines and in normal human primary fibroblasts. Cells were seeded in a density of 0.75×10^5 cells/mL into 96-well plates and incubated in a humidified atmosphere at 37ºC and 5% (v/v) CO_2 for 24 h. After incubation, complexes were weighed, immediately solubilized in 100% (v/v) DMSO and diluted for the final concentration with cell culture medium. The medium in each well was replaced by a solution of fresh medium containing each Cu(II) complex (concentrations ranging from 0.01 μM to 50 μM). Culture medium with DMSO (in the same % as in the complexes) and 0.4 μM Dox were used as negative and positive controls, respectively.

Following a second incubation period of 48 h in the same conditions, the supernatant of each well was replaced by a solution containing MTS reagent (3-(4,5-dimethylthiazol-2-yl)-5-(3-carboxymethoxyphenyl)-2-(4-sulfophenyl)-2H-tetrazolium) provided by the CellTiter 96® AQueous Non-Radioactive Cell Proliferation Assay Kit (Promega, Madison, WI, EUA) for a 30 minutes incubation. The percentage of viable cells was indirectly determined by the spectrophotometric quantification at 490 nm in a microplate reader, Tecan Infinite M200 (Tecan, Männedorf, Switzerland) of formazan production, resultant from the reduction of the MTS reagent by mitochondrial dehydrogenases. Since these enzymes are only active in viable cells, the absorbance value measured is directly proportional to the percentage of viable cells in culture[5].

By analysing the cell viability as a function of $\log_{10}$(concentration) using Prism 8 (GraphPad software), it is possible to determine the IC_{50} (complex concentration which induces a 50% reduction in cell viability) of each complex in the respective cell line at 48 h. The selective index (SI) for each

complex was also determined by dividing the IC_{50} of human fibroblasts by the respective IC_{50} in each cancer cell line (allowing us to understand how much active a complex is in tumor versus normal cell).

3D-spheroid formation and cell viability assays

HCT116DoxR cells were seeded at 5×10^4 cells/mL in super low Attachment 96-well plates (Nunclon™ Sphera™ U-Shaped-Bottom Microplate, Thermo Fisher Scientific, Waltham, MA, USA) and incubated at 37°C, in a humidified atmosphere with 5% (v/v) CO_2, for 6 days to allow spheroids' formation and growth. Following this incubation period, the culture medium was replaced by medium with copper complexes at pretended concentrations. Spheroids were then incubated for 48 h at the same conditions (as in 2D cells) and later the medium was replaced by a solution containing the MTS reagent and DMEM medium (20:100). Spheroids were incubated for 4 h with MTS before being transferred into a 96-well plate with a flat bottom to be analysed in the microplate reader Tecan Infinite M200 (Tecan, Männedorf, Switzerland). This increase incubation period with MTS reagent compared to 2D cells is due to their higher density, complexity and cell number[6] as recommended by the manufacturer.

Cellular internalization

Fluorescence and Confocal microscopy

HCT116DoxR cells were seeded on coverslips in confocal disks at a density of 0.75×10^5 cells/mL and grown for 24 h at the conditions mentioned before and later incubated with complexes **2** or **3** at 10x their IC_{50} concentration or 0.1% (v/v) DMSO (vehicle control). Following 3 h or 6 h of incubation, cells were fixed in 4% paraformaldehyde for 20 min at room temperature (rt), permeabilized with 0.1% (v/v) Triton X-100 (in filtered PBS 1x) for 15 min and incubated with RNAse 50 µg/mL (in filtered PBS 1x) for 30 min at 37ºC. Cells were then washed 3x with filtered PBS 1x and incubated with 0.25 µg/mL Propidium Iodide (PI) (maximum excitation 535 nm and maximum emission 615 nm) for 10 min at 37ºC.

In the case of confocal microscopy, cells were then blocked at rt with 1% BSA for 30 min. Cells were then incubated with Alexa Fluor™ 488-Phalloidin (Thermo Fischer Scientific, Waltham, MA, USA) (maximum excitation at 495 nm and emission maximum at 518 nm) in BSA for 20 min and transferred to a microscopic slide containing a drop of ProLong™ Glass antifade mounting. The slide was stored at 4ºC for 24 h, after which Z-stack images were taken on a Zeiss LSM 710 confocal laser point-scanning fluorescence microscope.

On the other hand, in the case of fluorescence microscopy, after incubation with PI, the images were taken on a Ti-U Eclipse inverted microscope (Nikon, Japan).

The complexes present a maximum excitation at 290 nm in the UV region and their maximum emission at 410-420 nm in the blue region.

ICP-AES (Inductively coupled plasma-atomic emission spectrometry) and Cell Fractionation

Internalization and subcellular localization of copper complexes in HCT116DoxR cell line were assessed by ICP-AES. Cytosolic, mitochondrial, and nuclear cell fractions were obtained using the Abcam Standard (ab109719) Cell Fractionation Kit.

HCT116DoxR cells were seeded in 24-well plates in a density of 2×10^5 cells/mL and incubated for 24 h at the same conditions mentioned before. Afterwards, the culture medium was replaced by fresh medium containing different copper complexes at a concentration equivalent to 20x their IC_{50} concentrations or 0.1% (v/v) DMSO. After 6 h of incubation in the conditions previously described, the culture medium was recovered, and cells were washed with PBS 1x which was also recovered. Cells were detached from each well with TrypLE™ Express (Thermo Fisher Scientific, Waltham, MA, USA) and centrifuged at 500 xg for 5 min (Sigma 1-14, Germany). The resulting supernatant was recovered, and the pellet resuspended in buffer A to a cell density of 6.6×10^6 cells/mL. The pretended cell fractions were later obtained by a series of centrifugations in specific buffers provided by the kit following the manufacturers' instructions. To better characterize subcellular localization of complex **2**,

the previously described assay was repeated, but using the Cell Signaling Cell Fractionation Kit (#9038) to obtain cytosolic, membrane and organelles, and nuclear and cytoskeletal fractions.
The same procedure was followed until the first centrifugation, after which the supernatant was recovered, and the pellet resuspended in cold PBS 1x. The cell concentration was determined, the cells were centrifuged for 5 min at 500 xg and, after recovering the supernatant, the pellet was resuspended in CIB buffer according to the manufacturers' instructions. The desired cell fractions were then obtained by adding the buffers supplied by the kit and subsequent centrifugations, according to the protocol.
Fresh aqua regia (3:1 HCl/HNO_3) was added to all supernatants and all cellular fractions, being left to react overnight at rt in a hood fume. Samples were then delivered to Laboratório de Análises/LAQV and the levels of copper in each sample were evaluated by ICP-AES.

Evaluation of apoptosis induction in HCT116DoxR cell line by flow cytometry

The induction of apoptosis in HCT116DoxR cell line was evaluated by flow cytometry, using the Annexin V-FITC Apoptosis Detection Kit (Thermo Fisher Scientific, Waltham, MA, USA). Cells were seeded in 6-well plates at a cell density of 1×10^5 cells/mL and incubated for 24 h. Afterwards, culture medium was replaced by medium with different Cu(II) complexes at their IC_{50} concentrations. Culture medium with 0.1% (v/v) DMSO, 6 μM Dox or 5 μM Cisplatin (Cis) were used as controls. After 48 h of incubation, cells were washed with PBS 1x, detached from each well with TrypLE™ Express and washed again with PBS 1x. Cells were then resuspended in 1x annexin-binding buffer and incubated at rt for 15 min with Annexin V-FITC and 100 μg/mL PI.
All samples were analysed by an Attune® Acoustic Focusing Flow Cytometer (Thermo Fischer Scientific, Waltham, MA, USA) and the results were processed with Attune® Cytometric software (Biolab@UCIBIO-FCT-NOVA).

Evaluation of BAX and BCL-2 protein expression by Western Blot

HCT116DoxR cell line was cultivated in T25 flasks at a cell density of 2×10^6 cells/mL and incubated for 24 h in the conditions previously described. Afterwards, the culture media was replaced by fresh medium containing IC_{50} concentrations of different Cu(II) complexes or 0.1% (v/v) DMSO (negative control). Following a 48 h incubation period, cells were washed and collected using cold PBS 1x and a cell scraper. The samples were centrifuged for 5 min at 700 xg and resuspended in fresh lysis buffer (150 mM NaCl; 5 mM ethylenediaminetetraacetic acid (EDTA); 50 mM Tris-HCl pH 8.0; 1x phosphatase inhibitors (PhosStop, Roche); 1x protease inhibitors (complete ULTRA tablets, Mini, EASYpack, Roche, Switzerland); 2% (v/v) NP-40 (Thermo Fisher Scientific, Waltham, MA, USA); 1 mM phenylmethylsulfonyl fluoride (PMSF, Merck, Germany) and 0.1% 1,4-dithiothreitol (v/v) (DTT, Merck, Germany).
Each sample was then submitted to 5 ultrasound cycles on ice (2 min and 30s on ultrasounds followed by a 1 min period on ice; UTR200; Hielscher Ultrasonics, Germany) and later centrifuged at 1000 xg for 5 min. After protein extract (from supernatant) quantification with Pierce 660 nm Protein Assay Reagent (Thermo Fisher Scientific, Waltham, MA, USA), 20 μg of protein were loaded on 10% (v/v) polyacrylamide gel and transferred to a PVDF membrane (GE Healthcare Life Sciences, Germany). Each membrane was then incubated for 1 h at rt and constant agitation with 5% (w/v) non-fat milk solubilized in TBST 1x buffer (50 mM Tris-HCl, pH 7.5, 150 mM NaCl, 0.1% (v/v) Tween-20) and later incubated in the same conditions with a solution containing the respective primary antibody in 5% non-fat milk in TBST 1x, specifically anti-BAX (1:5000; Abcam, United Kingdom) and anti BCL-2 (1:1000; Sigma, St. Louis, USA).
Afterwards, membranes were submitted to three wash cycles of 5 min with TBST 1x buffer at rt and constant agitation. This procedure was repeated in the incubation with the secondary antibody (1:3000, Anti-mouse IgG, horseradish peroxidase (HPR)-linked Antibody or 1:2000, Anti-rabbit IgG, HPR-linked Antibody; Cell Signalling Technology, USA). To reveal the protein bands, membranes were

treated with ECL^{TM} Prime Western Blotting Detection Reagents (Cytiva, United Kingdom) for 5 min and exposed to a film in a dark room. Membranes were later incubated (with agitation) twice with Stripping buffer (0.1M glycine, 20 mM magnesium acetate, 50 mM KCl, pH 2.0) during 10 and 20 min, respectively, and subsequently incubated with anti-β-actin (1:5000; Sigma, St. Louis, USA) as a control to normalize the results. Proteins were quantified by densitometry using Image J software.

Caspase-8 activity

The Caspase-8 activity in HCT116DoxR cells exposed to Cu(II) complexes was evaluated using the Caspase-8 Assay kit (ab39700) (Abcam, Cambridge, United Kingdom). Cells were cultivated in T25 flasks at a cell density of 2×10^6 cells/mL and incubated for 24 h at the conditions mentioned before. Afterwards, medium was replaced with fresh medium supplemented with 0.1% (v/v) DMSO, 5 μM Cis or the IC_{50} concentrations of each copper complex and cells were incubated for 48 h. Then, cells were washed and collected using cold PBS 1x and a cell scraper into eppendorfs. After a 5 min centrifugation at 500 xg and 4ºC, cells were washed with cold PBS 1x and centrifuged at 800 xg and 4ºC for 5 min. The resulting pellet was then resuspended in 50 μL of cold Lysis Buffer and the cells were incubated on ice for 20 min, being then centrifuged at 10 000 x g and 4ºC for 10 min. 200 μg of protein were transferred to a 96-well plate in duplicate at a final volume of 50 μL of sample. Afterwards, freshly prepared Reaction mix 1x was added to each well, as well as 5 μL of IETD-pNA. The plate was then incubated for 2 h at 37°C, after which, absorbance was read at 400 nm on a Tecan Infinite M200 microplate reader (Männedorf, Switzerland).

Evaluation of mitochondrial membrane potential ($\Delta\Psi_m$) in HCT116DoxR cell line by flow cytometry

$\Delta\Psi_m$ was evaluated in HCT116DoxR cell line by using the JC-1 Mitochondrial Membrane Potential Assay Kit (Abnova Corporation, Walnut, CA, USA). Cells were seeded in 6-well plates at a density of 1×10^5 cells/mL and, after 24 h of incubation, culture medium was replaced by medium with IC_{50} concentrations of different Cu(II) complexes. 0.1% (v/v) DMSO was used as a negative (vehicle) control while 6 μM Dox and 5 μM Cis were used as positive controls. Following an incubation period of 48 h, cells were washed with PBS 1x, detached with TrypLE^{TM} Express and washed again with PBS 1x. Cells were later resuspended and incubated with JC-1 in DMEM medium without phenol red + 5% (v/v) FBS for 20 min at 37ºC. The cells were then resuspended in DMEM medium without phenol red + 5% (v/v) FBS and the analysis of mitochondrial membrane potential was performed in the Attune® Acoustic Focusing Flow Cytometer (Thermo Fischer Scientific, Waltham, MA, USA). The results were processed with Attune® Cytometric software.

Evaluation of autophagy induction in HCT116DoxR cell line by flow cytometry

Autophagy induction in HCT116DoxR cell line was evaluated using the Autophagy Assay Kit (ab139484) (Abcam, Cambridge, United Kingdom) according to the manufacturers' instructions. Briefly, cells were seeded in 6-well plates at a density of 1×10^5 cells/mL and, after 24 h of incubation, culture medium was replaced by medium with IC_{50} concentrations of different Cu(II) complexes. 0.1% (v/v) DMSO, 6 μM Dox and 5 μM Cis were also used as controls. Cells were then incubated for another 48 h, and 18 h before the end of this period, the medium was replaced with a solution of medium and 1.5 μM Rapamycin (positive control). Afterwards, cells were washed with PBS 1x, detached with TrypLE^{TM} Express and washed again with DMEM medium without phenol red + 5% (v/v) FBS. After 30 min of incubation with Green Stain solution in DMEM medium without phenol red + 5% (v/v) FBS, the cells were resuspended with Assay Buffer 1x. The autophagic potential of the complexes was evaluated using the Attune® Acoustic Focusing Flow Cytometer (Thermo Fischer Scientific, Waltham, MA, USA) and the results were processed with Attune® Cytometric software.

Evaluation of ROS production in HCT116DoxR cell line

Quantification of fluorescent molecule DCF by flow cytometry

HCT116DoxR cell line was seeded in 6-well plates in a density of 1×10^5 cells/mL and incubated for 24 h. Afterwards, the culture medium was replaced by medium with IC_{50} concentrations of different Cu(II) complexes and 0.1% (v/v) DMSO, 6 μM Dox, 5 μM Cis and 42 μM Tert-Butyl hydroperoxide (TBHP) were used as controls. After an incubation period of 48 h, cells were washed with PBS 1x, detached with TrypLE™ Express and washed again with PBS 1x. Later, cells were incubated for 20 min with 10 μM of 2′,7′- dichlorodihydrofluorescein diacetate (H_2DCF-DA; Thermo Fisher Scientific, Waltham, MA, USA) in PBS 1x. ROS production was evaluated using the Attune® Acoustic Focusing Flow Cytometer (Thermo Fischer Scientific, Waltham, MA, USA) and the results were processed with Attune® Cytometric software.

Determination of the pDNA cleavage mechanisms by copper complexes *in vitro*

Plasmid DNA (pDNA) pUC18 were isolated from *E. coli* transformed cells grown overnight at 37 °C in a Luria-Bertani (LB)-agar medium (Applichem, Darmstadt, Germany) with 100 μg/ml Ampicillin (Bioline, London, United Kingdom). After the incubation period, pDNA was extracted using the NZYSpeedy Miniprep Kit (NZYTech, Portugal) according to the manufacturers' protocol. *In vitro* interaction between copper(II) complexes and pDNA was performed with 100 ng of pUC18 and increasing complex concentrations (5, 25, 50, 75, and 100 μM) in 5 mM Tris–HCl and 50 mM NaCl buffer (pH 7.02) as previously described[7]. As controls, buffered solutions of pUC18 and pUC18 + 1% (v/v) DMSO were also prepared. Each solution was programmed to a final volume of 20 μL and incubated overnight at 37ºC. In addition, a solution consisting of 100 ng of plasmid was prepared and stored at 4°C until the HindIII restriction enzyme was added during the last 2 h of incubation. Afterwards, the samples were applied to a 0.8% (w/v) agarose gel (NZYtech) and analysed by electrophoresis in TAE Buffer 1x with 0.0015% (v/v) GelRed and a constant voltage of 70 V applied for 80 min. The obtained gels were photographed and analysed on a GelDoc™ EZ Imager (Bio-Rad).

In order to determine the mechanism by which the Cu(II) complexes cleave the DNA, an assay was carried out in which 100 ng of pUC18 were exposed to the complexes and with 50 μM sodium azide (NaN_3, a ROS scavenging reagent). A positive control with 20 μM TBHP was also performed. Later, the samples were applied to a 0.8% (w/v) agarose gel (NZYtech) at the same conditions as before. The obtained gels were photographed and analysed on a GelDoc™ EZ Imager (Bio-Rad).

Cell cycle progression

HCT116DoxR cell line was seeded in 6-well plates in a density of 1×10^5 cells/mL, incubated for 8 h in a humidified atmosphere at 37ºC and 5% (v/v) CO_2, and later synchronized in early S-phase with a 2 mM thymidine solution (Merck, Germany) for a 16 h period. Later, medium with thymidine was replaced by fresh media and, after 8 h a second block with thymidine was performed in the same conditions. After 16 h of incubation, the culture medium was replaced by medium with the IC_{50} concentrations of Cu(II) complexes. 0.1% (v/v) DMSO was used as the vehicle control while 5 μM Cis and 6 μM Dox were used as the positive controls. After treatment, cells were incubated for 9, 12, 18, and 24 h. After each incubation period, cells were detached with TrypLE™ Express and centrifuged at 650 xg for 5 min, at 4ºC. The resultant pellet was resuspended in cold PBS 1x and centrifuged at 3000 xg for 5 min and 4ºC. The pellet was resuspended in cold PBS 1x, and the cells were fixed by the gentle addition of 1 mL of a solution of 80% (v/v) ethanol. Cells were stored at 4°C for at least 16 h. Afterwards, cells were centrifuged for 10 min at 7500 xg at 4°C and treated with 50 μg/mL RNAse. After 30 min of incubation at 37ºC, 25 μg/mL of PI was added to the cells. The DNA content of each sample was evaluated using the Attune® Acoustic Focusing Flow Cytometer (Thermo Fischer Scientific, Waltham, MA, USA) and the results were processed with Attune® Cytometric software.

Evaluation of cellular senescence in HCT116DoxR cell line by flow cytometry

The effect of the copper complexes in cellular senescence was evaluated using the Senescence Assay Kit (Beta Galactosidase Fluorescence, Abcam, Cambridge, UK). HCT116DoxR cell line was seeded

in 24-well plates in a density of 5×10^5 cells/mL and incubated for 24 h in a humidified atmosphere at 37ºC and 5% (v/v) CO_2. The culture medium was then replaced by medium with the Cu(II) complexes at its IC_{50} concentrations. 0.1% (v/v) DMSO was used as the vehicle control while 5 μM Cis and 6 μM Dox were used as the positive controls. After a 48 h incubation period, the medium in each well was replaced by the senescence probe in fresh medium and cells were incubated in the same conditions for 1.5 h. Afterwards, cells were washed with wash buffer, detached with TrypLE™ Express and washed twice again with wash buffer. The percentage of fluorescent cells was then determined using the Attune® Acoustic Focusing Flow Cytometer (Thermo Fischer Scientific, Waltham, MA, USA) and the results were processed with Attune® Cytometric software.

BSA binding assays

All solutions used in the BSA interaction studies were prepared with a 20 μM fixed final concentration of BSA and variable complex concentrations (10, 25, and 50 μM), diluted in filtered buffer (10 mM phosphate and 150 mM NaCl) at pH 7.0. Control solutions were also prepared, namely a solution containing only BSA, a solution consisting of BSA and DMSO, a solution consisting of DMSO only, and a solution containing only each Cu(II) complex at each of the mentioned concentrations. Solutions were analysed after 24 h of incubation at 37ºC. The UV-Vis spectra were obtained from 245 nm to 500 nm on the spectrophotometer Evolution 300 UV-Vis (Thermo Fischer Scientific, Waltham, MA, USA) while the fluorescence spectra were recorded on a Varian Cary Eclipse fluorescence spectrophotometer (Agilent Technologies, CA, USA) through an emission range of 290 to 500 nm after an excitation wavelength of 278 nm and with an excitation and emission slit widths of 5 nm. The affinity constant (K_b) was calculated using the Stern–Volmer equation[8] relating the fluorescence of protein in the absence and in the presence of the complexes:

$$log\frac{(F_0 - F)}{F} = logK_b + nlog[complex]$$

where F and F_0 represent the fluorescence intensities with and without the complexes, respectively, K_b represents the binding constant and n the number of binding sites.

Calf Thymus DNA binding assays

The DNA-binding experiments were carried out in PBS (pH 7.4, 130 mM NaCl) at rt. Concentrated stock solutions of Cu(II) compounds were prepared by dissolving the complex in 0.1 ml of DMSO and diluted with PBS to concentrations suitable for all the experiments, and the DMSO content in final samples was no more than 0.1%. Concentrated **s**tock solution of calf thymus DNA (ct-DNA, Invitrogen, Thermo Fischer Scientific, Waltham, MA, USA) was stored at 4 ºC and used in less than 4 days. The concentration of ct-DNA was determined by UV-Vis absorbance at 260 nm (A_{260}), with molar absorption coefficient ε_{260} = 6600 $M^{-1}\cdot cm^{-1}$ [9]. The ratio of absorbances of ct-DNA solution at 260 and 280 nm, A_{260}/A_{280} was approximately 1.89, indicating that the DNA was sufficiently free of protein[10]. The absorption titration of complexes with ct-DNA were carried out on NanoDrop OneC UV-Vis spectrophotometer (Thermo Fischer Scientific, Waltham, MA, USA) with use of cuvette measuring mode (l = 1.0 cm) and magnetic stirring at 37 °C, measured from 250 nm to 500 nm. The samples of complexes (maintaining constant concentration of 10 or 25 μM) in PBS were titrated with increasing concentrations of nucleic acid (0–350 μM). The equal amounts of ct-DNA were added to both the complex and the reference solutions to eliminate the absorbance of DNA itself. Samples were incubated for 8 min to equilibrate before each measurement. The intrinsic binding constant K_b was determined using the Wolfe-Shimer equation:

$$\frac{[DNA]}{[\varepsilon_a - \varepsilon_f]} = \frac{[DNA]}{[\varepsilon_b - \varepsilon_f]} + \frac{1}{K_b[\varepsilon_b - \varepsilon_f]}$$

where the absorption coefficients εa, εf and εb correspond to A_{obsd}/[complex], the extinction coefficient for the free compound and for the compound in the fully bound form, respectively. The

intrinsic binding constant K_b can be obtained from the ratio of the slope to the intercept of the linear fit of the plot [DNA]/[ε_a–ε_f] versus [DNA].

For emission quenching experiments, equimolar solution of ct-DNA and ethidium bromide (EB, Sigma Aldrich) was prepared and allowed to incubate for 2 h in the dark. The tested complexes were titrated into the formed EB–ct-DNA adduct and incubated for additional 30 min. The final concentration of the EB–ct-DNA adduct was 50 μM and the concentration of the complexes varied from 0 to 40 μM. The fluorescence spectra were recorded on FLS-980 spectrofluorometer (Edinburgh Instruments, Livingston, United Kingdom) with excitation wavelength 515 nm and emission wavelength of 620 nm. The apparent binding constant (K_{app}) was calculated using the Equations :

$$I_0/I = 1 + K_{SV}[Q]$$

$$K_{app}[Q_{1/2}] = K_{EB}[EB]$$

where I_0 and I are the fluorescence intensity in the absence and presence of the complexes; K_{SV} is the Stern-Volmer quenching constant; [Q] is the concentration of the complexes; [$Q_{1/2}$] is the concentration of the complex causing 50% reduction in the fluorescence intensity; K_{EB} = 1×10^{7} M^{-1}; [EB] = 5×10^{-5} M.

Cell Migration assay

Fibroblasts were seeded in 24-well plates in a density of 1×10^{5} cells/mL and grown ate the same conditions mentioned before until the presence of a confluent monolayer was detected. A scratch was made on the surface of each well using a sterile 200 μL micropipette tip and the cells were immediately exposed to medium with the IC_{50} concentrations of Cu(II) complexes, 0.1% (v/v) DMSO (vehicle control) or 0.4 μM Dox. Each plate was then incubated for 24 h in the same conditions mentioned before. Fibroblasts were photographed immediately after exposure to the complexes or controls (0 h) and after incubation (24 h) using a Ti-U Eclipse inverted microscope (Nikon, Japan). The width of each scratch in each image was measured via ImageJ software.

***Ex-ovo* Chick Chorioallantoic Membrane (CAM) assay**

The *ex-ovo* CAM assay was performed to evaluate the copper(II) complexes' effect on angiogenesis, as already described in literature[11,12]. Firstly, chicken embryos were incubated for 24 h at 37ºC in a humidified atmosphere. After embryo stabilization, a solution containing the IC_{50} concentration of each copper complex or 0.1% DMSO (vehicle control) dissolved in PBS 1x was placed in the center of O-rings. Each O-ring was photographed immediately after exposure to the complexes or DMSO (0 h) and after 24 h and 48 h incubation at 37ºC using a digital USB Microscope Camera (Opti-Tekscope OT-V1). Newly formed blood vessels were manually counted via ImageJ software.

The *ex-ovo* CAM assay fulfils the Directive 2010/63/ EU of the European Parliament for the protection of animal models for scientific purposes.

Statistical analysis

All results were expressed as mean ± SEM of at least two independent biological assays, each obtained by technical duplicates, unless otherwise specified. One-way ANOVA or Student's t-test were used to determine statistical significance ($p < 0.05$) using the GraphPad Prism 8 software (GraphPad Software Inc., San Diego, CA, USA).

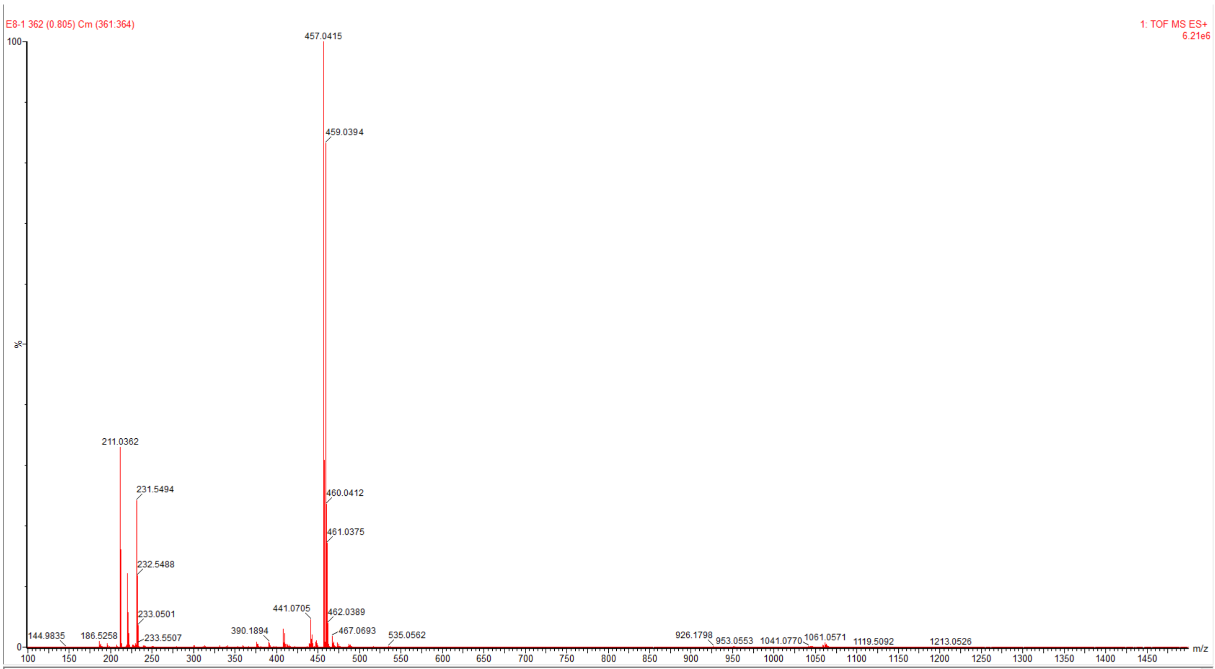

Multiple Mass Analysis: 2 mass(es) processed
Tolerance = 200.0 PPM / DBE: min = -10.0, max = 100.0
Element prediction: Off
Number of isotope peaks used for i-FIT = 2
Monoisotopic Mass, Even Electron Ions
24 formula(e) evaluated with 3 results within limits (up to 2 closest results for each mass)
Elements Used:

Mass	RA	Calc. Mass	mDa	PPM	DBE	Formula	i-FIT	i-FIT Norm	Fit Conf %	C	H	N	Cl	Cu
457.0415	100.00	457.0407	0.8	1.8	18.5	C25 H17 N3 Cl Cu	599.7	n/a	n/a	25	17	3	1	1
459.0394	83.49	459.0564	-17.0	-37.0	17.5	C25 H19 N3 Cl Cu	585.9	0.000	100.00	25	19	3	1	1
		458.9625	76.9	167.5	24.5	C26 H7 N3 Cl Cu	596.8	10.977	0.00	26	7	3	1	1

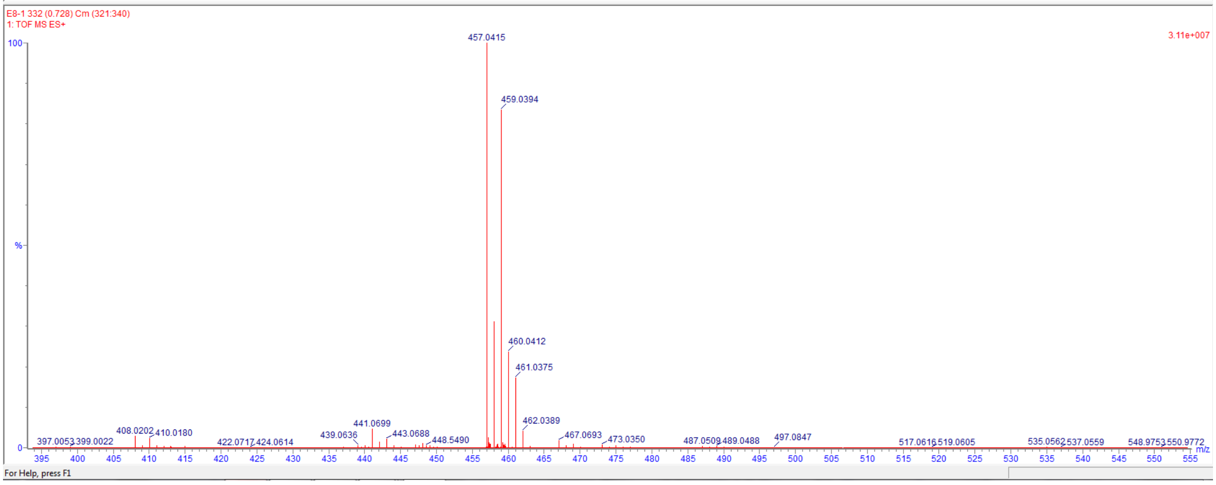

1

HRMS (ESI): calcd for $C_{25}H_{17}N_3ClCu^+$ 457.0407 found 457.0415.

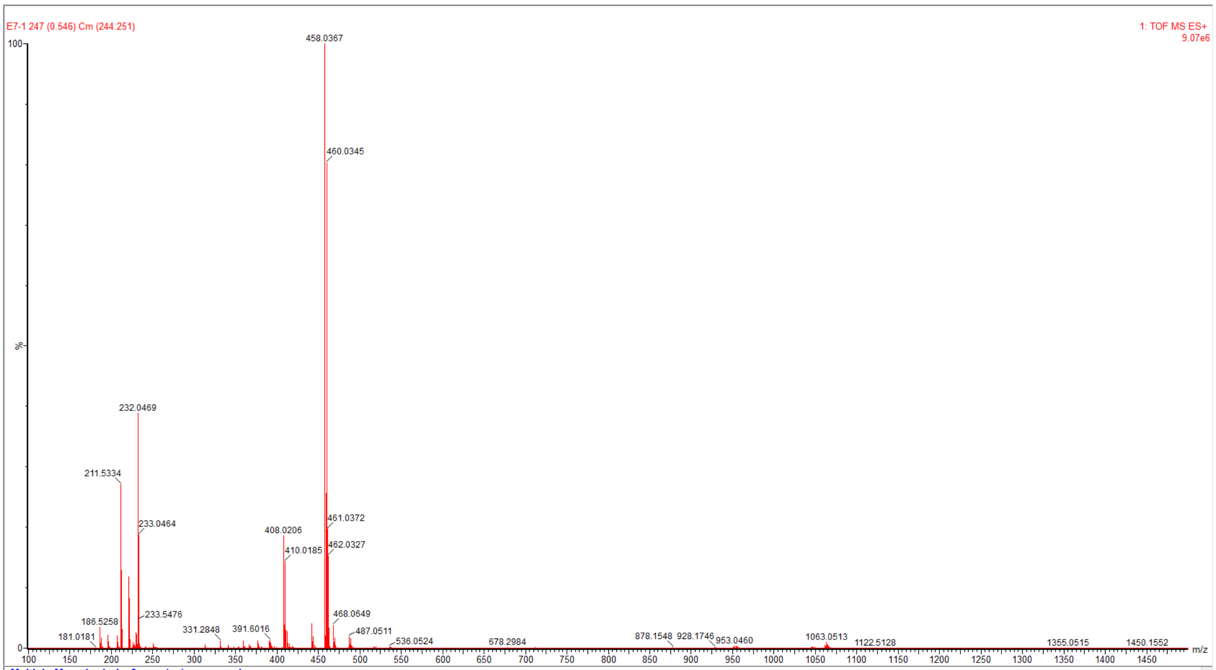

Multiple Mass Analysis: 2 mass(es) processed
Tolerance = 100.0 PPM / DBE: min = -10.0, max = 100.0
Element prediction: Off
Number of isotope peaks used for i-FIT = 2
Monoisotopic Mass, Even Electron Ions
130 formula(e) evaluated with 11 results within limits (up to 2 closest results for each mass)
Elements Used:

Mass	RA	Calc. Mass	mDa	PPM	DBE	Formula	i-FIT	i-FIT Norm	Fit Conf %	C	H	N	Cl	Cu
458.0367	100.00	458.0359	0.8	1.7	18.5	C24 H16 N4 Cl Cu	518.9	0.004	99.56	24	16	4	1	1
		458.0157	21.0	45.8	27.5	C32 H11 Cu	524.4	5.437	0.44	32	11			1
460.0345	80.39	460.0313	3.2	7.0	26.5	C32 H13 Cu	505.9	3.230	3.95	32	13			1
		460.0516	-17.1	-37.2	17.5	C24 H18 N4 Cl Cu	502.7	0.040	96.05	24	18	4	1	1

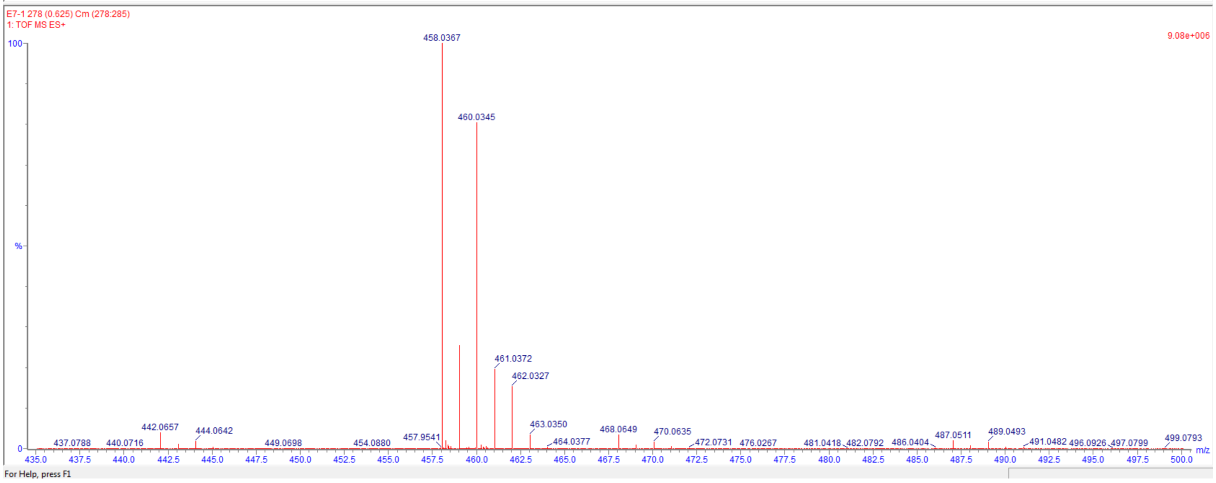

2

HRMS (ESI): calcd for $C_{24}H_{16}N_4ClCu^+$ 458.0359 found 458.0367.

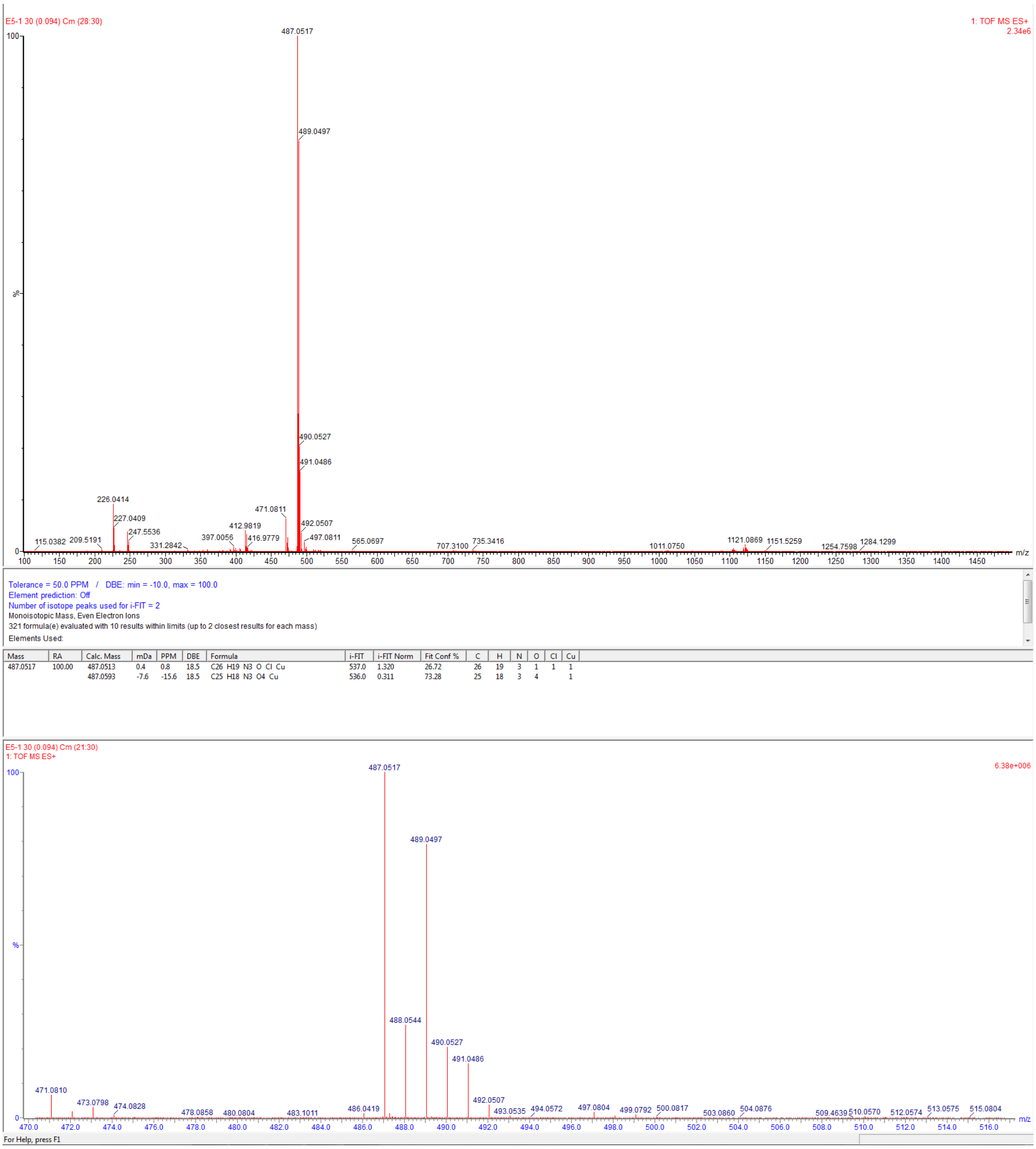

3

HRMS (ESI): calcd for $C_{26}H_{19}N_3OClCu^+$ 487.0513 found 487.0517.

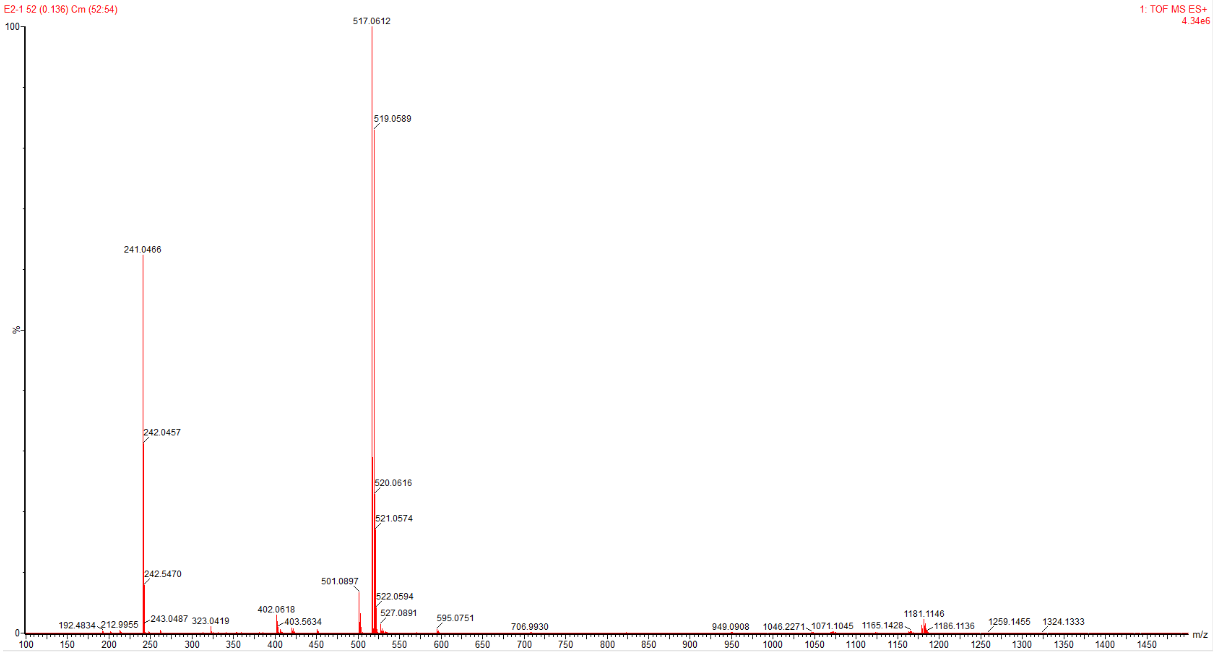

Multiple Mass Analysis: 2 mass(es) processed
Tolerance = 200.0 PPM / DBE: min = -10.0, max = 100.0
Element prediction: Off
Number of isotope peaks used for i-FIT = 2
Monoisotopic Mass, Even Electron Ions
669 formula(e) evaluated with 89 results within limits (up to 2 closest results for each mass)
Elements Used:

Mass	RA	Calc. Mass	mDa	PPM	DBE	Formula	i-FIT	i-FIT Norm	Fit Conf %	C	H	N	O	Cl	Cu
517.0612	100.00	517.0618	-0.6	-1.2	18.5	C27 H21 N3 O2 Cl Cu	517.0	0.012	98.76	27	21	3	2	1	1
		517.0640	-2.8	-5.4	27.5	C33 H16 N3 Cu	521.4	4.389	1.24	33	16	3			1
519.0589	83.20	519.0644	-5.5	-10.6	22.5	C29 H18 N3 O3 Cu	498.0	0.479	61.93	29	18	3	3		1
		519.0564	2.5	4.8	22.5	C30 H19 N3 Cl Cu	498.5	0.966	38.07	30	19	3		1	1

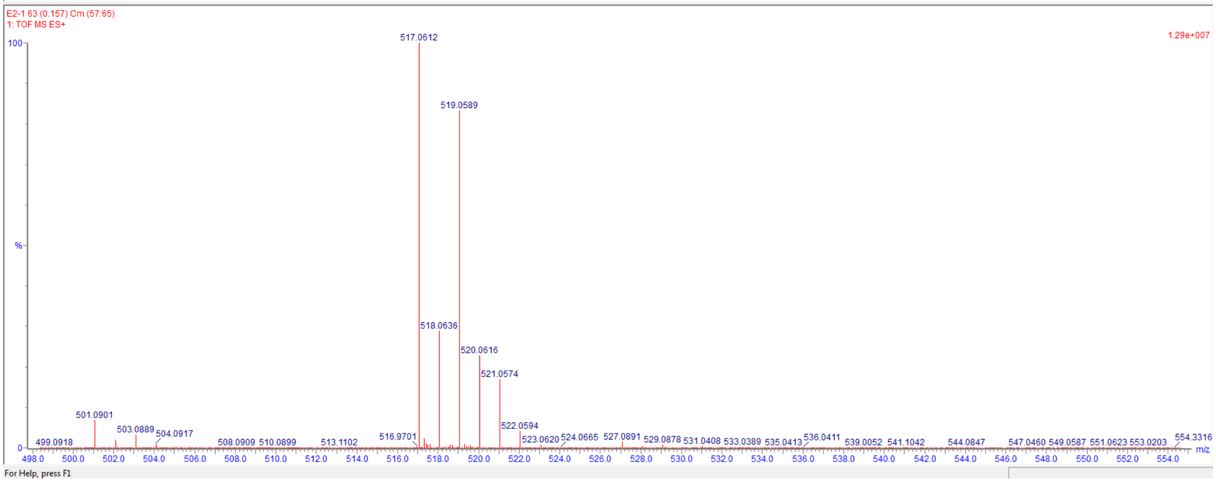

For Help, press F1

4

HRMS (ESI): calcd for $C_{27}H_{21}N_3O_2ClCu^+$ 517.0618 found 517.0612.

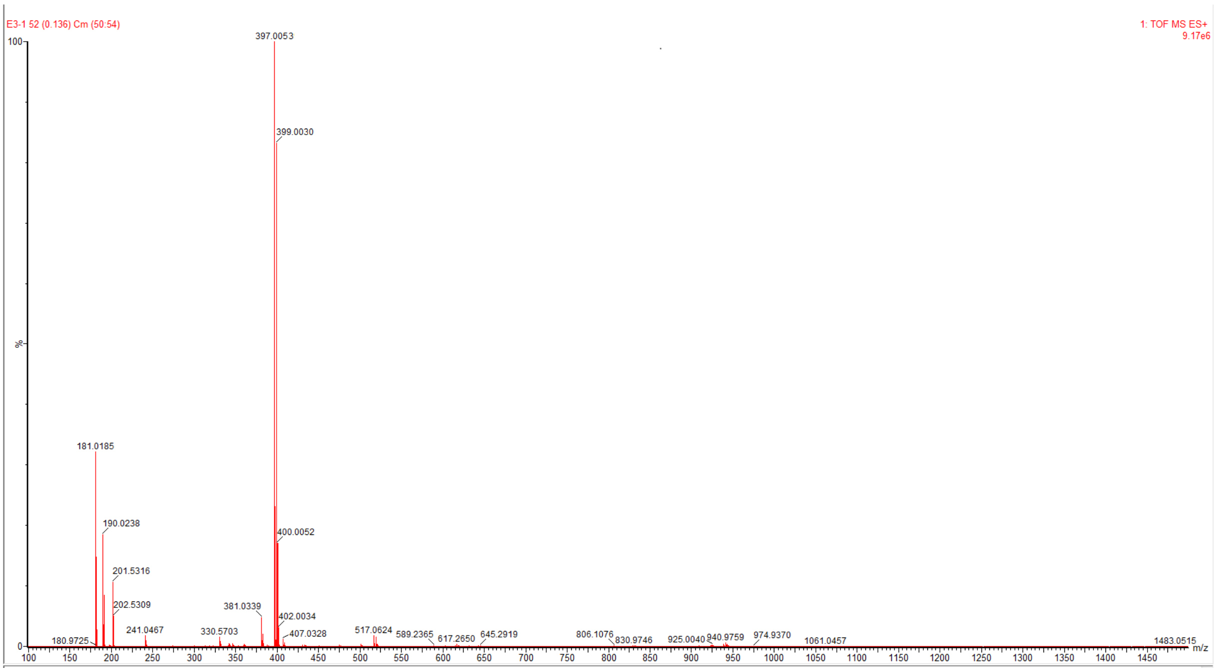

Multiple Mass Analysis: 2 mass(es) processed
Tolerance = 50.0 PPM / DBE: min = -10.0, max = 100.0
Element prediction: Off
Number of isotope peaks used for i-FIT = 2
Monoisotopic Mass, Even Electron Ions
537 formula(e) evaluated with 17 results within limits (up to 2 closest results for each mass)
Elements Used:

Mass	RA	Calc. Mass	mDa	PPM	DBE	Formula	i-FIT	i-FIT Norm	Fit Conf %	C	H	N	O	Cl	Cu
397.0053	100.00	397.0043	1.0	2.5	14.5	C19 H13 N3 O Cl Cu	563.6	0.128	87.98	19	13	3	1	1	1
		397.0124	-7.1	-17.9	14.5	C18 H12 N3 O4 Cu	565.6	2.119	12.02	18	12	3	4		1
399.0030	83.56	399.0047	-1.7	-4.3	9.5	C15 H15 N3 O4 Cl Cu	546.1	0.295	74.46	15	15	3	4	1	1
		399.0069	-3.9	-9.8	18.5	C21 H10 N3 O2 Cu	547.2	1.365	25.54	21	10	3	2		1

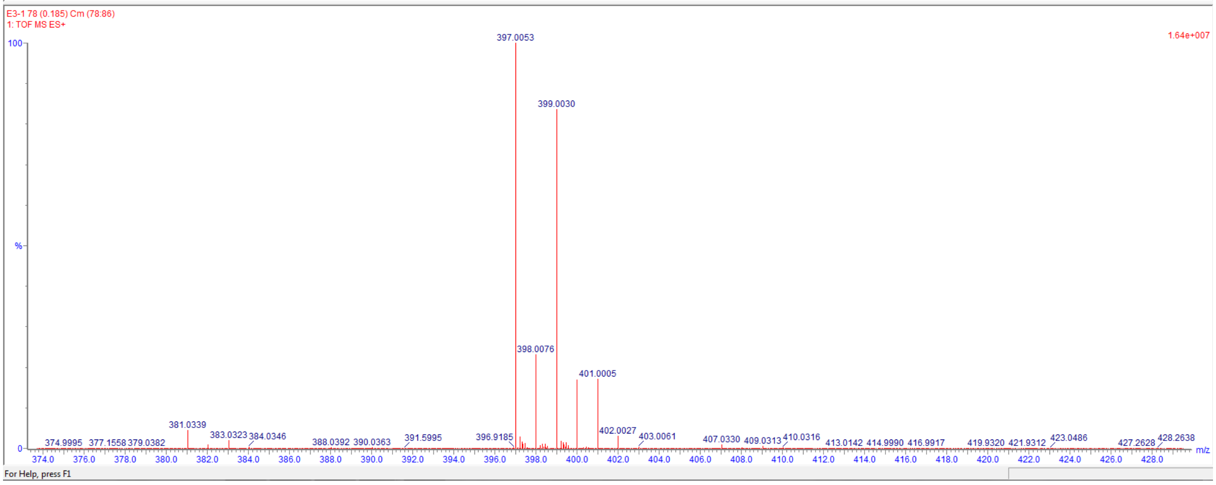

5

HRMS (ESI): calcd for $C_{19}H_{13}N_3OClCu^+$ 397.0043 found 397.0053.

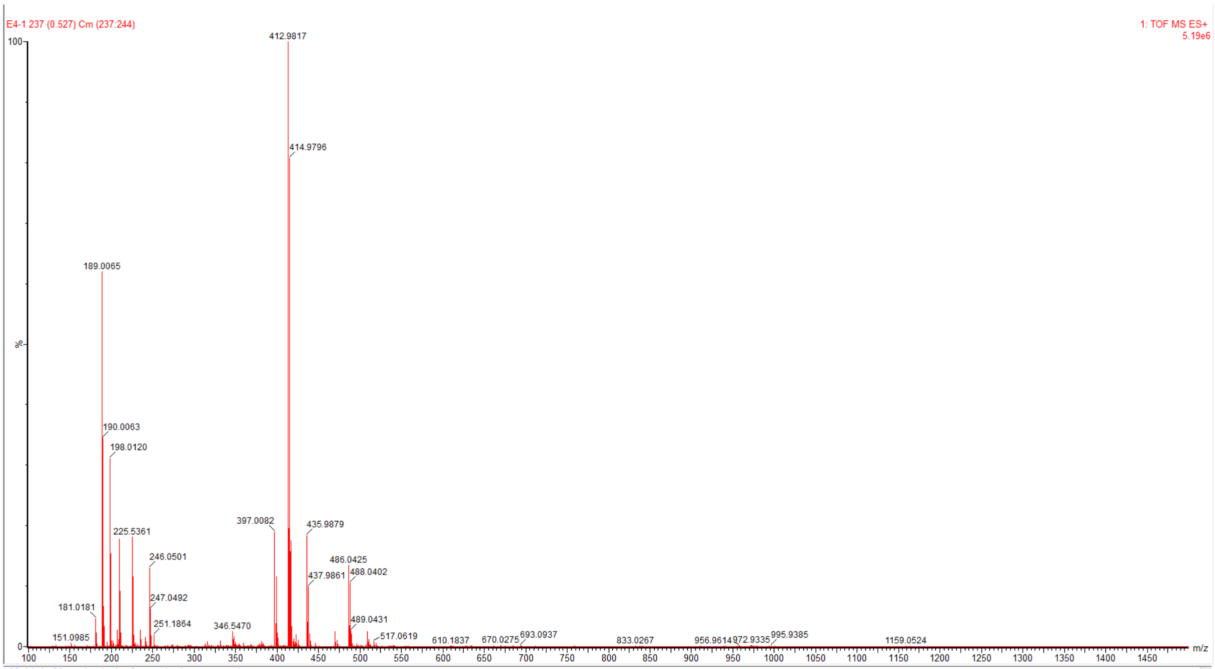

Multiple Mass Analysis: 2 mass(es) processed
Tolerance = 50.0 PPM / DBE: min = -10.0, max = 100.0
Element prediction: Off
Number of isotope peaks used for i-FIT = 2
Monoisotopic Mass, Even Electron Ions
335 formula(e) evaluated with 6 results within limits (up to 2 closest results for each mass)
Elements Used:

Mass	RA	Calc. Mass	mDa	PPM	DBE	Formula	i-FIT	i-FIT Norm	Fit Conf %	C	H	N	S	Cl	Cu
412.9817	100.00	412.9815	0.2	0.5	14.5	C19 H13 N3 S Cl Cu	533.4	4.132	1.60	19	13	3	1	1	1
		412.9848	-3.1	-7.5	9.5	C16 H17 N3 S2 Cl Cu	529.3	0.016	98.40	16	17	3	2	1	1
414.9796	80.87	414.9938	-14.2	-34.2	18.5	C22 H11 N3 Cl Cu	535.0	1.577	20.67	22	11	3		1	1
		414.9971	-17.5	-42.2	13.5	C19 H15 N3 S Cl Cu	533.6	0.232	79.33	19	15	3	1	1	1

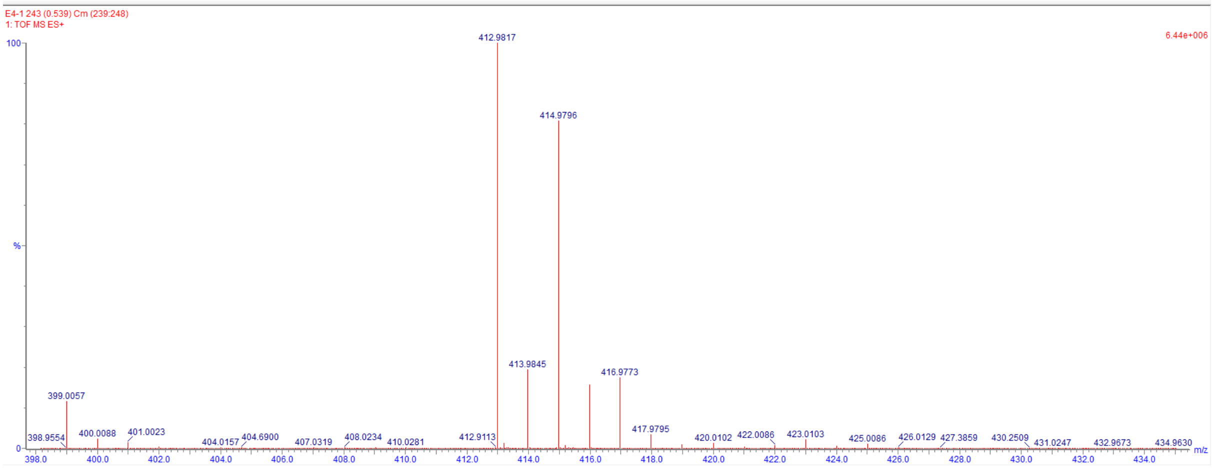

6

HRMS (ESI): calcd for $C_{19}H_{13}N_3SClCu^+$ 412.9815 found 412.9817.

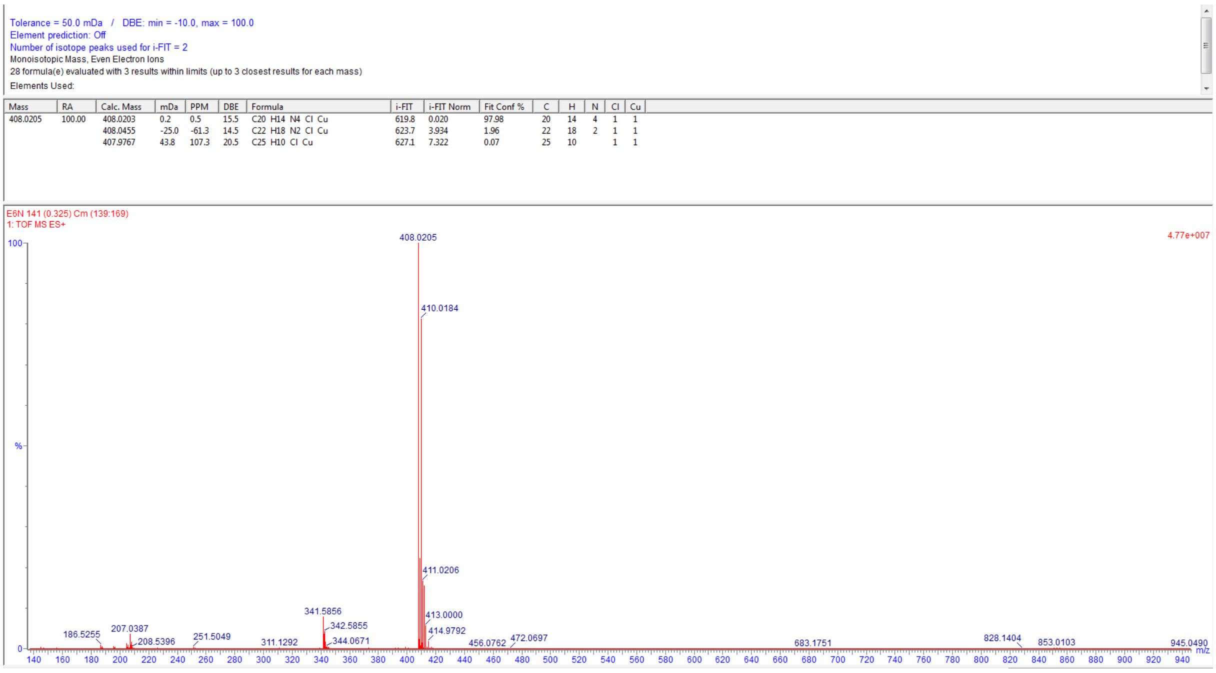

Tolerance = 50.0 mDa / DBE: min = -10.0, max = 100.0
Element prediction: Off
Number of isotope peaks used for i-FIT = 2
Monoisotopic Mass, Even Electron Ions
28 formula(e) evaluated with 3 results within limits (up to 3 closest results for each mass)
Elements Used:

Mass	RA	Calc. Mass	mDa	PPM	DBE	Formula	i-FIT	i-FIT Norm	Fit Conf %	C	H	N	Cl	Cu
408.0205	100.00	408.0203	0.2	0.5	15.5	C20 H14 N4 Cl Cu	619.8	0.020	97.98	20	14	4	1	1
		408.0455	-25.0	-61.3	14.5	C22 H18 N2 Cl Cu	623.7	3.934	1.96	22	18	2	1	1
		407.9767	43.8	107.3	20.5	C25 H10 Cl Cu	627.1	7.322	0.07	25	10		1	1

7

HRMS (ESI): calcd for $C_{20}H_{14}N_4ClCu^+$ 408.0203 found 408.0205.

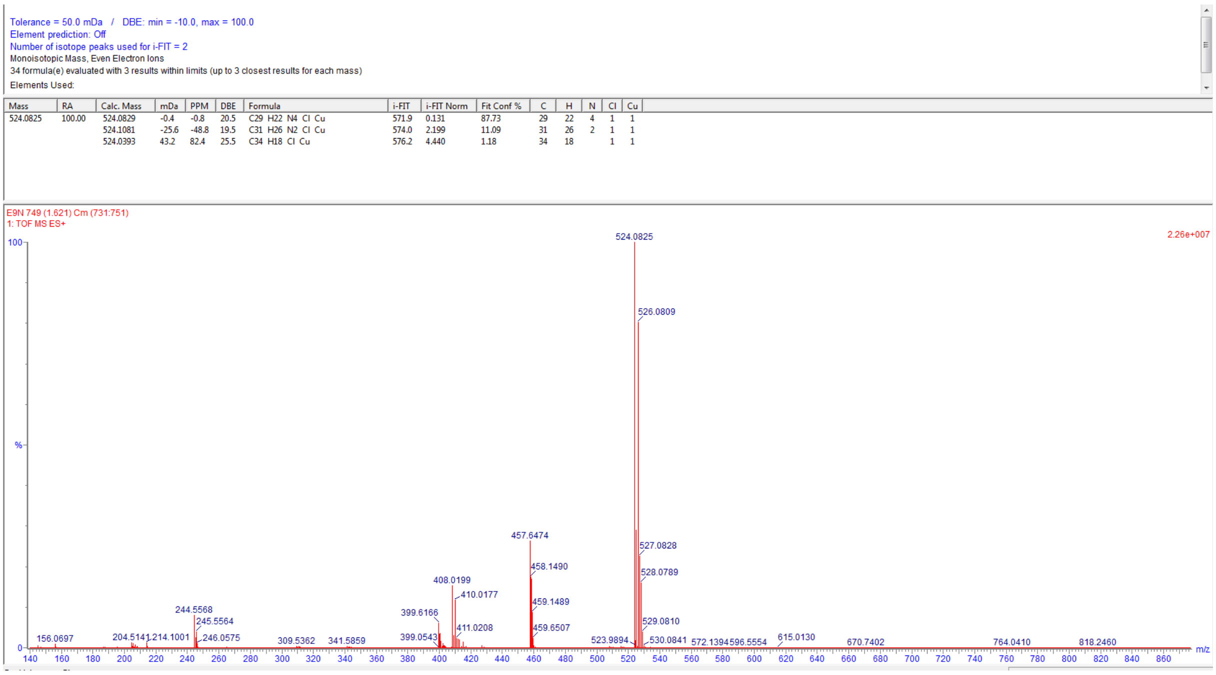

Tolerance = 50.0 mDa / DBE: min = -10.0, max = 100.0
Element prediction: Off
Number of isotope peaks used for i-FIT = 2
Monoisotopic Mass, Even Electron Ions
34 formula(e) evaluated with 3 results within limits (up to 3 closest results for each mass)
Elements Used:

Mass	RA	Calc. Mass	mDa	PPM	DBE	Formula	i-FIT	i-FIT Norm	Fit Conf %	C	H	N	Cl	Cu
524.0825	100.00	524.0829	-0.4	-0.8	20.5	C29 H22 N4 Cl Cu	571.9	0.131	87.73	29	22	4	1	1
		524.1081	-25.6	-48.8	19.5	C31 H26 N2 Cl Cu	574.0	2.199	11.09	31	26	2	1	1
		524.0393	43.2	82.4	25.5	C34 H18 Cl Cu	576.2	4.440	1.18	34	18		1	1

8

HRMS (ESI): calcd for $C_{29}H_{22}N_4ClCu^+$ 524.0829 found 524.0825.

Figure S1. HR-ESI-MS spectra of the complexes **1-8**.

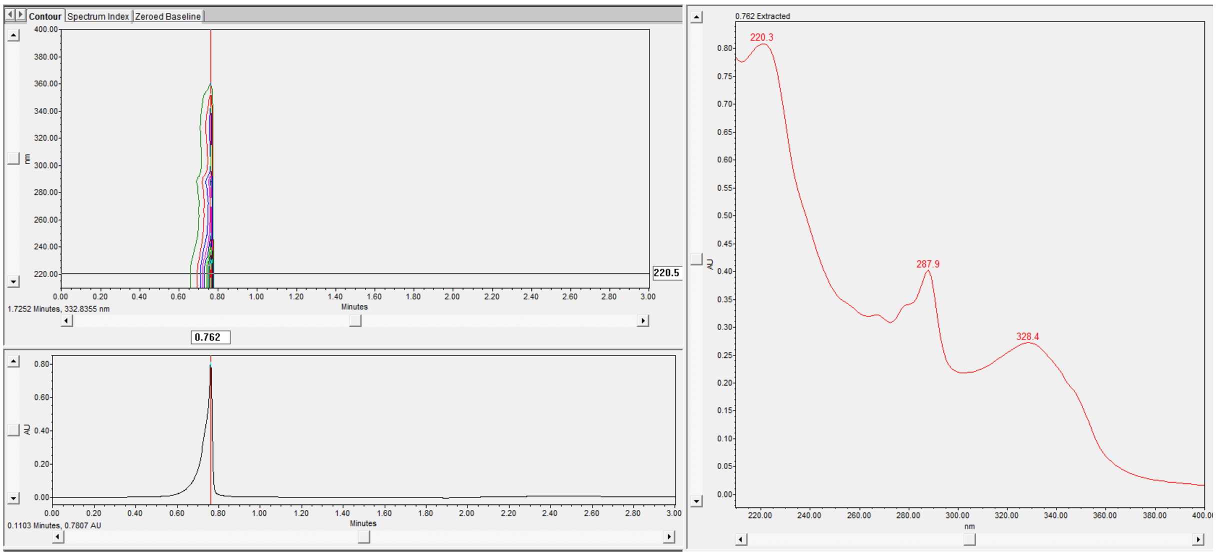
Contour
Spectrum Index
Zoomed Baseline
220.5
0.762
1.7252 Minutes, 332.8355 nm
0.1103 Minutes, 0.7807 AU
Minutes
0.762 Extracted
220.3
287.9
328.4
nm

1

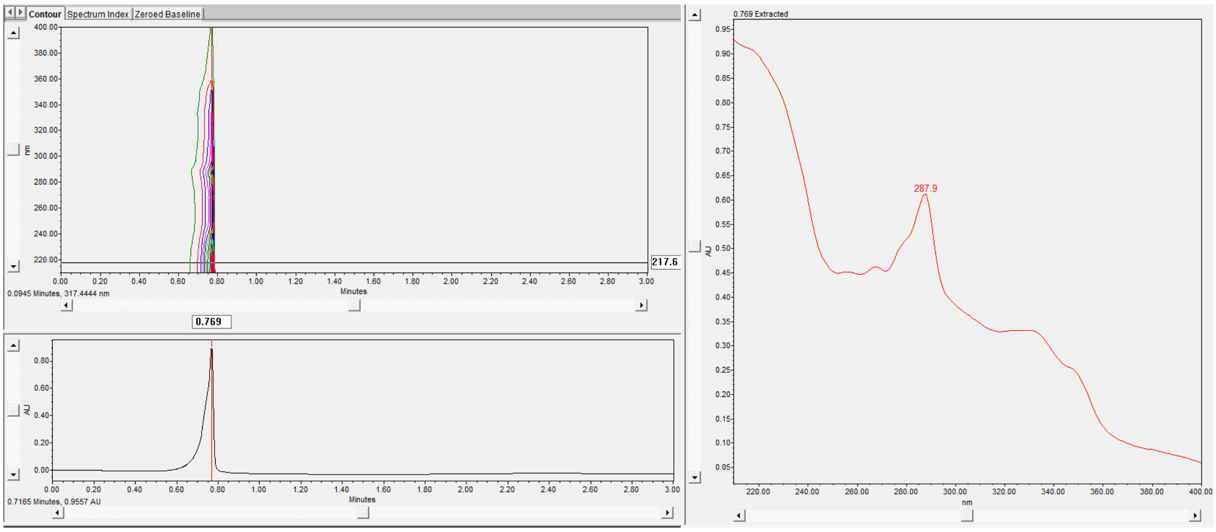
Contour
Spectrum Index
Zoomed Baseline
217.6
0.769
0.0945 Minutes, 317.4444 nm
0.7165 Minutes, 0.9557 AU
Minutes
0.769 Extracted
287.9
nm

2

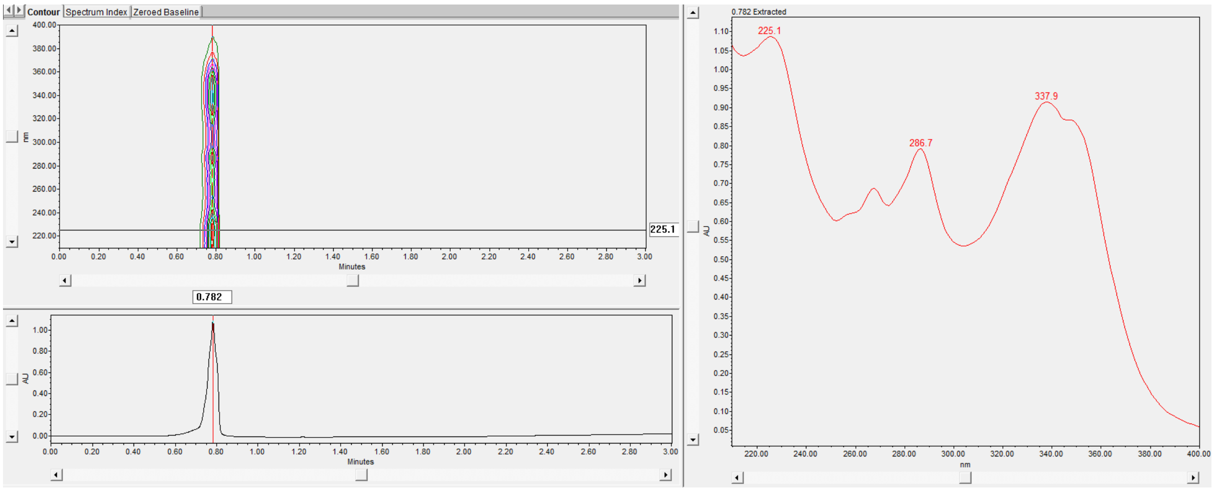
Contour
Spectrum Index
Zoomed Baseline
225.1
0.782
Minutes
0.782 Extracted
225.1
286.7
337.9
nm

3

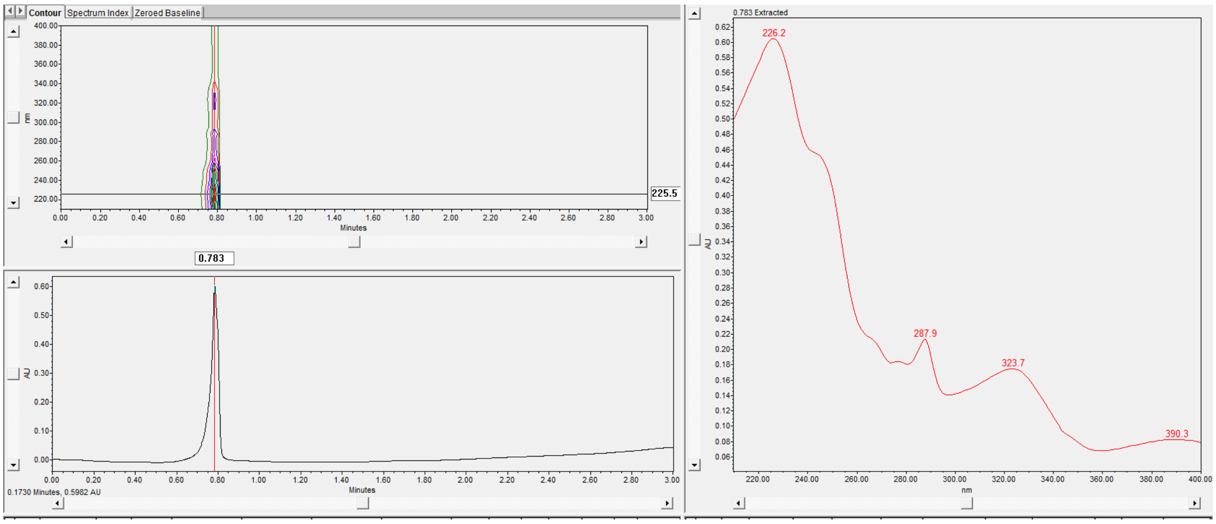
Contour
Spectrum Index
Zeroed Baseline
225.5
0.783
Minutes
0.1730 Minutes, 0.5982 AU
AU
0.783 Extracted
226.2
287.9
323.7
390.3
nm

4

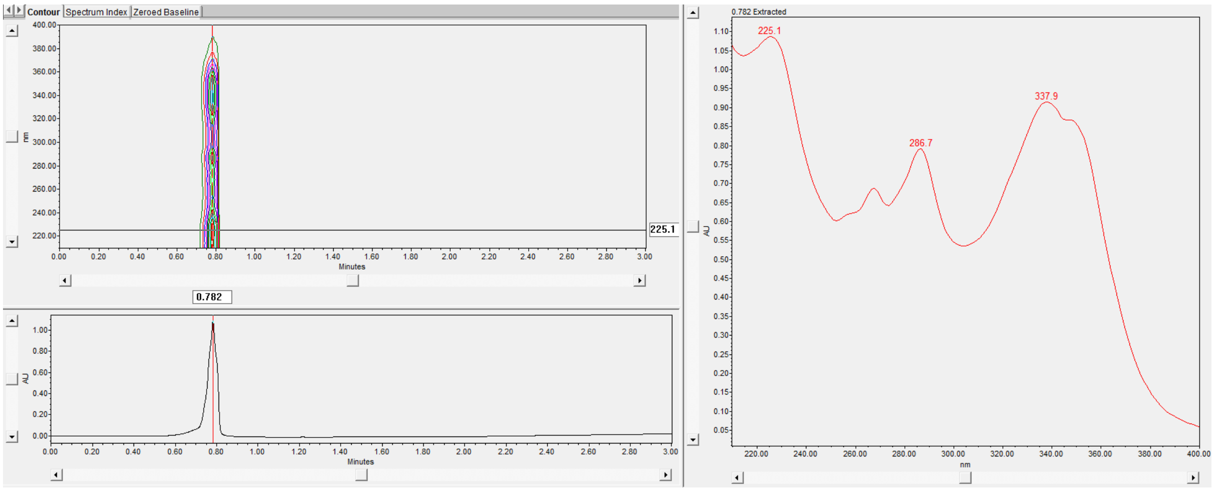
Contour
Spectrum Index
Zeroed Baseline
225.1
0.782
Minutes
AU
0.782 Extracted
225.1
286.7
337.9
nm

5

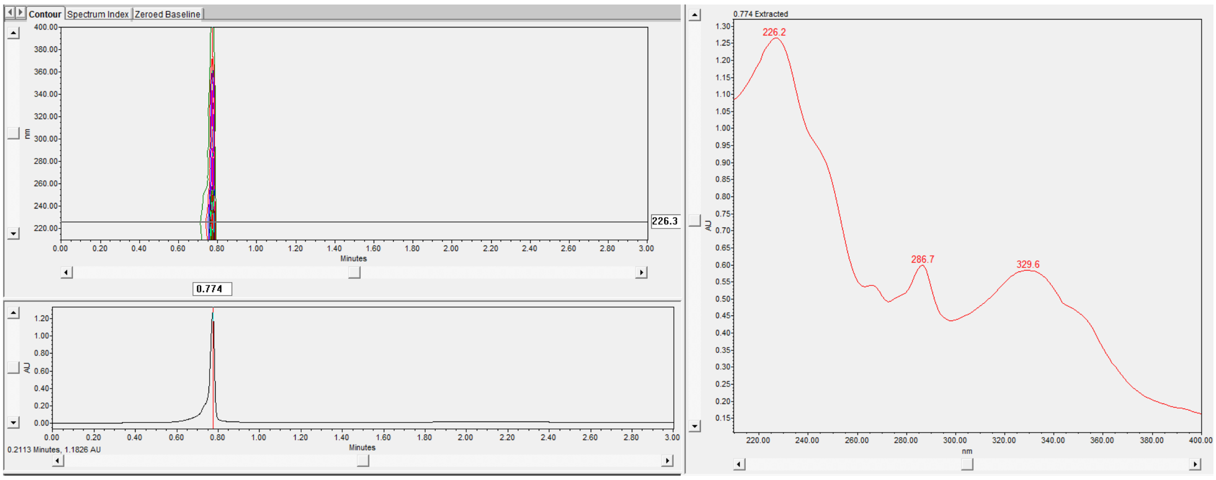
Contour
Spectrum Index
Zeroed Baseline
226.3
0.774
Minutes
0.2113 Minutes, 1.1826 AU
AU
0.774 Extracted
226.2
286.7
329.6
nm

6

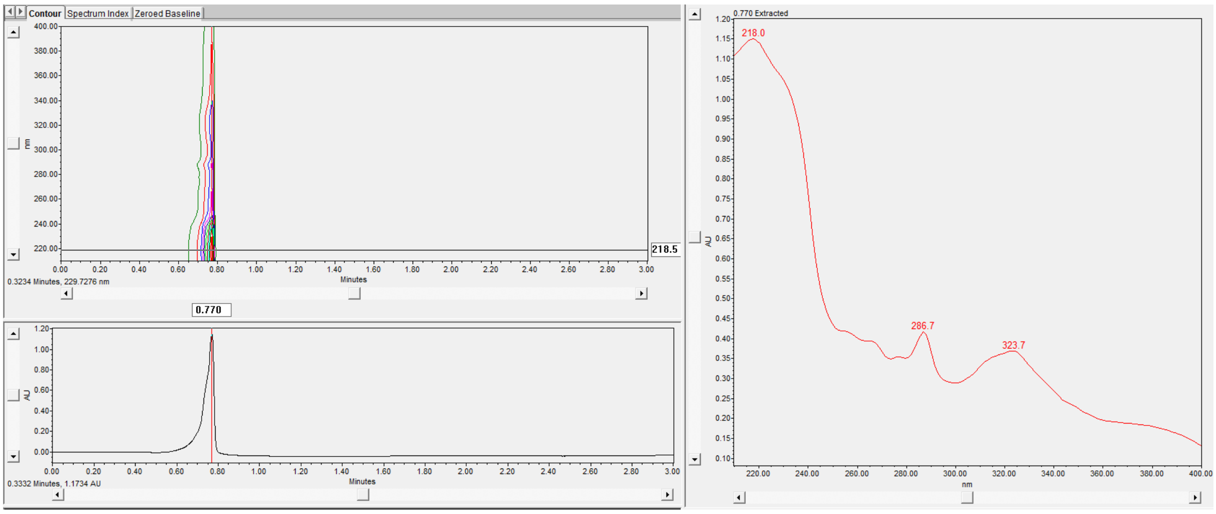

7

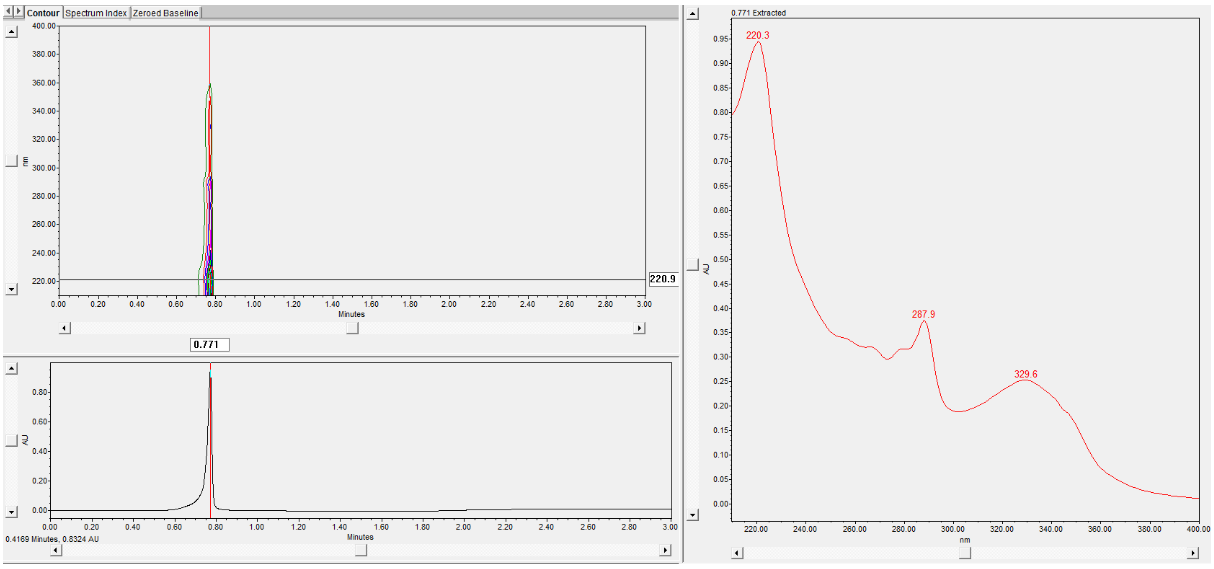

8

Figure S2. UPLC of the complexes **1-8**. Eluent actonitrile:water mixture (80:20, v/v), injection 2 μl, flow 0.30 ml/min, PDA detector.

Table S1. Crystal data and structure refinement.

Complex	1	2	3	4	5	6	7	8
**Empirical formula**	$C_{50}H_{34}Cl_2Cu_2F_{12}N_6P_2$ + masked solvent	$C_{52}H_{38}Cl_2Cu_2F_{12}N_{10}P_2$	$C_{52}H_{38}Cl_2Cu_2F_{12}N_6O_2P_2$	$C_{54}H_{42}Cl_2Cu_2F_{12}N_6O_4P_2$ + masked solvent	$C_{38}H_{26}Cl_2Cu_2F_{12}N_6O_2P_2$	$C_{38}H_{26}Cl_2Cu_2F_{12}N_6P_2S_2$	$C_{40}H_{28}Cl_2Cu_2F_{12}N_8P_2$	$C_{58}H_{44}Cl_2Cu_2F_{12}N_8P_2$ + masked solvent
**Formula weight**	1206.75	1290.84	1266.80	1326.85	1086.57	1118.69	1108.62	1340.93
**Temperature [K]**	295.0(2)	295.0(2)	295.0(2)	295.0(2)	295.0(2)	295.0(2)	295.0(2)	295.0(2)
**Wavelength [Å]**	0.71073	0.71073	0.71073	0.71073	0.71073	0.71073	0.71073	0.71073
**Crystal system**	Monoclinic	Monoclinic	Monoclinic	Triclinic	Monoclinic	Monoclinic	Monoclinic	Monoclinic
**Space group**	$P2_1/n$	$P2_1/n$	$P2_1/n$	$P\bar{1}$	$P2_1/c$	$P2_1/c$	$P2_1/c$	$P2/n$
**Unit cell dimensions [Å,°]**	a = 13.5489(8)	a = 13.4845(6)	a = 13.7501(14)	a = 8.0166(4)	a = 6.5051(5)	a = 6.6795(6)	a = 6.6743(3)	a = 15.1332(7)
	b = 7.6952(4)	b = 7.8492(3)	b = 7.7288(10)	b = 13.6030(5)	b = 20.1133(12)	b = 19.8481(16)	b = 16.9776(7)	b = 12.4816(7)
	c = 24.8745(13)	c = 24.4501(8)	c = 24.965(2)	c = 25.1535(10)	c = 15.2396(8)	c = 15.6455(15)	c = 18.7028(8)	c = 17.1567(8)
				α = 91.416(3)				
	β = 94.736(5)	β = 96.475(4)	β = 92.766(9)	β = 91.495(4)	β = 94.115(6)	β = 92.455(9)	β = 97.174(4)	β = 112.173(5)
				γ = 97.962(3)				
**Volume [Å^3]**	2584.6(2)	2571.35(17)	2649.9(5)	2714.5(2)	1988.8(2)	2072.3(3)	2102.69(16)	3001.0(3)
**Z**	2	2	2	2	2	2	2	2
**Density (calculated) [Mg/m^3]**	1.551	1.667	1.588	1.623	1.814	1.793	1.751	1.484
**Absorption coefficient [mm-1]**	1.073	1.087	1.053	1.035	1.387	1.427	1.311	0.933
**F(000)**	1212	1300	1276	1340	1084	1116	1108	1356
**Crystal size [mm]**	0.37 × 0.07 × 0.02	0.43 × 0.04 × 0.04	0.14 × 0.09 × 0.09	0.36 × 0.04 × 0.03	0.06 × 0.05 × 0.03	0.12 × 0.03 × 0.03	0.54 × 0.05 × 0.05	0.32 × 0.04 × 0.04
**θ range for data collection [°]**	3.52 to 25.05	3.36 to 25.05	3.32 to 25.05	3.39 to 25.05	3.32 to 25.05	3.32 to 25.05	3.50 to 25.05	3.51 to 25.05
**Index ranges**	-16 ≤ h ≤ 13<br>-9 ≤ k ≤ 9<br>-29 ≤ l ≤ 25	-16 ≤ h ≤ 15<br>-9 ≤ k ≤ 8<br>-29 ≤ l ≤ 22	-11 ≤ h ≤ 16<br>-8 ≤ k ≤ 9<br>-29 ≤ l ≤ 29	-9 ≤ h ≤ 9<br>-16 ≤ k ≤ 15<br>-29 ≤ l ≤ 29	-7 ≤ h ≤ 7<br>-23 ≤ k ≤ 21<br>-18 ≤ l ≤ 15	-7 ≤ h ≤ 7<br>-23 ≤ k ≤ 22<br>-18 ≤ l ≤ 18	-7 ≤ h ≤ 7<br>-19 ≤ k ≤ 20<br>-22 ≤ l ≤ 21	-18 ≤ h ≤ 17<br>-14 ≤ k ≤ 10<br>-17 ≤ l ≤ 20
**Reflections collected**	12315	11374	12079	22475	9709	10209	10491	13598
**Independent reflections**	4570 (R_{int} = 0.0580)	4544 (R_{int} = 0.0250)	4680 (R_{int} = 0.0746)	9593 (R_{int} = 0.0423)	3521 (R_{int} = 0.0364)	3637 (R_{int} = 0.0765)	3713 (R_{int} = 0.0304)	5302 (R_{int} = 0.0263)
**Completeness to 2θ**	99.7%	99.7%	99.7%	99.7%	99.8%	99.5%	99.8%	99.7%
**Min. and max. transm.**	0.698 and 1.000	0.850 and 1.000	0.706 and 1.000	0.754 and 1.000	0.554 and 1.000	0.141 and 1.000	0.582 and 1.000	0.624 and 1.000
**Data / restraints / parameters**	4570 / 0 / 334	4544 / 0 / 362	4680 / 0 / 353	9593 / 0 / 743	3521 / 0 / 289	3637 / 0 / 289	3713 / 0 / 298	5302 / 0 / 382
**Goodness-of-fit on F^2**	0.943	1.044	1.028	1.035	1.055	0.982	1.057	1.068
**Final R indices [I>2σ(I)]**	R_1 = 0.0556<br>wR_2 = 0.1328	R_1 = 0.0411<br>wR_2 = 0.1046	R_1 = 0.0659<br>wR_2 = 0.1372	R_1 = 0.0479<br>wR_2 = 0.1106	R_1 = 0.0441<br>wR_2 = 0.1019	R_1 = 0.0757<br>wR_2 = 0.2001	R_1 = 0.0443<br>wR_2 = 0.1184	R_1 = 0.0640<br>wR_2 = 0.1838
**R indices (all data)**	R_1 = 0.0943<br>wR_2 = 0.1485	R_1 = 0.0554<br>wR_2 = 0.1120	R_1 = 0.1161<br>wR_2 = 0.1581	R_1 = 0.0783<br>wR_2 = 0.1232	R_1 = 0.0673<br>wR_2 = 0.1109	R_1 = 0.1318<br>wR_2 = 0.2510	R_1 = 0.0533<br>wR_2 = 0.1241	R_1 = 0.0823<br>wR_2 = 0.1951
**Largest diff. peak and hole [e Å^{-3}]**	0.56 and -0.50	0.47 and -0.45	0.34 and -0.42	0.52 and -0.38	0.51 and -0.37	0.71 and -0.58	0.92 and -0.56	0.87 and -0.73
**CCDC number**	2295797	2295798	2295799	2295800	2295801	2296785	2295802	2295803

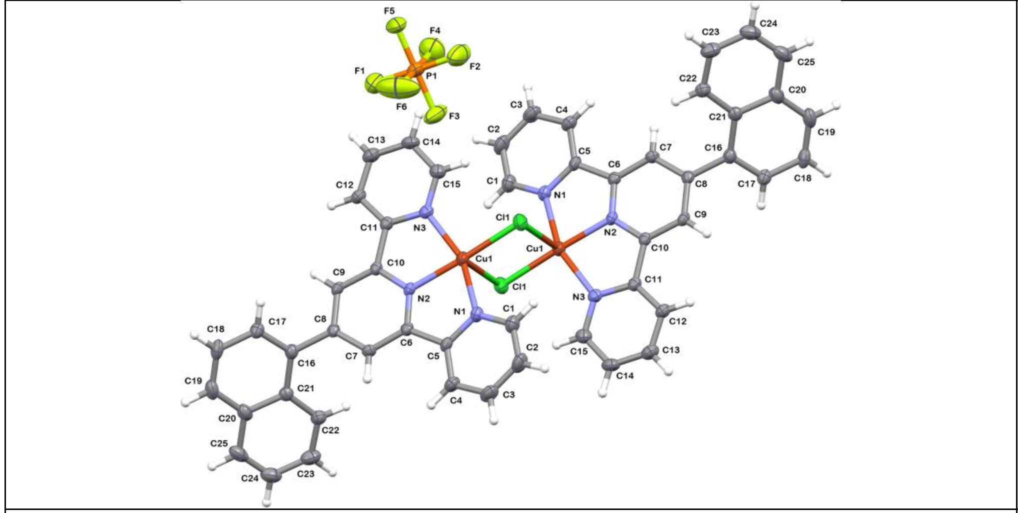	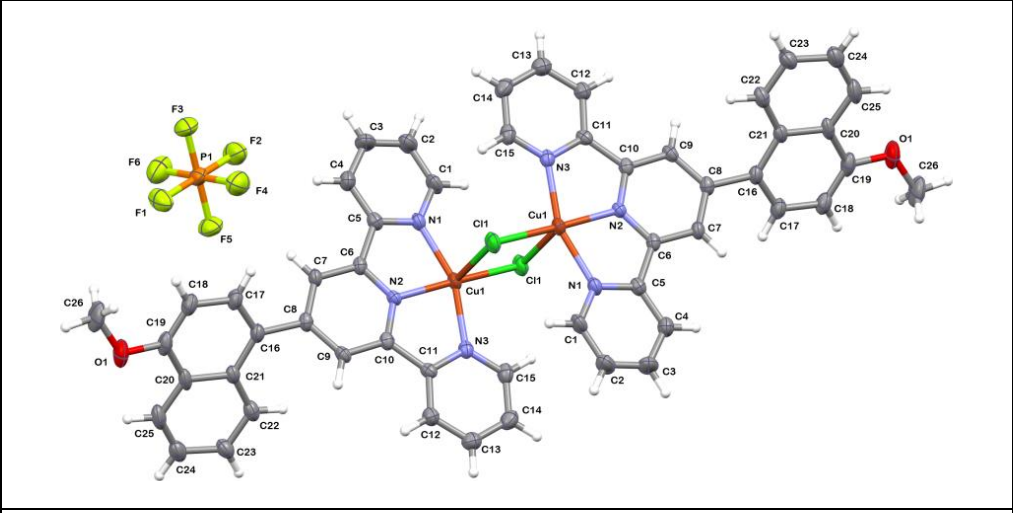
**1**	**3**
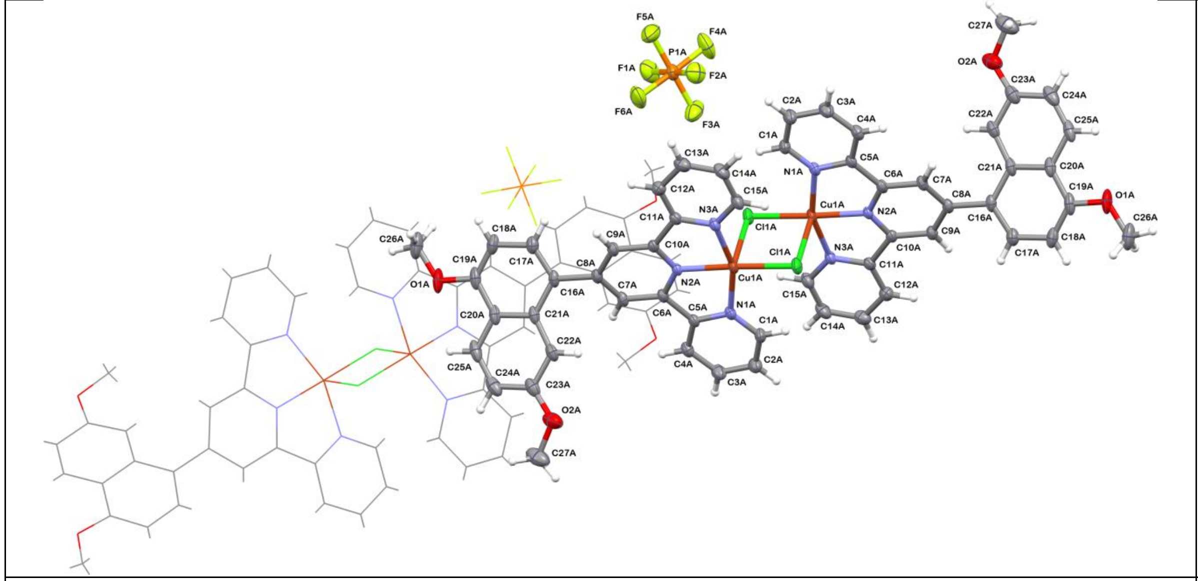	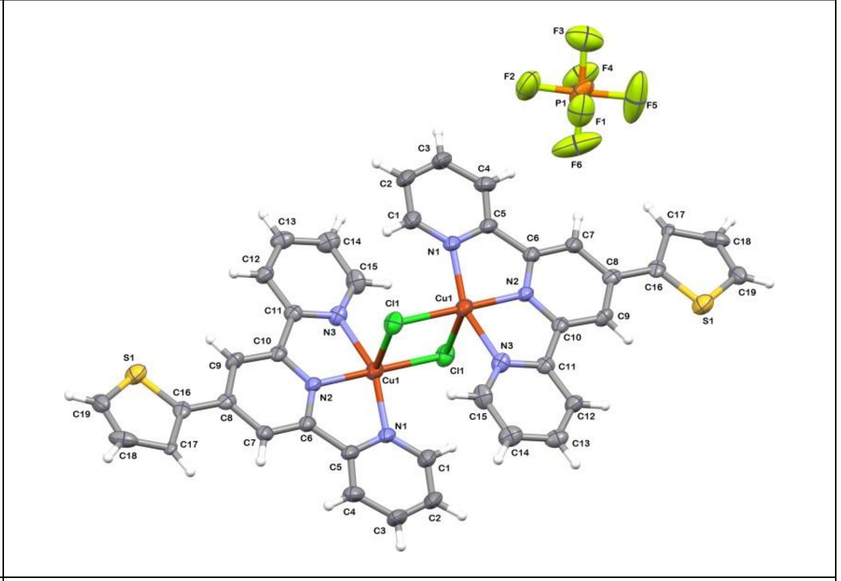
**4** (numeration and ellipsoids of molecule B omitted for clarity)	**6**

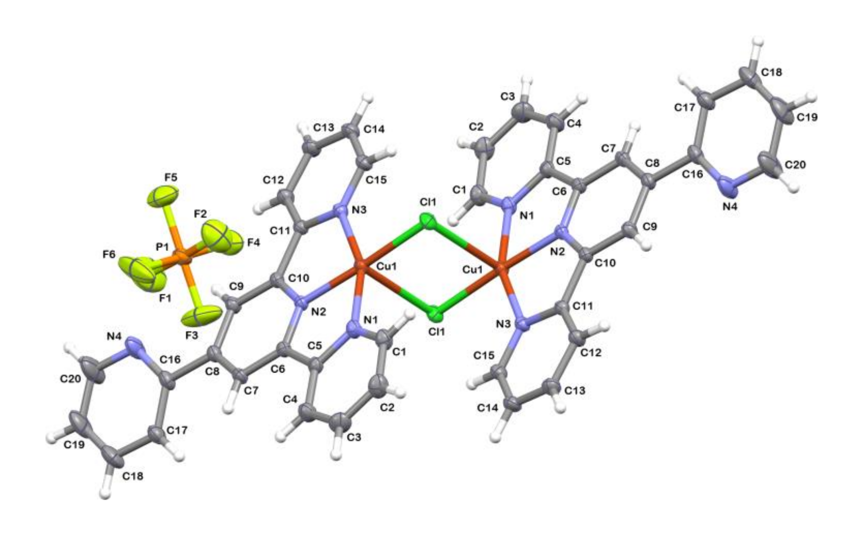

7

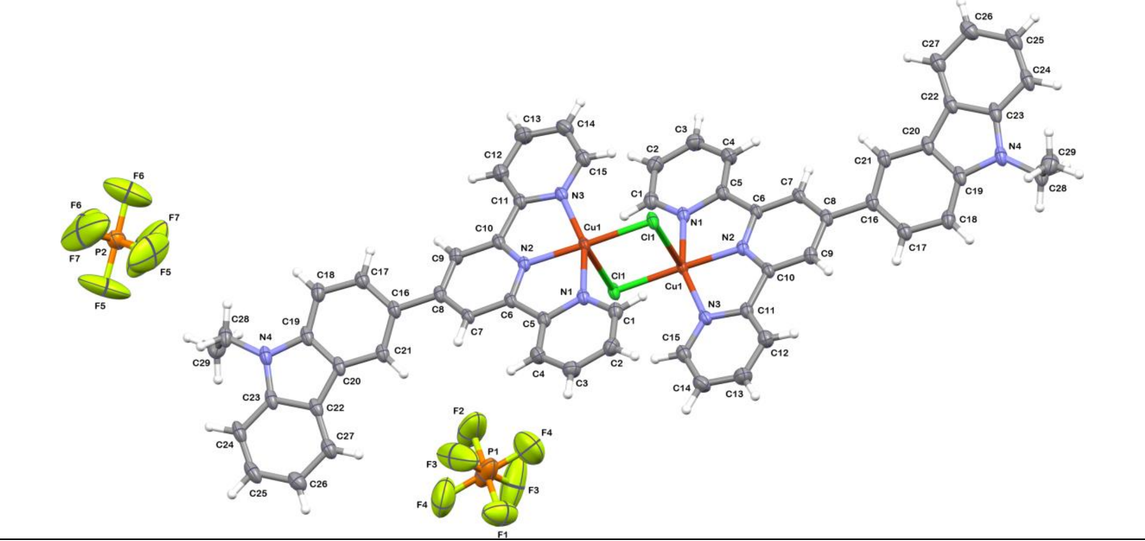

8

Figure S3. Dimer units of compounds **1, 3, 4, 6, 7** and **8**.

Table S2. The selected structural data of compounds **1-8**

parameter	1	2	3	4 molecule A	4 molecule B	5	6	7	8
τ_5	0.240	0.297	0.243	0.285	0.271	0.322	0.348	0.305	0.317
Cu•••Cu distance in dimer	3.5602(7)	3.5186(7)	3.5979(8)	3.6864(7)	3.5753(7)	3.4920(7)	3.499(1)	3.5207(5)	3.5590(8)
dihedral angle $py_{central}$–$py_{distal(1)}$	9.17	2.85	10.14	9.26	5.46	1.24	2.22	4.15	2.58
dihedral angle $py_{central}$–$py_{distal(2)}$	6.28	8.21	6.23	7.08	8.72	1.66	1.23	5.88	4.06
dihedral angle $py_{distal(1)}$–$py_{distal(2)}$	5.10	5.36	4.97	4.11	3.43	2.12	1.53	2.15	6.36
dihedral angle $py_{central}$–R	50.84	55.54	45.99	43.19	44.06	5.17	8.51	22.27	22.54
Cu–Cl–Cu angle	89.97(4)	88.87(3)	90.47(5)	91.52(4)	89.39(4)	87.13(3)	87.15(7)	89.54(3)	90.95(4)
Cl–Cu–Cl angle	90.03(4)	91.13(3)	89.53(5)	88.48(4)	90.61(4)	92.87(4)	92.85(7)	90.46(3)	89.05(4)
Cu distance from N Cl square pyramid base	0.145	0.103	0.125	0.090	0.109	0.067	0.063	0.106	0.089

Table S3. Selected bond lengths [Å] and angles [°] of compounds **1-8**

Bond lengths	1	2	3	4 molecule A	4 molecule B	5	6	7	8
Cu(1)–Cl(1)$_{(basal)}$	2.2243(12)	2.2064(8)	2.2179(14)	2.2047(10)	2.2097(10)	2.2083(9)	2.209(2)	2.2195(9)	2.2192(11)
Cu(1)–Cl(1)$_{(apical)}$	2.781(1)	2.7849(9)	2.815(2)	2.896(1)	2.834(1)	2.818(1)	2.825(2)	2.7510(10)	2.7458(12)
Cu(1)–N(1)	2.028(4)	2.023(2)	2.034(4)	2.027(3)	2.026(3)	2.015(3)	2.024(6)	2.019(3)	2.023(4)
Cu(1)–N(2)	1.939(3)	1.935(2)	1.941(4)	1.930(3)	1.924(3)	1.919(3)	1.920(6)	1.928(3)	1.935(3)
Cu(1)–N(3)	2.024(4)	2.019(2)	2.039(4)	2.026(3)	2.018(3)	2.008(3)	2.021(6)	2.020(3)	2.035(4)
**Bond angles**	**1**	**2**	**3**	**4 molecule A**	**4 molecule B**	**5**	**6**	**7**	**8**
Cl(1)$_{(apical)}$–Cu(1)–Cl(1)$_{(basal)}$	90.03(4)	91.13(3)	89.53(5)	88.48(4)	90.61(4)	92.87(4)	92.85(7)	90.47(3)	89.05(4)
Cu(1)–Cl(1)–Cu(1)	89.97(4)	88.87(3)	90.47(5)	91.52(4)	89.39(4)	87.13(3)	87.15(7)	89.53(3)	90.95(4)
N(1)–Cu(1)–Cl(1)$_{(basal)}$	99.19(10)	99.39(7)	99.47(12)	99.84(9)	100.18(9)	99.50(9)	99.8(2)	100.23(8)	100.26(10)
N(1)–Cu(1)–Cl(1)$_{(apical)}$	91.6(1)	99.41(7)	91.9(1)	88.95(9)	99.22(9)	90.15(9)	93.4(2)	97.06(9)	96.06(10)
N(2)–Cu(1)–Cl(1)$_{(basal)}$	172.87(10)	176.60(7)	173.23(12)	176.02(9)	175.22(9)	179.07(9)	179.66(16)	176.89(9)	177.81(10)
N(2)–Cu(1)–Cl(1)$_{(apical)}$	97.03(9)	92.27(7)	97.2(1)	95.49(8)	94.12(8)	88.04(8)	87.4(1)	92.57(8)	93.09(10)
N(3)–Cu(1)–Cl(1)$_{(basal)}$	99.69(11)	99.90(7)	100.20(12)	100.11(9)	99.08(9)	99.53(9)	100.2(2)	98.98(8)	99.25(10)
N(3)–Cu(1)–Cl(1)$_{(apical)}$	98.6(1)	88.98(7)	96.5(1)	98.40(9)	88.88(9)	95.84(9)	92.6(2)	92.19(8)	92.56(10)
N(1)–Cu(1)–N(2)	79.67(14)	80.07(9)	80.03(16)	80.03(12)	79.78(11)	80.33(12)	80.0(3)	80.08(11)	79.96(15)
N(1)–Cu(1)–N(3)	158.47(14)	158.78(10)	158.63(16)	158.89(12)	158.98(12)	159.72(11)	158.8(3)	158.57(11)	158.76(13)
N(2)–Cu(1)–N(3)	80.31(14)	80.15(9)	79.45(16)	79.59(12)	80.32(11)	80.54(12)	80.0(2)	80.22(11)	80.23(15)

Table S4. Short intra- and intermolecular hydrogen bonds detected in complexes **1-8**

D–H•••A	D–H [Å]	H•••A [Å]	D–A [Å]	D–H•••A [°]
**1**				
C(2)–H(2)•••F(3)[a]	0.93	2.44	3.326(7)	159.00
**2**				
C(1)–H(1)•••Cl(1)	0.93	2.83	3.379(4)	119.00
C(4)–H(4)•••F(3)[b]	0.93	2.54	3.381(4)	151.00
C(12)–H(12)•••F(3)[c]	0.93	2.55	3.451(5)	164.00
C(16)–H(16)••Cl(1)[d]	0.93	2.79	3.553(3)	140.00
C(24)–H(24)•••N(5)[c]	0.93	2.60	3.502(8)	163.00
C(26)–H(26A)•••F(4)	0.93	2.54	3.208(11)	127.00
C(26)–H(26B)•••F(2)[e]	0.93	2.48	3.334(11)	148.00
**3**				
C(12)–H(12)•••F(5)[f]	0.93	2.48	3.362(6)	158.00
C(25)–H(25)•••O(1)	0.93	2.36	2.694(8)	101.00
C(26)–H(26C)•••F(3)[g]	0.93	2.50	3.365(8)	150.00
**4**				
C(3A)–H(3A)•••F(4A)[h]	0.93	2.50	3.252(6)	138.00
C(4B)–H(4B)•••F(5A)[h]	0.93	2.41	3.321(5)	166.00
C(9A)–H(9A)•••F(3B)	0.93	2.52	3.358(5)	150.00
C(9B)–H(9B)••F(3B)[i]	0.93	2.33	3.255(4)	173.00
C(14B)–H(14B)•••F(5B)[j]	0.93	2.52	3.405(6)	160.00
C(15B)–H(15B)•••Cl(1B)	0.93	2.82	3.371(4)	119.00
C(25A)–H(25A)•••O(1A)	0.93	2.43	2.744(6)	100.00
C(25B)–H(25B)•••O(1B)	0.93	2.40	2.722(5)	100.00
C(26B)–H(26F)•••F(3A)	0.96	2.46	3.167(5)	130.00
**5**				
C(1)–H(1)•••Cl(1)	0.93	2.82	3.373(4)	119.00
C(1)–H(1)•••Cl(1)[k]	0.93	2.71	3.512(4)	145.00
C(7)–H(7)•••O(1)	0.93	2.46	2.767(4)	100.00
C(9)–H(9)•••F(5)[l]	0.93	2.52	3.443(5)	171.00
C(15)–H(15)•••Cl(1)	0.93	2.81	3.360(5)	119.00
C(17)–H(17)•••F(5)[l]	0.93	2.55	3.450(5)	163.00
**6**				
C(1)–H(1)•••Cl(1)	0.93	2.82	3.372(10)	119.0
C(9)–H(9)•••S(1)	0.93	2.61	3.046(8)	106.0
C(15)–H(15)•••Cl(1)	0.93	2.79	3.358(8)	120.0
C(15)–H(15)•••Cl(1)[j]	0.93	2.73	3.469(8)	137.0
**7**				
C(14)–H(14)•••F(6)[m]	0.93	2.40	3.145(6)	137.00
C(15)–H(15)•••Cl(1)	0.93	2.82	3.373(4)	119.00
**8**				
C(3)–H(3)•••F(4)[n]	0.93	2.50	3.386(13)	159.00
C(12)–H(12)•••F(3)[h]	0.93	2.46	3.223(14)	139.00
C(17)–H(17)••F(1)[h]	0.93	2.51	3.368(8)	153.00
C(24)–H(24)••Cl(1)[o]	0.93	2.75	3.521(5)	140.00

symmetry codes: (a) = 1-x,2-y,1-z; (b) = 3/2-x,1/2+y,1/2-z; (c) = -1+x,y,z; (d) = -1/2+x,1/2-y,-1/2+z; (e) = x,1+y,z; (f) = 1/2-x,1/2+y,1/2-z; (g) = -1/2-x,1/2+y,1/2-z; (h) = x,-1+y,z; (i) = 1+x,y,z; (j) = 1-x,1-y,-z; (k) = 2-x,1-y,1-z; (l) = -1+x,3/2-y,1/2+z; (m) = -1+x,1/2-y,-1/2+z; (n) = 3/2-x,y,1/2-z; (o) = x,y,-1+z.

Table S5. Short π•••π interactions for complexes **1-8**

Cg(I)•••Cg(J)	Cg(I)•••Cg(J) [Å]	α [°]	β [°]	γ [°]	Cg(I)-Perp [Å]	Cg(J)-Perp [Å]
**1**						
Cg(1)•••Cg(2)[p]	3.719(2)	5.05(19)	20.0	15.7	3.5792(15)	3.4947(18)
Cg(1)•••Cg(3)[p]	3.814(3)	4.3(2)	20.6	20.2	3.5796(15)	3.569(2)
**2**						
Cg(1)•••Cg(4)[q]	3.6809(17)	8.28(14)	18.7	13.5	3.5797(11)	3.4858(13)
Cg(1)•••Cg(5)[q]	3.8457(19)	7.74(16)	23.5	21.2	3.5860(11)	3.5256(15)
**3**						
Cg(1)•••Cg(2)[q]	3.853(3)	13.6(2)	3.1	16.6	3.6930(19)	3.848(2)
Cg(1)•••Cg(3)[f]	3.641(3)	8.6(3)	21.9	14.4	3.5264(19)	3.378(2)
**4**						
Cg(1A)•••Cg(3B)[c]	3.757(2)	6.40(17)	22.8	16.7	3.5988(13)	3.4626(15)
Cg(3A)•••Cg(1B)[c]	3.790(2)	4.07(19)	27.6	24.6	3.4445(19)	3.3595(13)
**5**						
Cg(6)•••Cg(6)[r]	3.513(2)	0.0(2)	18.4	18.4	3.3328(16)	3.3326(16)
Cg(6)•••Cg(7)[c]	3.976(2)	4.8(2)	30.5	32.8	3.3414(16)	3.4267(15)
Cg(7)•••Cg(1)[i]	3.866(2)	1.24(17)	28.2	27.2	3.4383(15)	3.4067(13)
**6**						
Cg(8)•••Cg(8)[s]	3.626(4)	0.0(4)	17.3	17.3	3.462(3)	3.462(3)
Cg(1)•••Cg(9)[c]	3.906(4)	1.2(3)	26.0	27.2	3.473(3)	3.510(3)
7						
Cg(1)•••Cg(9)[i]	3.817(2)	5.89(16)	21.0	25.0	3.4593(13)	3.5623(14)
**8**						
Cg(7)•••Cg(10)[n]	3.802(3)	6.6(3)	31.6	25.9	3.419(2)	3.238(2)
Cg(1)•••Cg(2)[n]	3.678(3)	3.4(2)	20.7	18.0	3.4981(19)	3.441(2)
Cg(1)•••Cg(10)[j]	3.978(3)	22.6(2)	35.8	13.5	3.8673(19)	3.228(2)

symmetry codes: (p) = 3/2-x,1/2+y,3/2-z; (q) = 1/2-x,-1/2+y,1/2-z; (f) = 1/2-x,1/2+y,1/2-z; (c) = -1+x,y,z; (r) -x,1-y,2-z; (i) = 1+x,y,z; (s) = -1-x,1-y,1-z; (n) = 3/2-x,y,1/2-z; (j) = 1-x,1-y,-z.

α = dihedral angle between *Cg*(I) and *Cg*(J); *Cg*(I)-Perp = Perpendicular distance of *Cg*(I) on ring J; *Cg*(J)-Perp = perpendicular distance of *Cg*(J) on ring I; β = angle *Cg*(I)→*Cg*(J) vector and normal to ring I; γ = angle *Cg*(I) →*Cg*(J) vector and normal to plane J;

Cg(1) is the centroid of atoms = N2/C6/C7/C8/C9/C10; Cg(2) is the centroid of atoms = C16/C17/C18/C19/C20/C21; Cg(3) is the centroid of atoms = C20/C21/C22/C23/C24/C25; Cg(4) is the centroid of atoms = N4/C16/C17/C18/C19/C20; Cg(5) is the centroid of atoms = C19/C20/C21/C22/C23/C24; Cg(6) is the centroid of atoms = O1/C16/C17/C18/C19; Cg(7) is the centroid of atoms = N1/C1/C2/C3/C4/C5; Cg(8) is the centroid of atoms = S1/C16/C17/C18/C19; Cg(9) is the centroid of atoms = N3/C11/C12/C13/C14/C15; Cg(10) is the centroid of atoms = C22/C23/C24/C25/C26/C27.

Table S6. X–Y•••Cg(J)(π-ring) interactions for complexes **1-8**

X–Y•••Cg(J)	X(I)•••Cg(J) [Å]	X-Perp [Å]	γ [°]	Y-X(I)•••Cg(J) [°]
**1**				
no interactions detected				
**2**				
P(1)–F(1) ••• Cg(5)^{i}	3.966(4)	-3.647	23.11	126.96(14)
**3**				
C(26)–H(26B) ••• Cg(9)^{f}	2.85	-2.80	10.71	135.0(1)
**4**				
P(1A)-F(3A) ••• Cg(9A)^{t}	3.605(4)	3.469	15.79	122.31(19)
**5**				
P(1)-F(1) ••• Cg(9)^{u}	3.883(5)	3.388	29.26	107.6(2)
P(1)-F(3) ••• Cg(7)^{v}	3.588(4)	-3.272	24.25	122.59(17)
P(1)-F(4) ••• Cg(9)^{u}	3.788(4)	3.665	14.65	112.15(19)
**6**				
P(1)-F(3) ••• Cg(9)^{w}	3.545(8)	3.165	26.78	125.0(4)
P(1)-F(4) ••• Cg(7)^{c}	3.576(10)	-3.246	24.80	121.7(4)
**7**				
P(1)-F(1) ••• Cg(1)^{t}	3.670(6)	3.598	11.38	97.7(2)
P(1)-F(4) ••• Cg(1)^{t}	3.187(4)	2.886	25.06	118.21(18)
P(1)-F(5) ••• Cg(9)^{x}	3.741(4)	-3.287	28.51	109.67(17)
**8**				
no interactions detected				

symmetry codes: (i) = 1+x,y,z; (f) = 1/2-x,1/2+y,1/2-z; (t) = x,y,z; (u) = x,3/2-y,-1/2+z; (v) = 1-x,1-y,1-z; (w) = -1-x,-1/2+y,1/2-z; (x) = x,1/2-y,1/2+z.
γ = angle X(I)→Cg(J) vector and normal to plane J.
Cg(5) is the centroid of atoms = C19/C20/C21/C22/C23/C24; Cg(8) is the centroid of atoms = N3/C11/C12/C13/C14/C15; Cg(7) is the centroid of atoms = N1/C1/C2/C3/C4/C5; Cg(1) is the centroid of atoms = N2/C6/C7/C8/C9/C10.

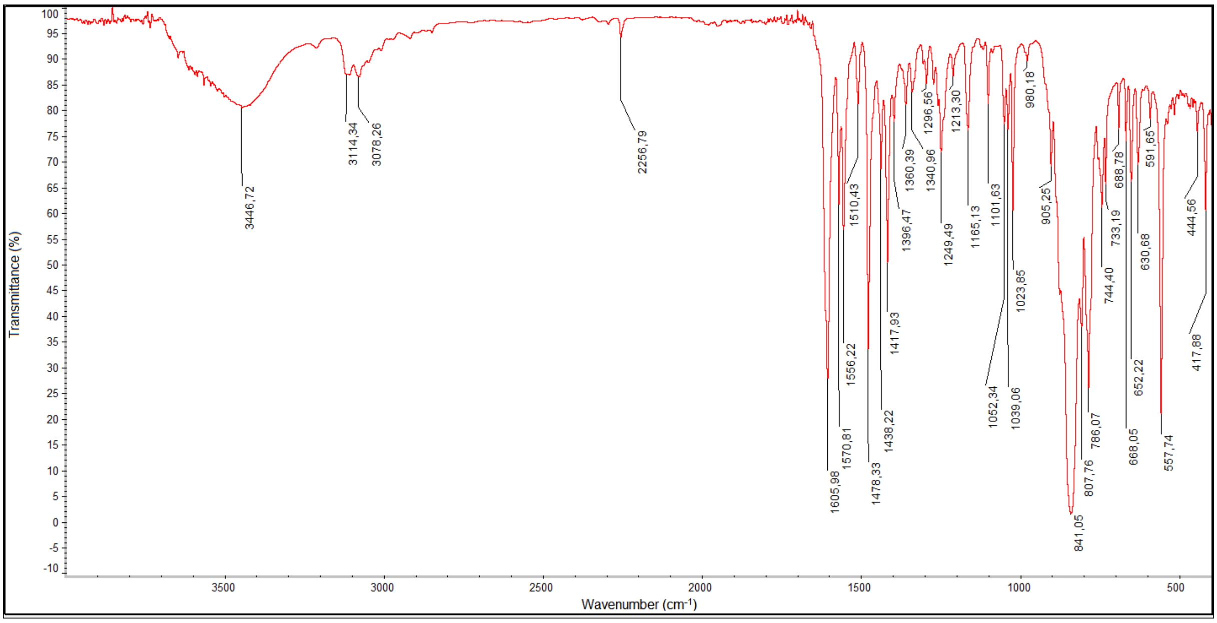
Transmittance (%)
Wavenumber (cm-1)
100
95
90
85
80
75
70
65
60
55
50
45
40
35
30
25
20
15
10
5
0
-5
-10
3500
3000
2500
2000
1500
1000
500
3446,72
3114,34
3078,26
2256,79
1605,98
1570,81
1556,22
1510,43
1478,33
1438,22
1417,93
1396,47
1360,39
1340,96
1296,56
1249,49
1213,30
1165,13
1101,63
1052,34
1039,06
1023,85
980,18
905,25
841,05
807,76
786,07
744,40
733,19
688,78
668,05
652,22
630,68
591,65
557,74
444,56
417,88

1

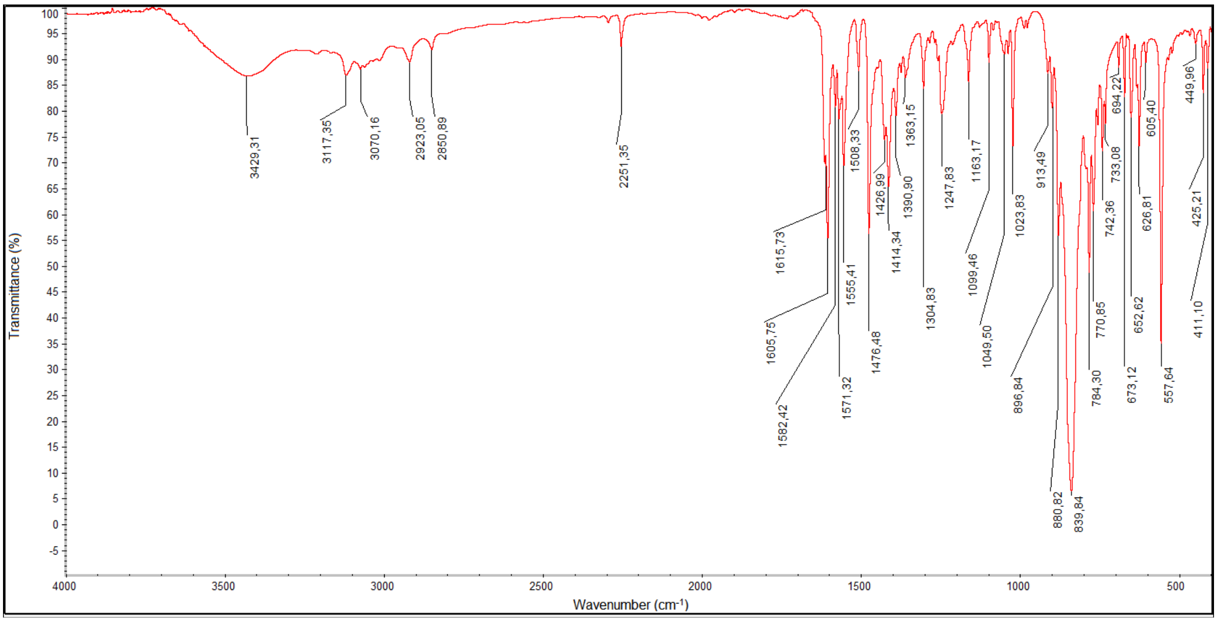
Transmittance (%)
Wavenumber (cm-1)
100
95
90
85
80
75
70
65
60
55
50
45
40
35
30
25
20
15
10
5
0
-5
4000
3500
3000
2500
2000
1500
1000
500
3429,31
3117,35
3070,16
2923,05
2850,89
2251,35
1615,73
1605,75
1582,42
1571,32
1555,41
1508,33
1476,48
1426,99
1414,34
1390,90
1363,15
1304,83
1247,83
1163,17
1099,46
1049,50
1023,83
913,49
896,84
880,82
839,84
784,30
770,85
742,36
733,08
694,22
673,12
652,62
626,81
605,40
557,64
449,96
425,21
411,10

2

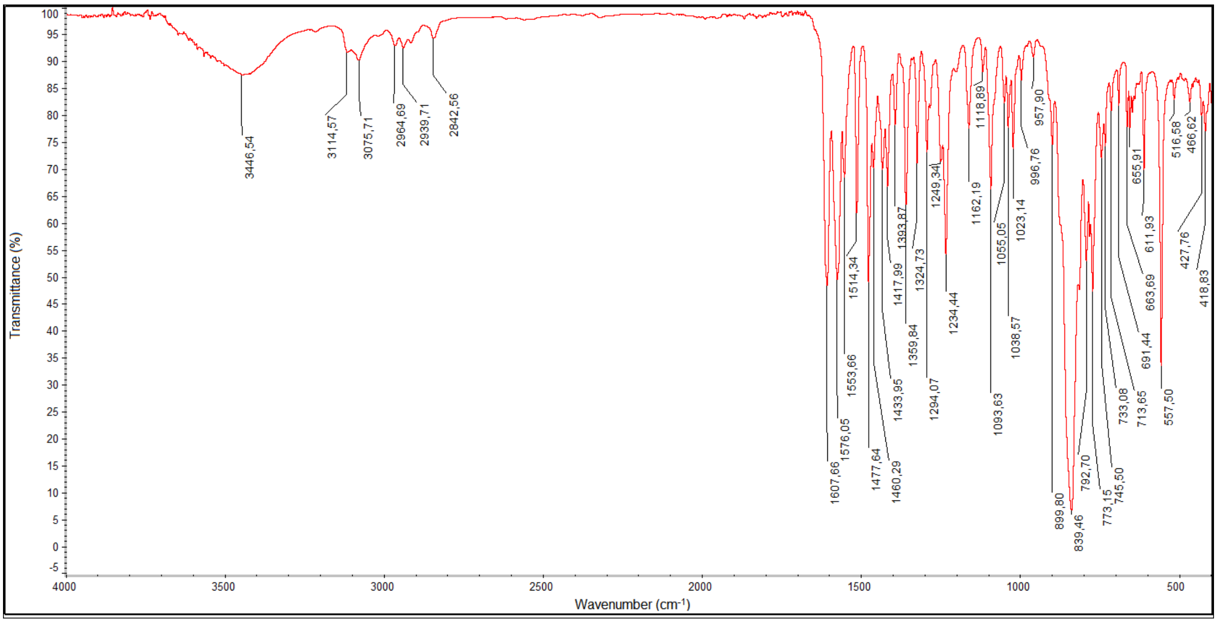
Transmittance (%)
Wavenumber (cm-1)
3446,54
3114,57
3075,71
2964,69
2939,71
2842,56
1607,66
1576,05
1553,66
1514,34
1477,64
1460,29
1433,95
1417,99
1393,87
1359,84
1324,73
1294,07
1249,34
1234,44
1162,19
1118,89
1093,63
1055,05
1038,57
1023,14
996,76
957,90
899,80
839,46
792,70
773,15
745,50
733,08
713,65
691,44
663,69
655,91
611,93
557,50
516,58
466,62
427,76
418,83

3

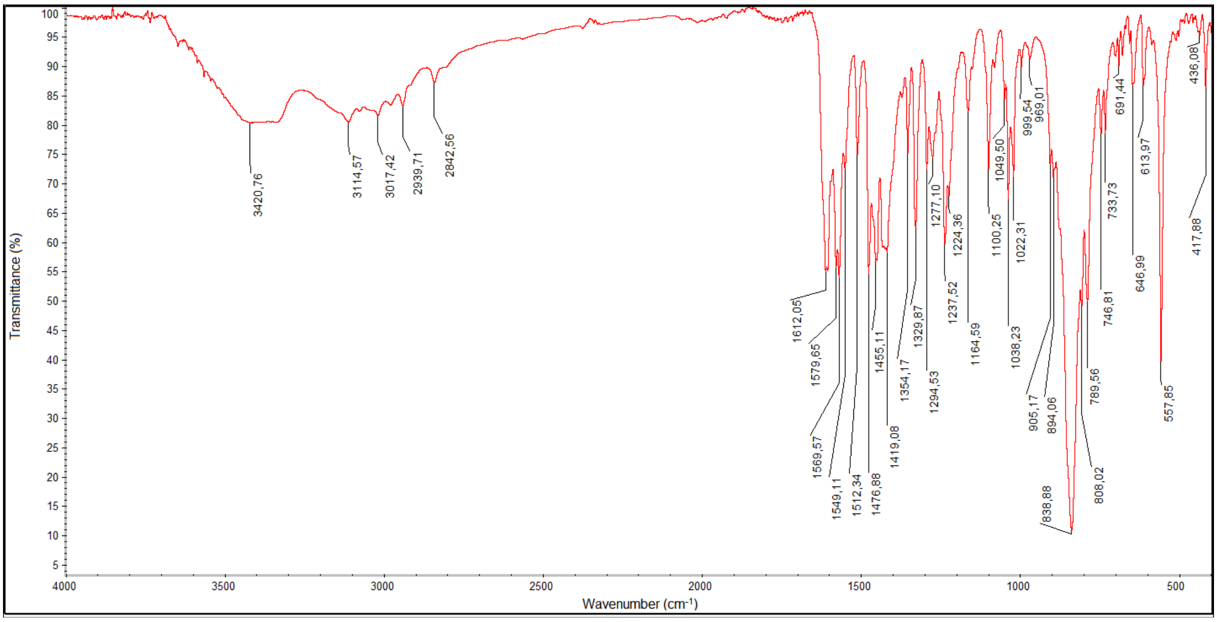
Transmittance (%)
Wavenumber (cm-1)
3420,76
3114,57
3017,42
2939,71
2842,56
1612,05
1579,65
1569,57
1549,11
1512,34
1476,88
1455,11
1419,08
1354,17
1329,87
1294,53
1277,10
1237,52
1224,36
1164,59
1100,25
1049,50
1038,23
1022,31
999,54
969,01
905,17
894,06
838,88
808,02
789,56
746,81
733,73
691,44
646,99
613,97
557,85
436,08
417,88

4

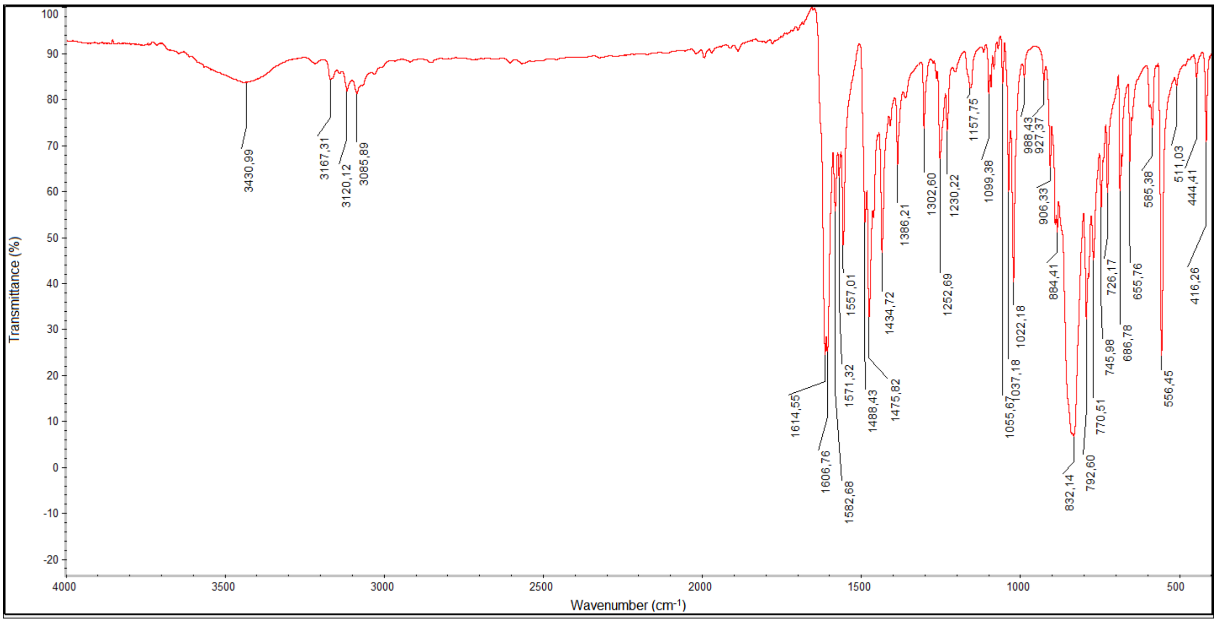

Transmittance (%)
Wavenumber (cm-1)
3430,99
3167,31
3120,12
3085,89
1614,55
1606,76
1582,68
1571,32
1557,01
1488,43
1475,82
1434,72
1386,21
1302,60
1252,69
1230,22
1157,75
1099,38
1055,67
1037,18
1022,18
988,43
927,37
906,33
884,41
832,14
792,60
770,51
745,98
726,17
686,78
655,76
585,38
556,45
511,03
444,41
416,26

5

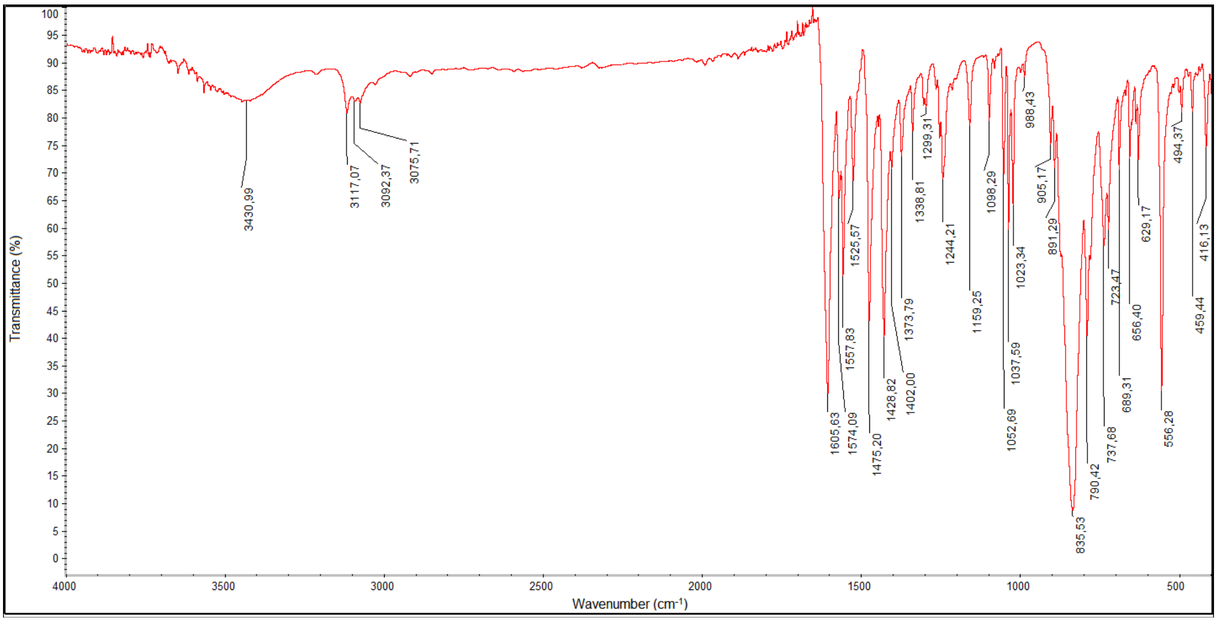

Transmittance (%)
Wavenumber (cm-1)
3430,99
3117,07
3092,37
3075,71
1605,63
1574,09
1557,83
1525,57
1475,20
1428,82
1402,00
1373,79
1338,81
1299,31
1244,21
1159,25
1098,29
1052,69
1037,59
1023,34
988,43
905,17
891,29
835,53
790,42
737,68
723,47
689,31
656,40
629,17
556,28
494,37
459,44
416,13

6

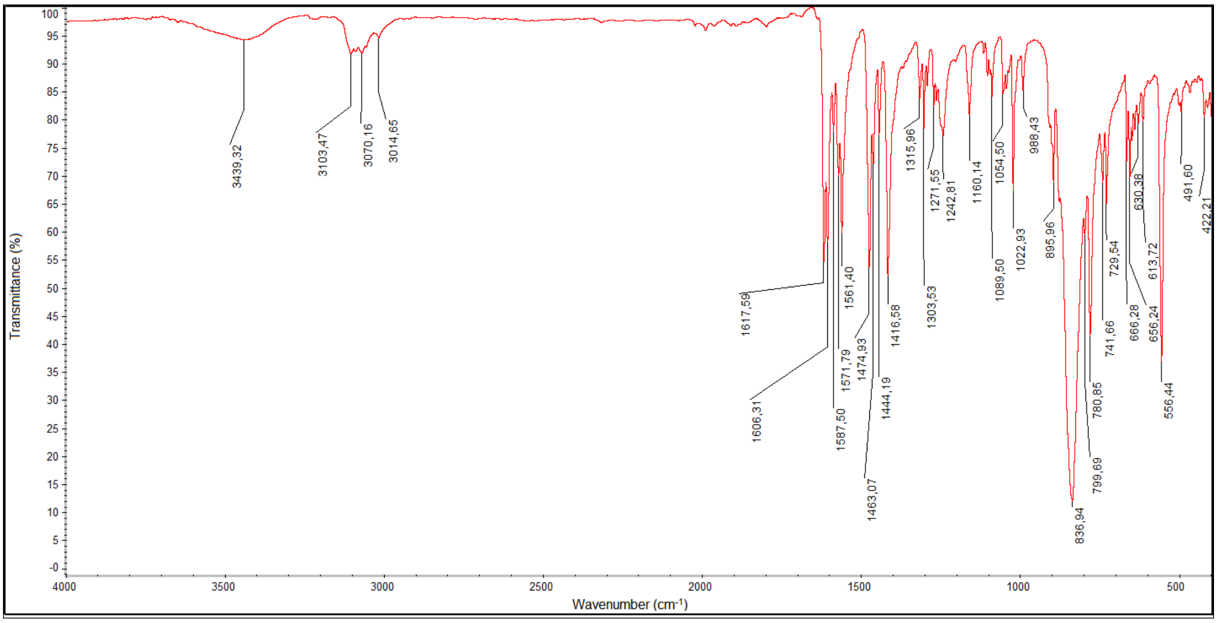

7

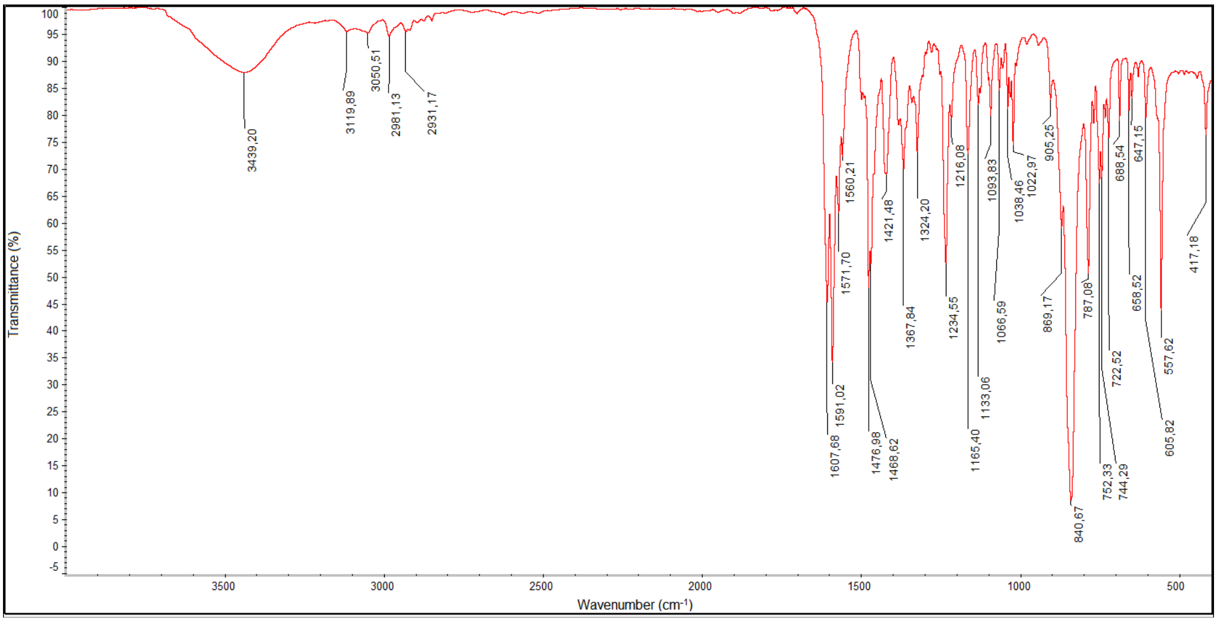

8

Figure S4. FT-IR spectra of the complexes **1-8**.

Table S7. Molar conductivity values Λ_M (S cm^2 mol^{-1}) of the complexes in DMSO at 25 °C.

**compound**	**Λ_M (S cm^2 mol^{-1})**
**1**	59.8 ± 1.2
**2**	58.2 ± 1.2
**3**	61.0 ± 1.2
**4**	71.2 ± 1.4
**5**	63.2 ± 1.3
**6**	53.6 ± 1.1
**7**	63.3 ± 1.3
**8**	60.2 ± 1.2

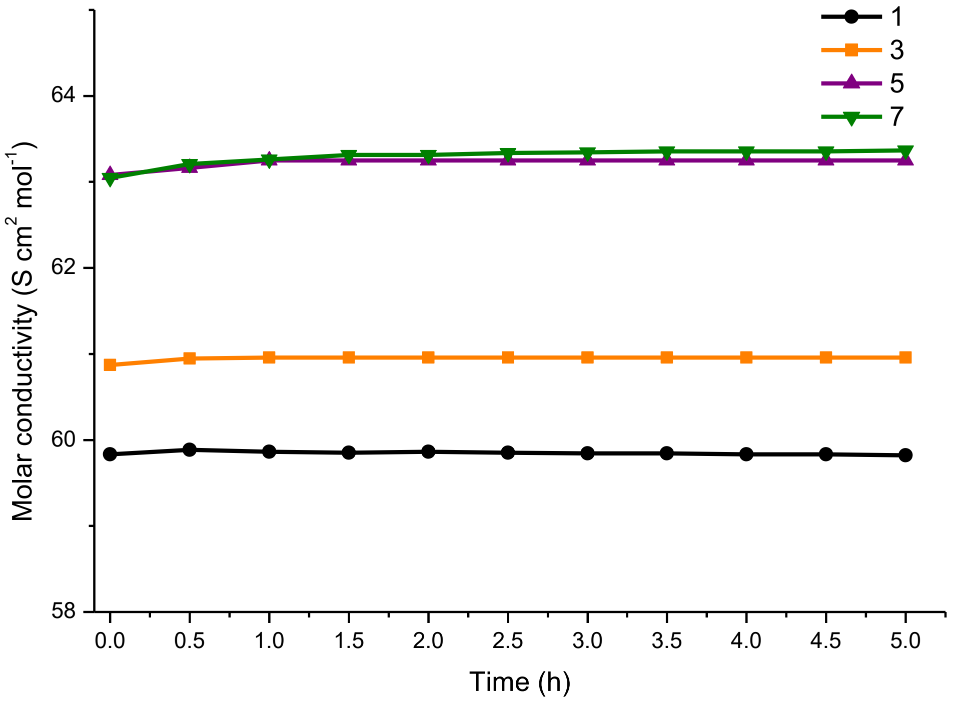

Figure S5. Plot of the molar conductivities Λ_M vs time for the representative Cu(II) samples **1**, **3**, **5**, and **7** at 25 °C.

Table S8. UV-Vis properties of Cu(II) compounds in DMSO and in solid.

	Wavelength/nm	
**compound**	**DMSO (ε × 10^4/$M^{-1}cm^{-1}$)**	**solid**
**1**	706 (0.02); 480 (0.2); 345 (2.8); 333 (2.8); 290 (4.4); 281 (4.0)	650, 399, 332
**2**	710 (0.02); 345 (2.4); 332 (2.7); 320 (2.3); 291 (3.9); 282 (3.7)	667, 423(sh), 337
**3**	706 (0.02); 382 (1.1); 340 (2.2); 326 (2.7); 316 (2.6); 290 (3.6)	675, 485(sh), 430, 368
**4**	709 (0.02); 392 (1.3); 328 (3.0); 290 (4.2); 278 (3.8); 267 (4.2)	659, 455, 395, 325
**5**	708 (0.02); 356 (4.0); 343 (4.0); 328 (3.0); 310 (2.9); 289 (3.7); 269 (3.2)	675, 409, 326, 287
**6**	708 (0.02); 354 (4.2); 343 (4.2); 290 (4.3); 271 (3.7)	655, 404, 333, 283
**7**	706 (0.02); 352 (1.9); 337 (2.2); 306 (3.1); 290 (6.2); 284 (5.9); 272 (4.6)	657, 422(sh), 381, 332
**8**	703 (0.02); 406 (3.5); 335 (4.0); 324 (4.3); 291 (8.4); 281 (7.9)	645, 487, 422, 377, 322

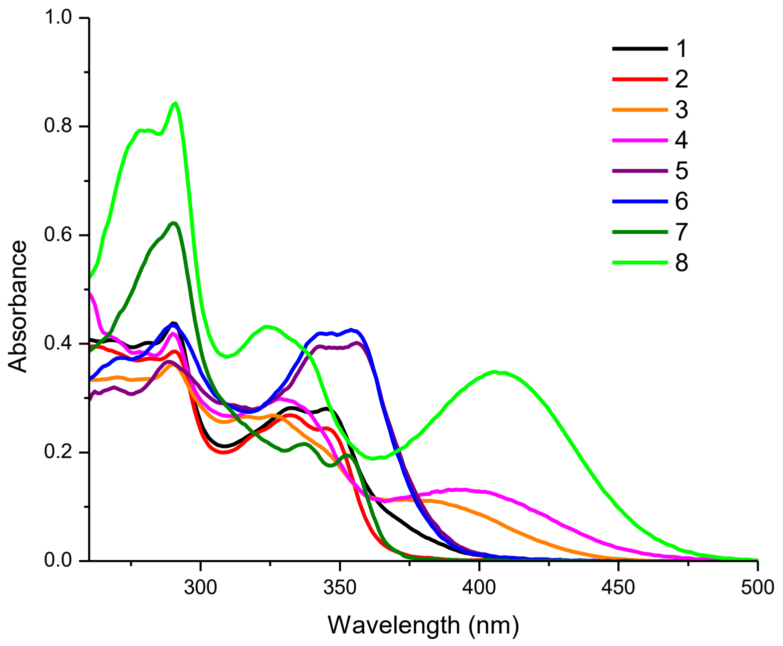

Figure S6. UV-Vis spectra of complexes **1**–**8** in DMSO.

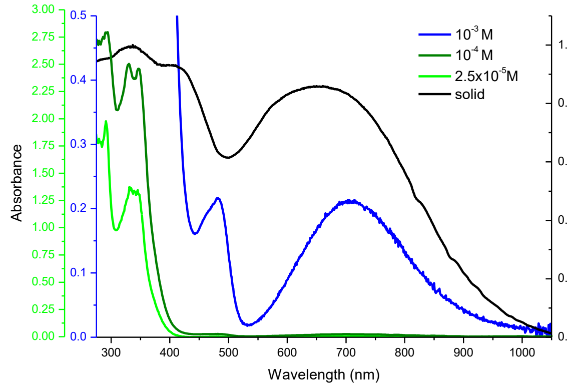

1

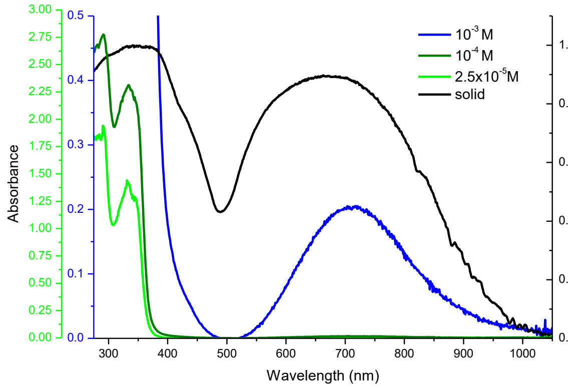

2

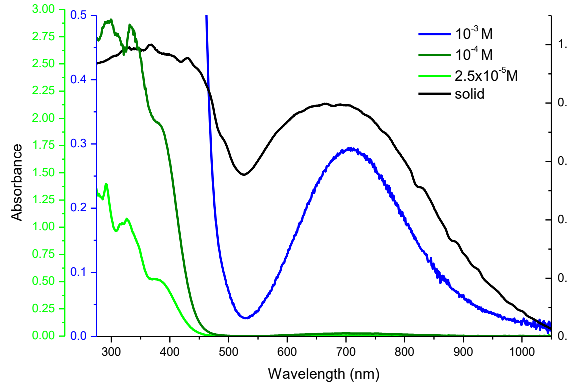

3

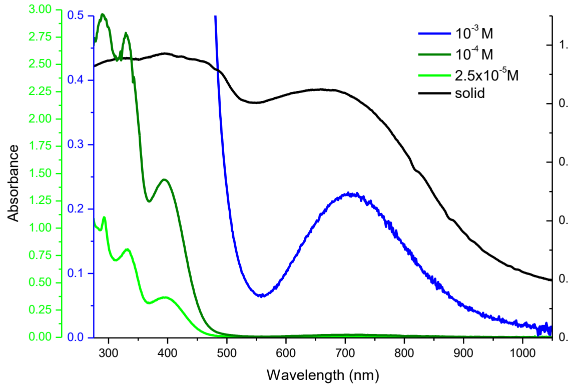

4

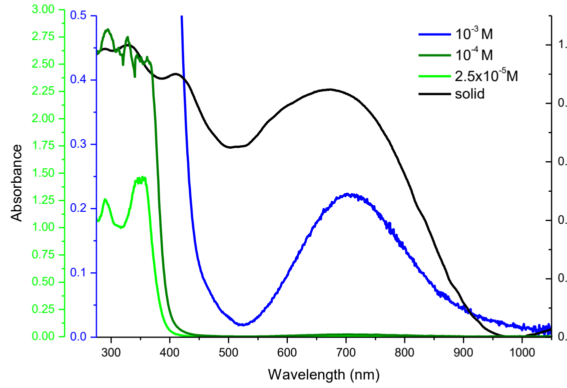

5

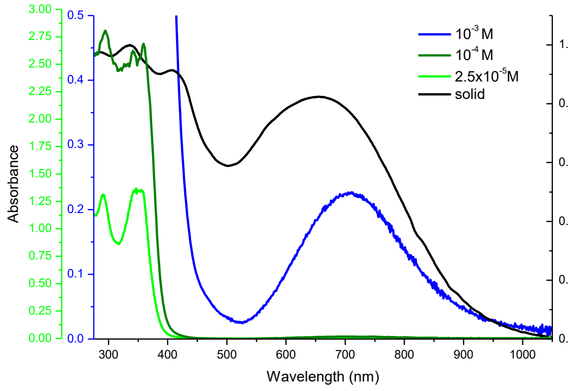

6

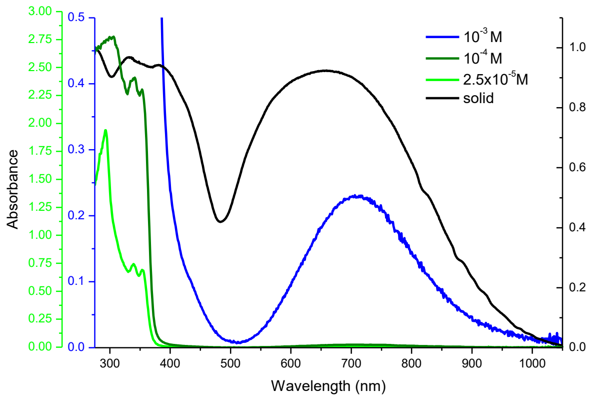

7

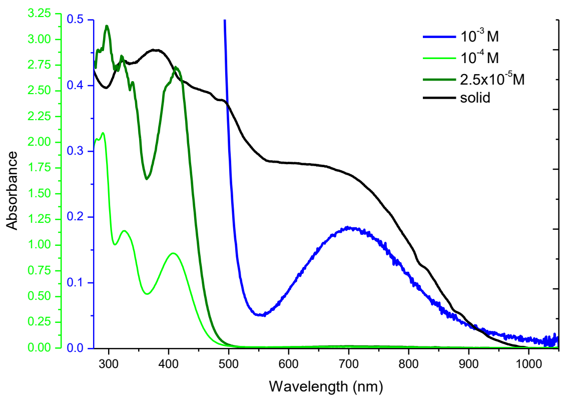

8

Figure S7. Comparison of the d–d range of the UV-Vis spectra in DMSO (concentration range 10^{-3}–2.5×10^{-5} M) and the respective diffuse reflectance spectra of powdered solids of **1-8**.

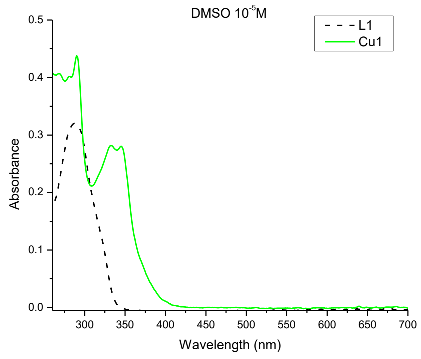

1

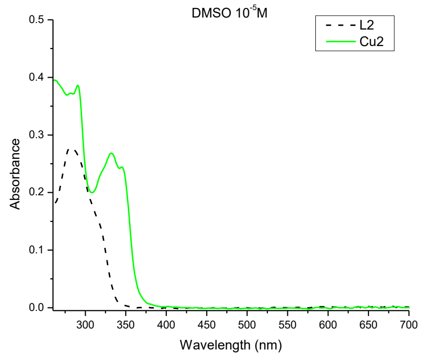

2

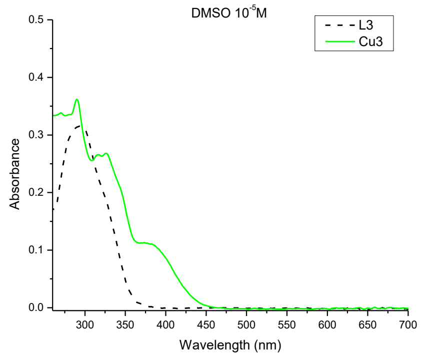

3

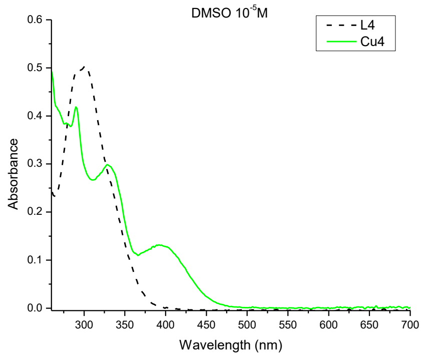

4

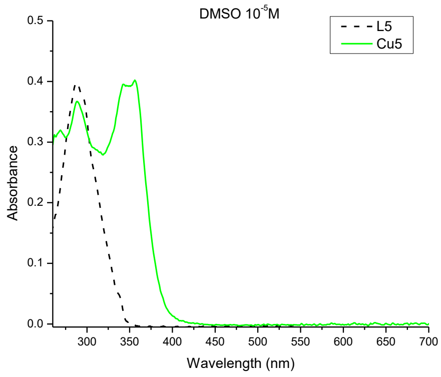

5

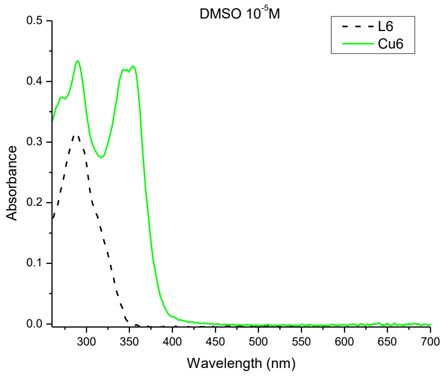

6

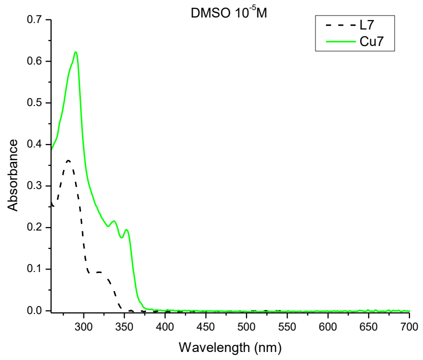

7

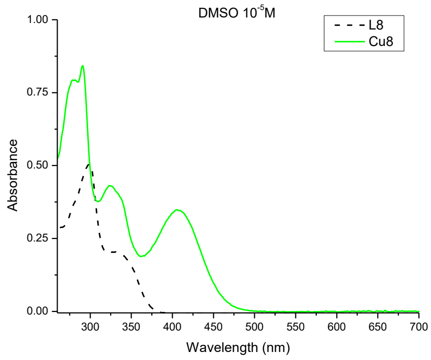

8

Figure S8. UV-Vis spectra of compounds **1-8** and respective ligands **L1-L8** in DMSO ($c = 10^{-5}$ M).

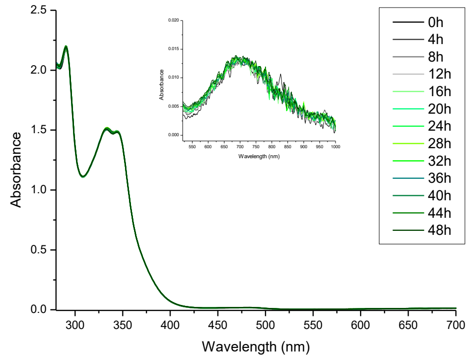

1

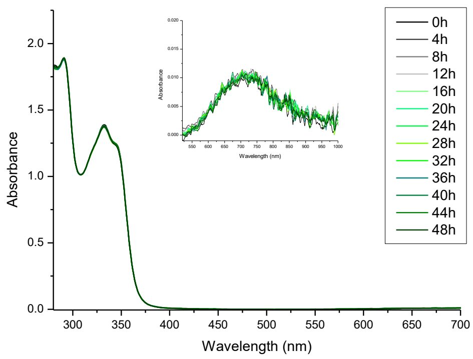

2

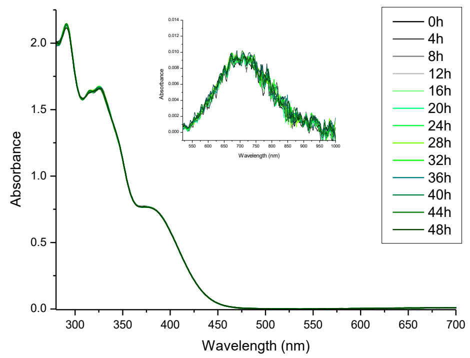

3

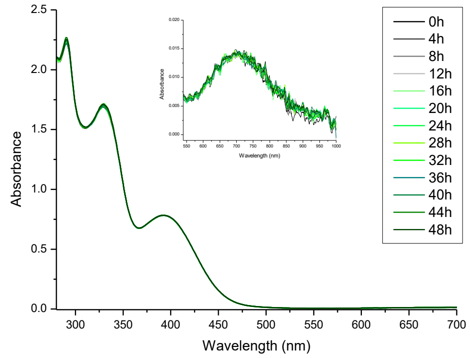

4

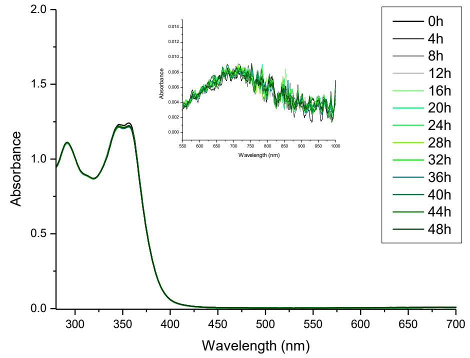

5

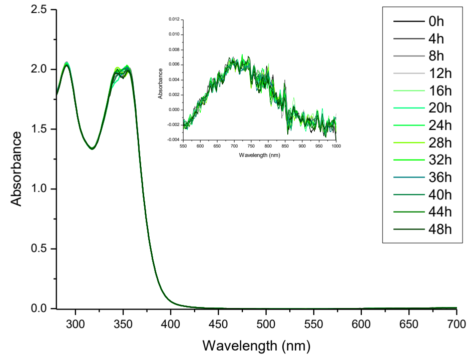

6

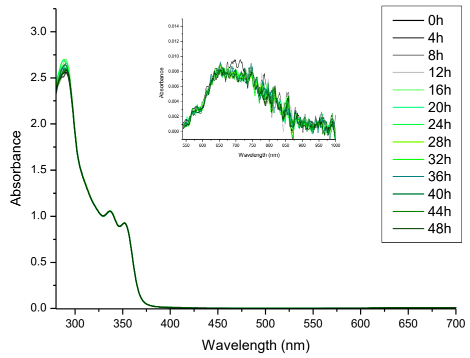

7

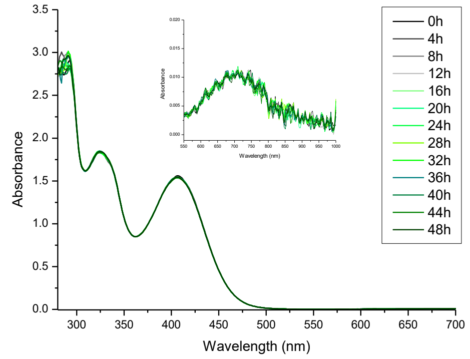

8

Figure S9. Kinetic UV-Vis spectra of compounds **1-8** in DMSO ($c = 5 \times 10^{-5}$ M). Spectra were recorded every 4h for 48h at RT. Insert: close-up of the d-d region of the spectra.

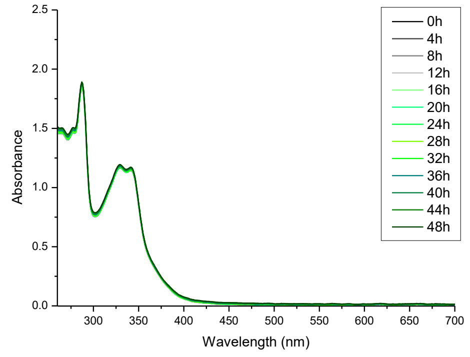

1

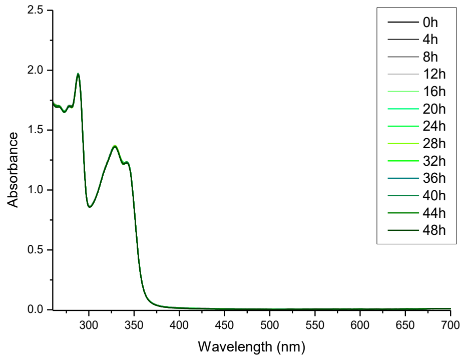

2

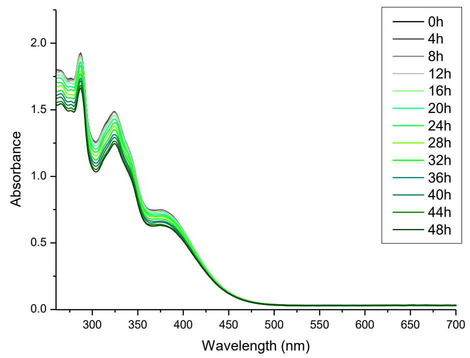

3

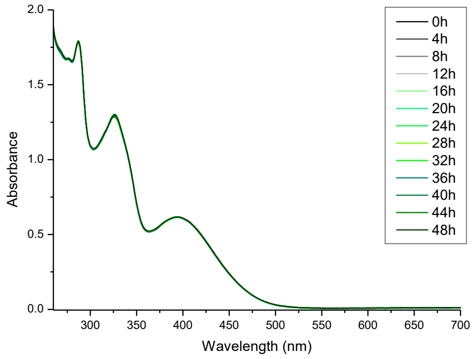

4

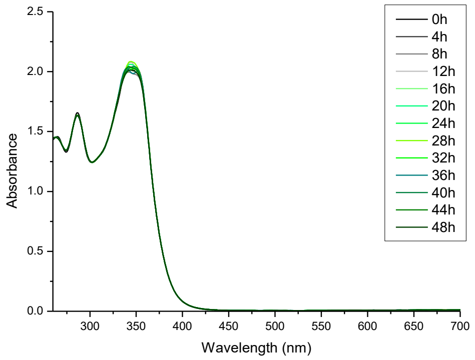

5

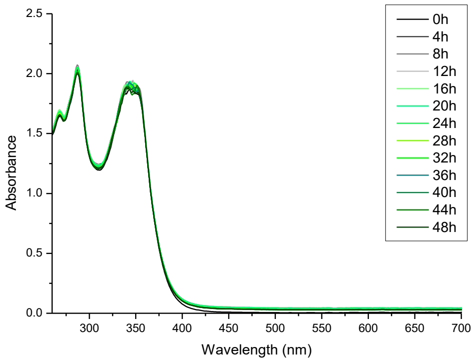

6

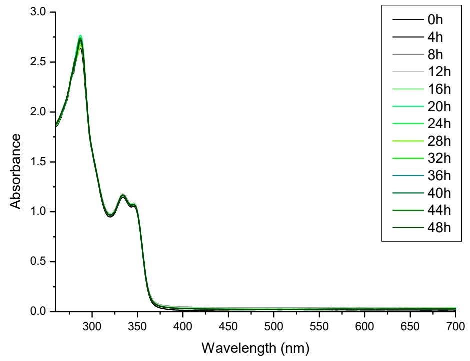

7

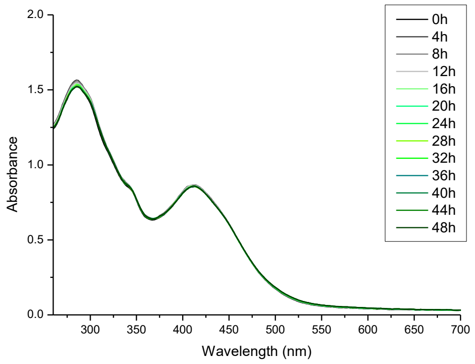

8

Figure S10. Kinetic UV-Vis spectra of compounds **1-8** in PBS ($c = 5 \times 10^{-5}$ M). Spectra were recorded every 4h for 48h at RT.

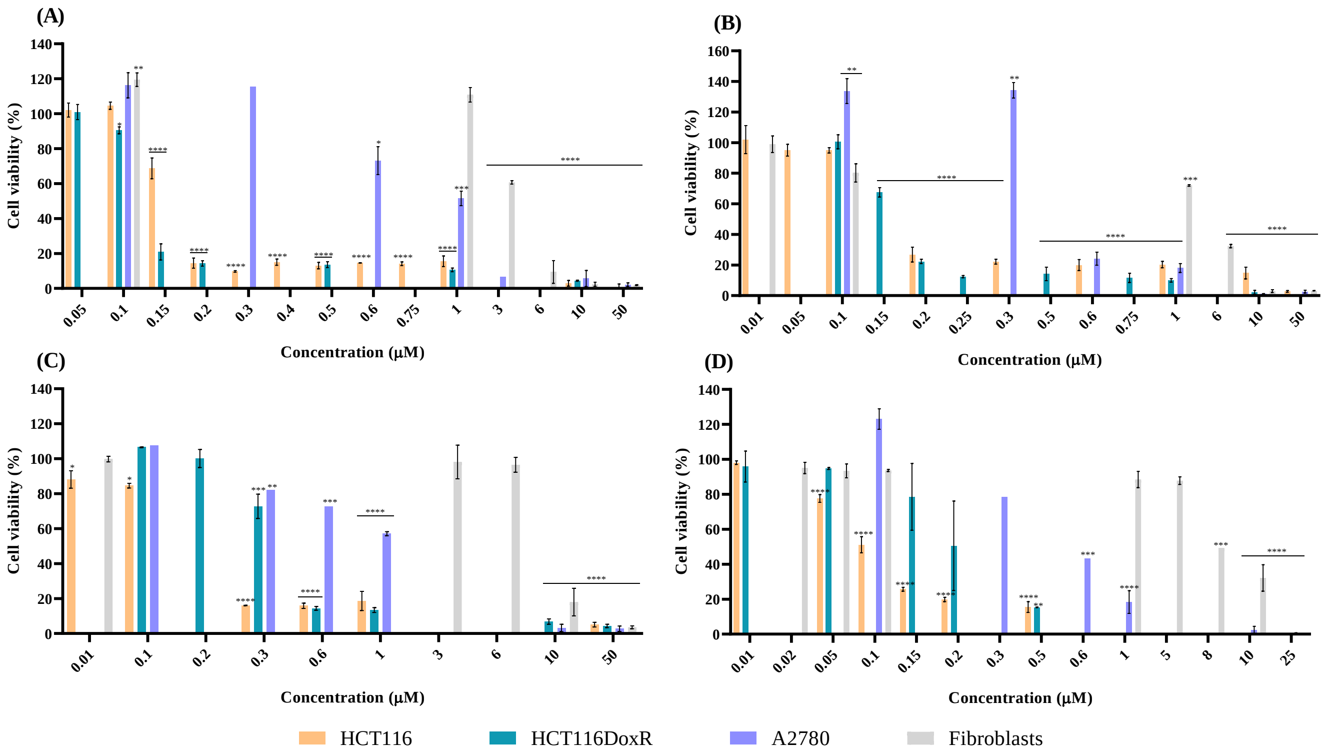

Figure S11. Cell viability of HCT116, HCT116DoxR, and A2780 tumor cell lines and primary normal fibroblasts after exposure to different concentrations of copper complexes **1 (A)**, **4 (B)**, **6 (C)**, and **8 (D)** for 48 h. DMSO in the same % as in the complexes was used as the vehicle control. Data are expressed as the mean ± SEM of at least two biological independent assays. Statistical significance was assessed relative to control (DMSO) by the one-way ANOVA method (* $p < 0.05$; ** $p < 0.01$; *** $p < 0.001$; **** $p < 0.0001$).

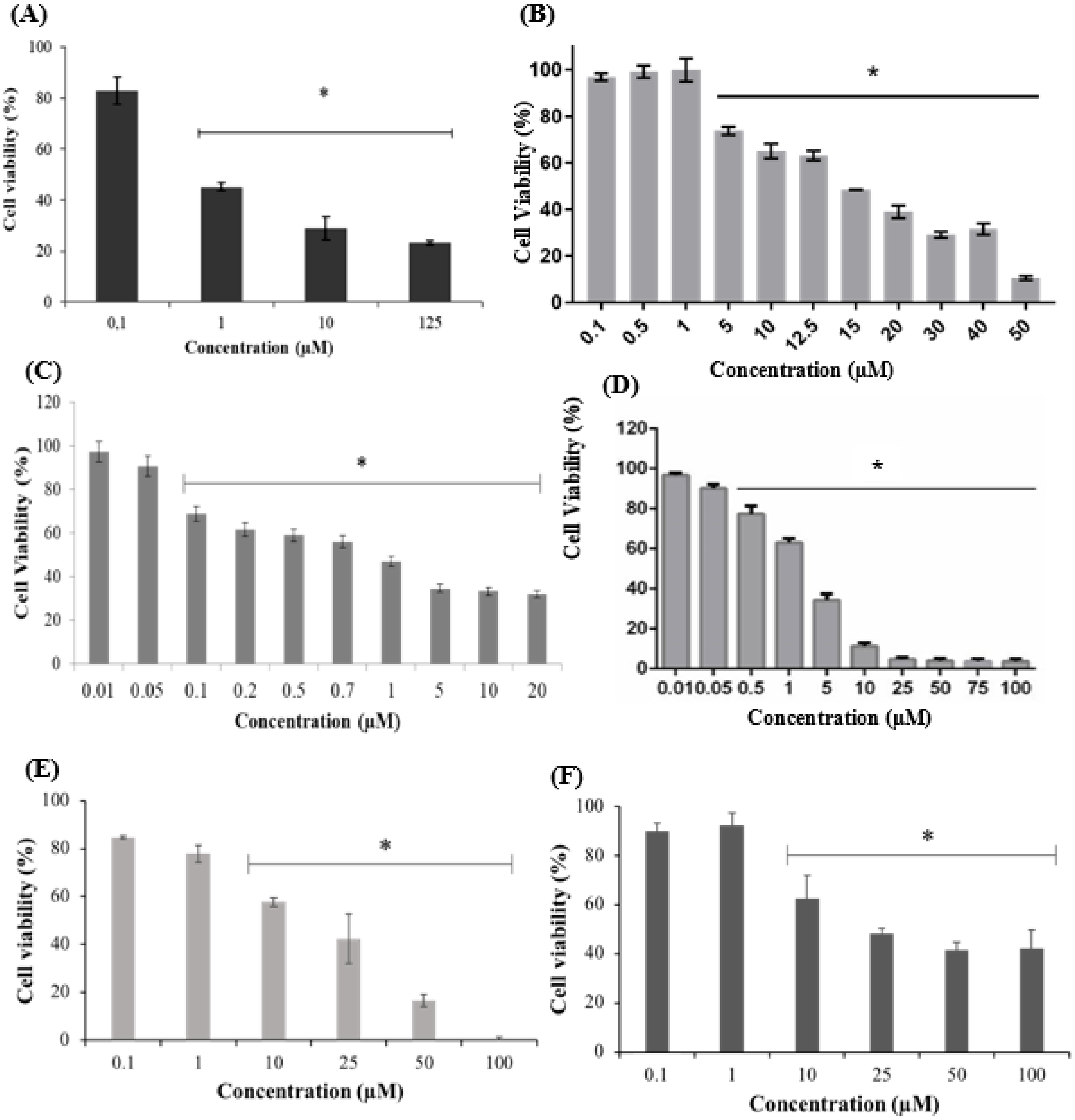

Figure S12. Viability of HCT116 cancer cell line after exposure to different concentrations of doxorubicin **(A)** and cisplatin **(B)** for 48 h. Viability of A2780 cancer cell line after exposure to different concentrations of doxorubicin **(C)** and cisplatin **(D)** for 48 h. Viability of Fibroblasts after exposure to different concentrations of doxorubicin **(E)** and cisplatin **(F)** for 48 h. 0.1% (v/v) DMSO was used as the vehicle control. Data are expressed as the mean ± SEM of three biological assays.

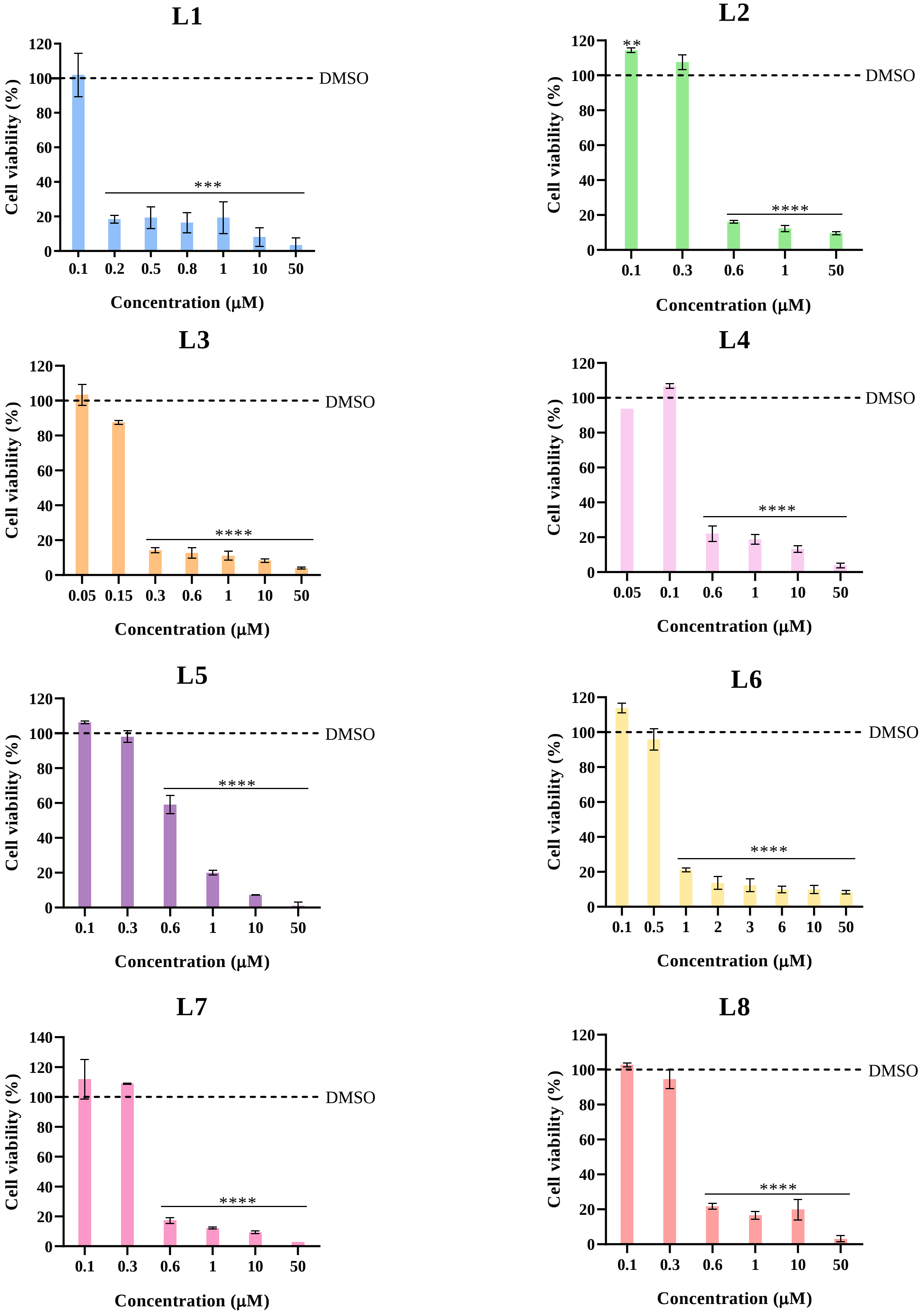

Figure S13. Cell viability of HCT116DoxR tumor cell line after exposure to different concentrations of ligands **L1-L8** for 48 h. DMSO in the same % as in the ligands was used as the vehicle control. Data are expressed as the mean ± SEM of at least two biological independent assays. Statistical significance was assessed relative to control (DMSO) by the one-way ANOVA method (*** $p < 0.001$; **** $p < 0.0001$).

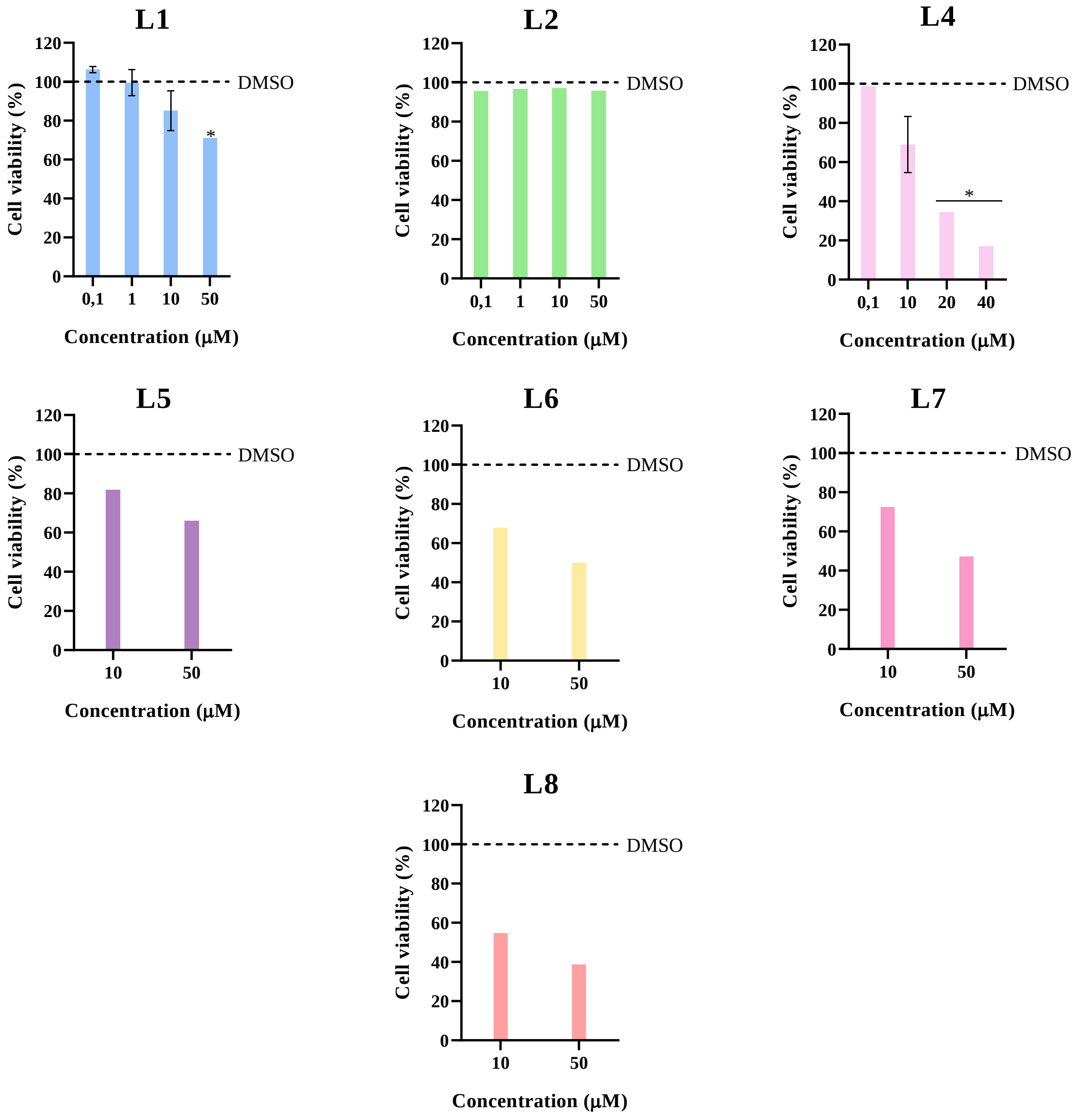

Figure S14. Cell viability of fibroblasts after exposure to different concentrations of ligands **L1-L8** for 48 h. DMSO in the same % as in the ligands was used as vehicle control. Data from L1 and L4 are expressed as the mean ± SEM of at least two biological independent assays. Statistical significance was assessed relative to control (DMSO) by the one-way ANOVA method (* $p < 0.05$).

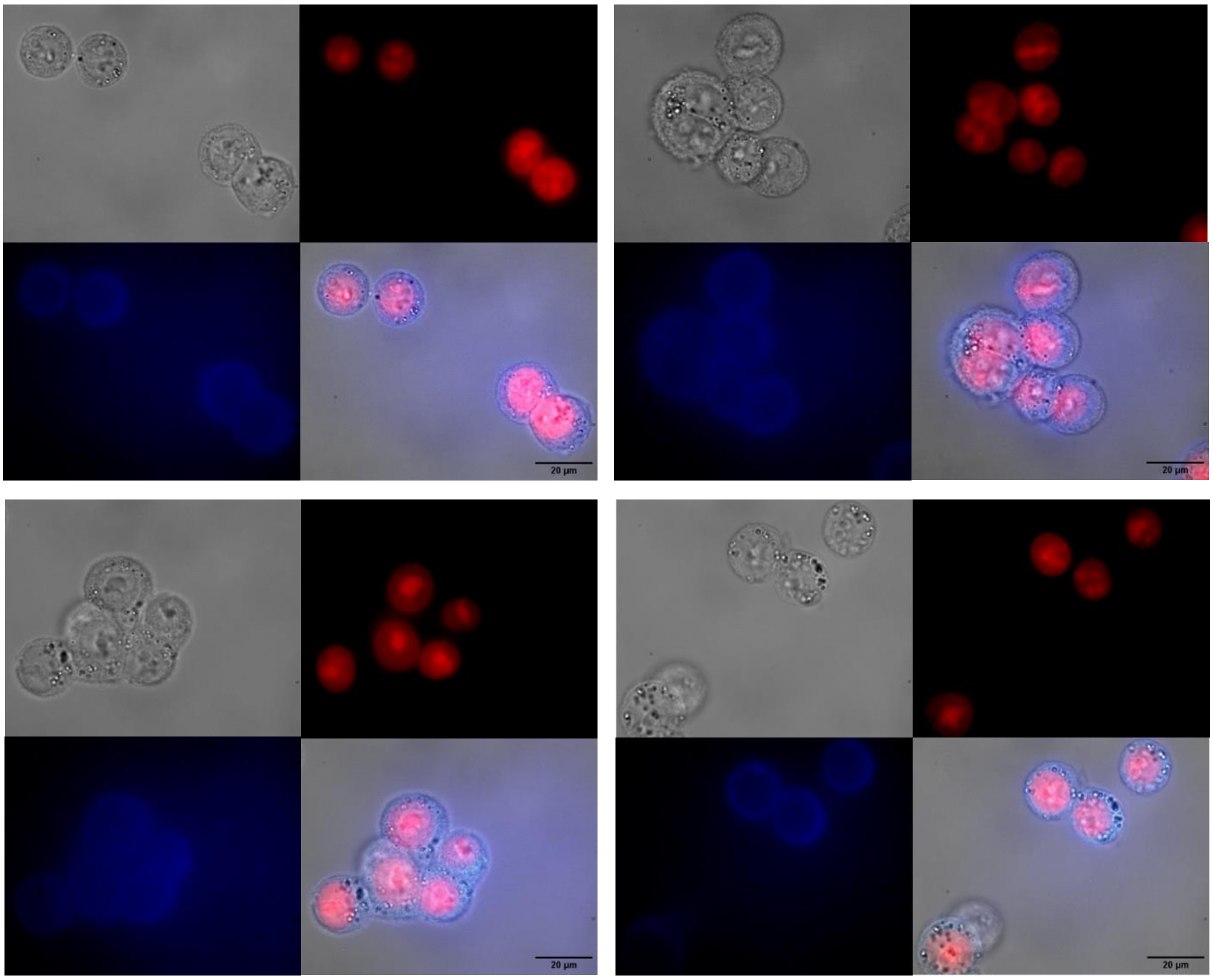

Figure S15. Fluorescence microscopy of HCT116DoxR cells incubated for 3 h with 10x the IC_{50} of complex **2** (blue). The nuclei were labelled with PI (red). Complex **2** presents a maximum excitation at 290 nm in the UV region and its maximum emission at 410-420 nm in the blue region.

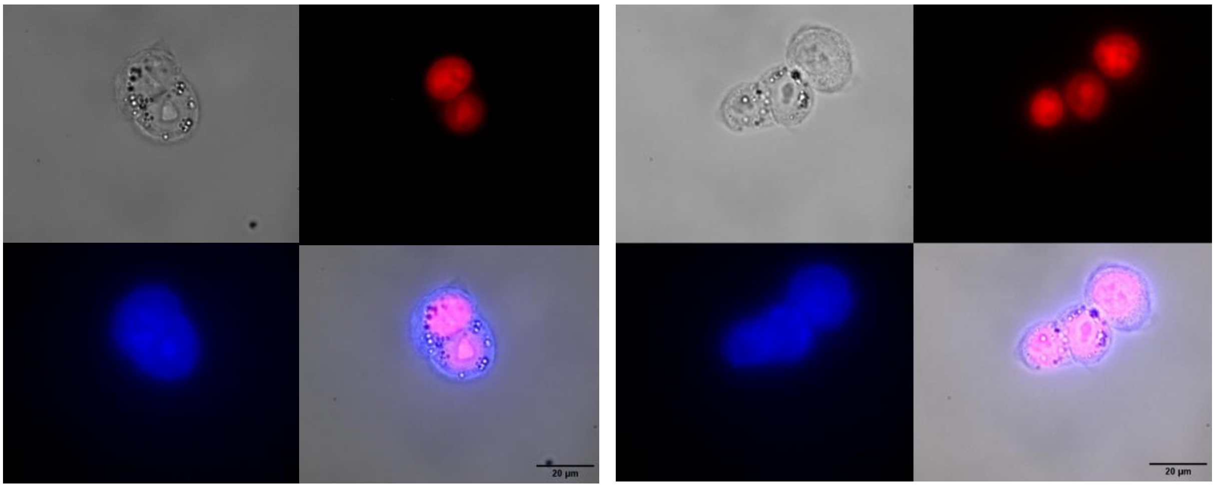

Figure S16. Fluorescence microscopy of HCT116DoxR cells incubated for 3 h with 10x the IC_{50} of complex **3** (blue). The nuclei were labelled with PI (red). Complex **3** presents a maximum excitation at 290 nm in the UV region and its maximum emission at 410-420 nm in the blue region.

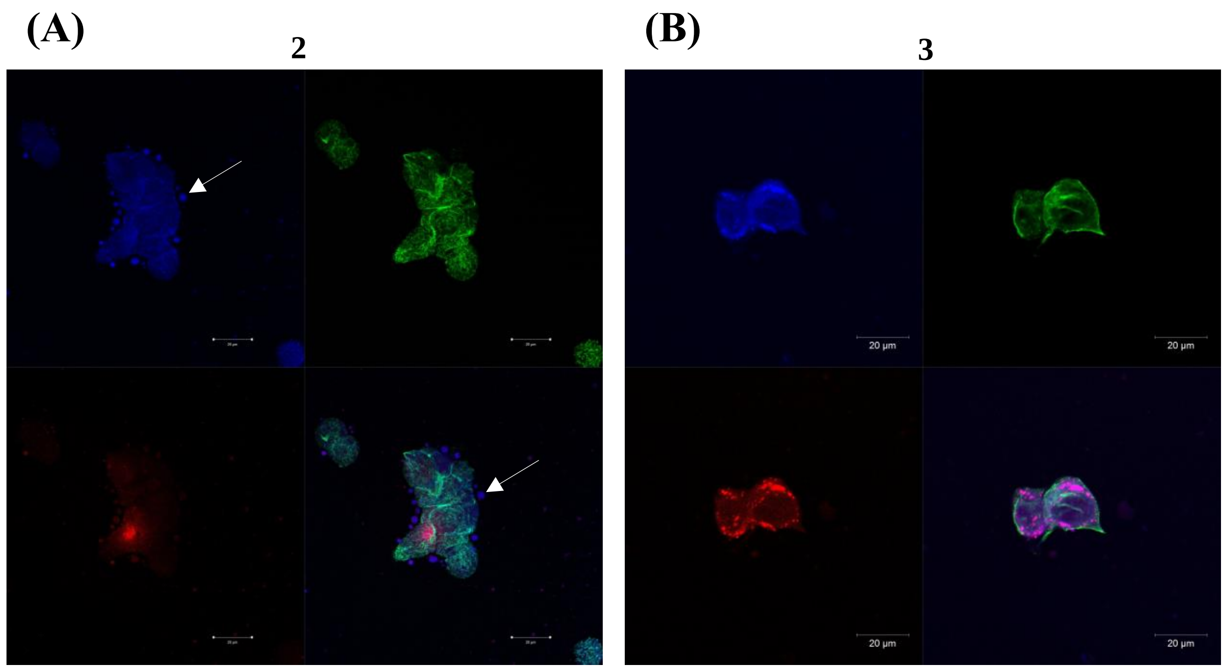

Figure S17. Confocal microscopy of HCT116DoxR cells incubated for 3 h with 10x the IC_{50} concentrations of complexes **2 (A)** and **3 (B)**. The nuclei were labelled with PI (red), the actin cytoskeleton was labelled with Alexa Fluor^{TM} 488 Phalloidin (green), while the fluorescent complexes are in blue. The white arrow indicates complex **2** aggregates in the vicinity of the cells. The complexes present a maximum excitation at 290 nm in the UV region and their maximum emission at 410-420 nm in the blue region.

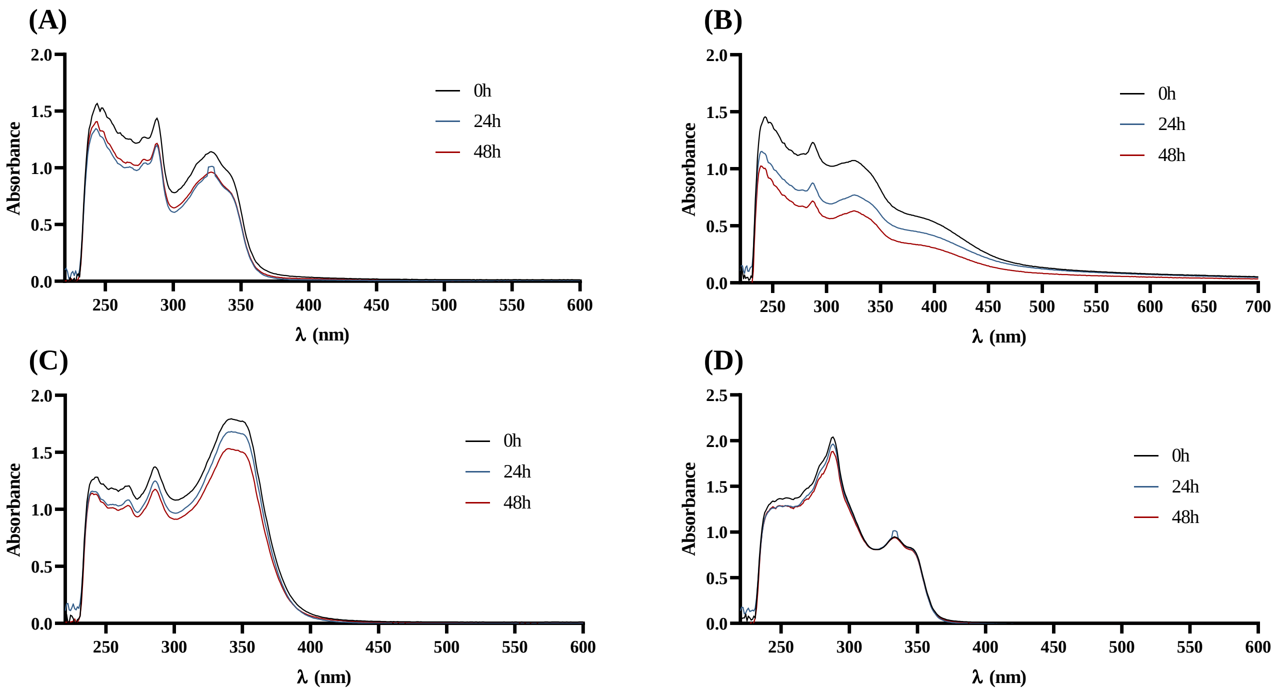

Figure S18. Evaluation of the stability and solubility of complexes **2 (A)**, **3 (B)**, **5 (C)**, and **7 (D)** by UV-Visible spectroscopy over 48 h. The complexes were dissolved in DMSO and then diluted in colourless RPMI medium (without *phenol red*) with 0% FBS to a final concentration of 50 µM. The results presented were normalized with respect to the 1% (v/v) DMSO control.

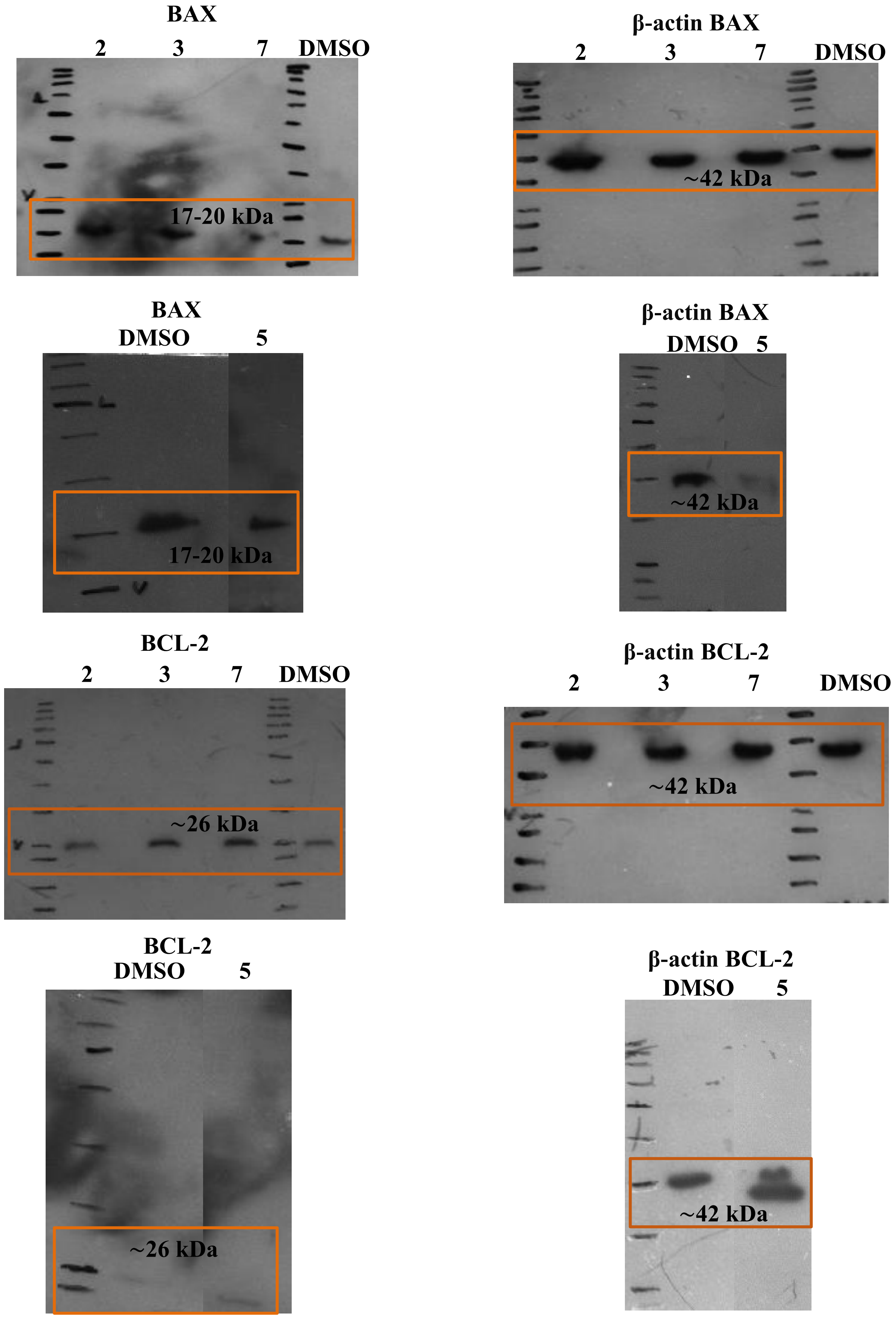

Figure S19. Western blot bands used to quantify BAX and BCL-2 proteins in HCT116DoxR cells after 48 h exposure to the complexes **2**, **3**, **5**, and **7** or 0.1% (v/v) DMSO.

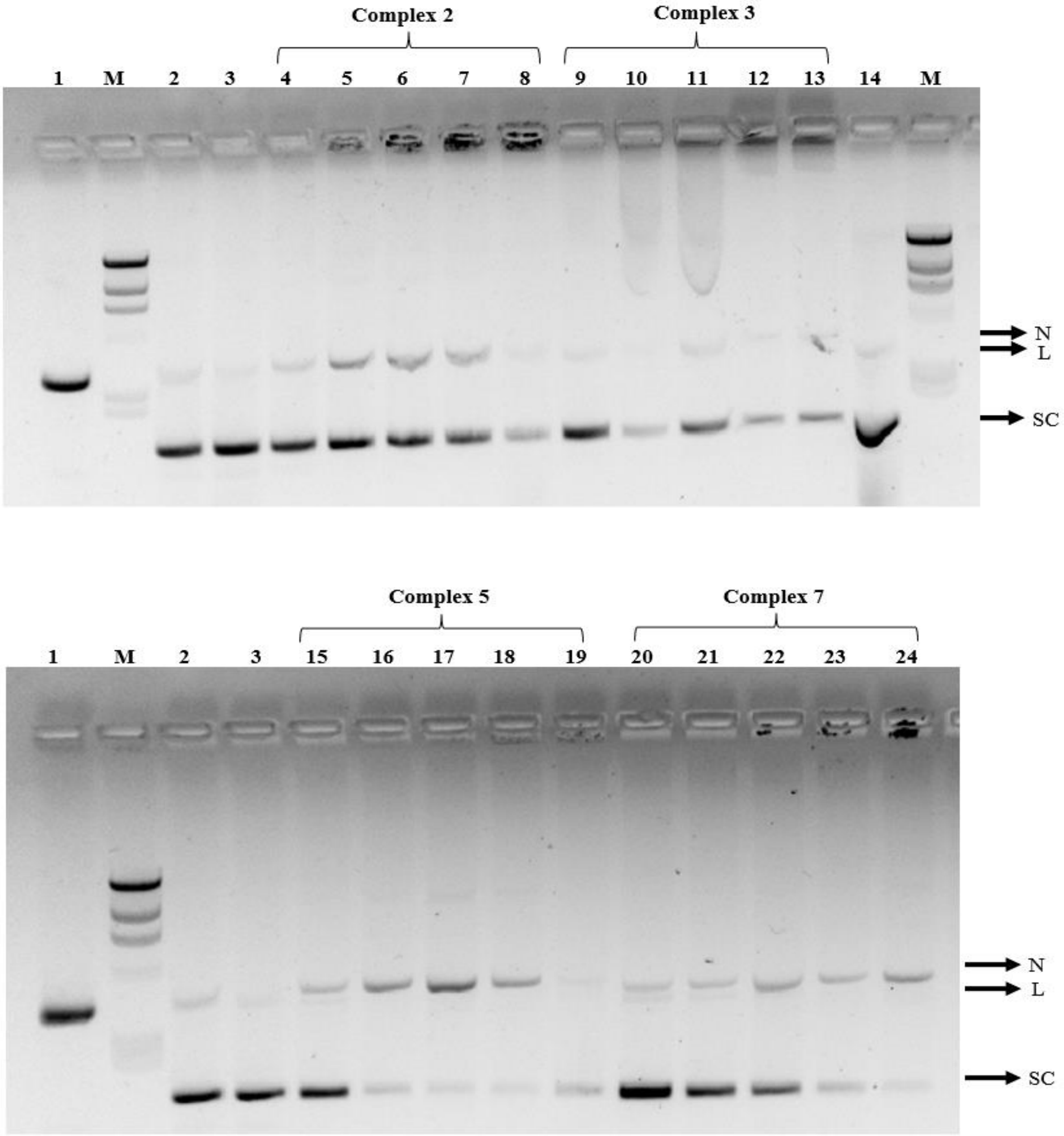

Figure S20. Electrophoresis in 0.8% (w/v) agarose gel to analyse the electrophoretic mobility of the pDNA samples after 24 h of exposure to increasing concentrations of complexes **2**, **3**, **5**, and **7**. Conditions: TAE 1x; Gel Red 0.0015% (v/v); 70 V, 80 min. 1 - pUC18 incubated with HindIII for 2 h; M - HindIII lambda molecular weight marker; 2 - pUC18 exposed to 5 mM Tris-HCl and 50 mM NaCl pH=7.0 buffer solution and 1% (v/v) DMSO for 24h; 3 - pUC18 exposed to 5 mM Tris-HCl and 50 mM NaCl pH=7.0 buffer solution for 24 h; 4, 9, 15, 20 - pUC18 exposed to 5 μM of the indicated complex for 24 h; 5, 10, 16, 21 - pUC18 exposed to 25 μM of the indicated complex for 24 h; 6, 11, 17, 22 - pUC18 exposed to 50 μM of the indicated complex for 24 h; 7, 12, 18, 23 - pUC18 exposed to 75 μM of the indicated complex for 24 h; 8, 13, 19, 24 - pUC18 exposed to 100 μM of the indicated complex for 24 h; 14 - pUC18. The arrows correspond to the isoforms of the pUC18 plasmid (N - nicked; L - linear; SC - supercoiled).

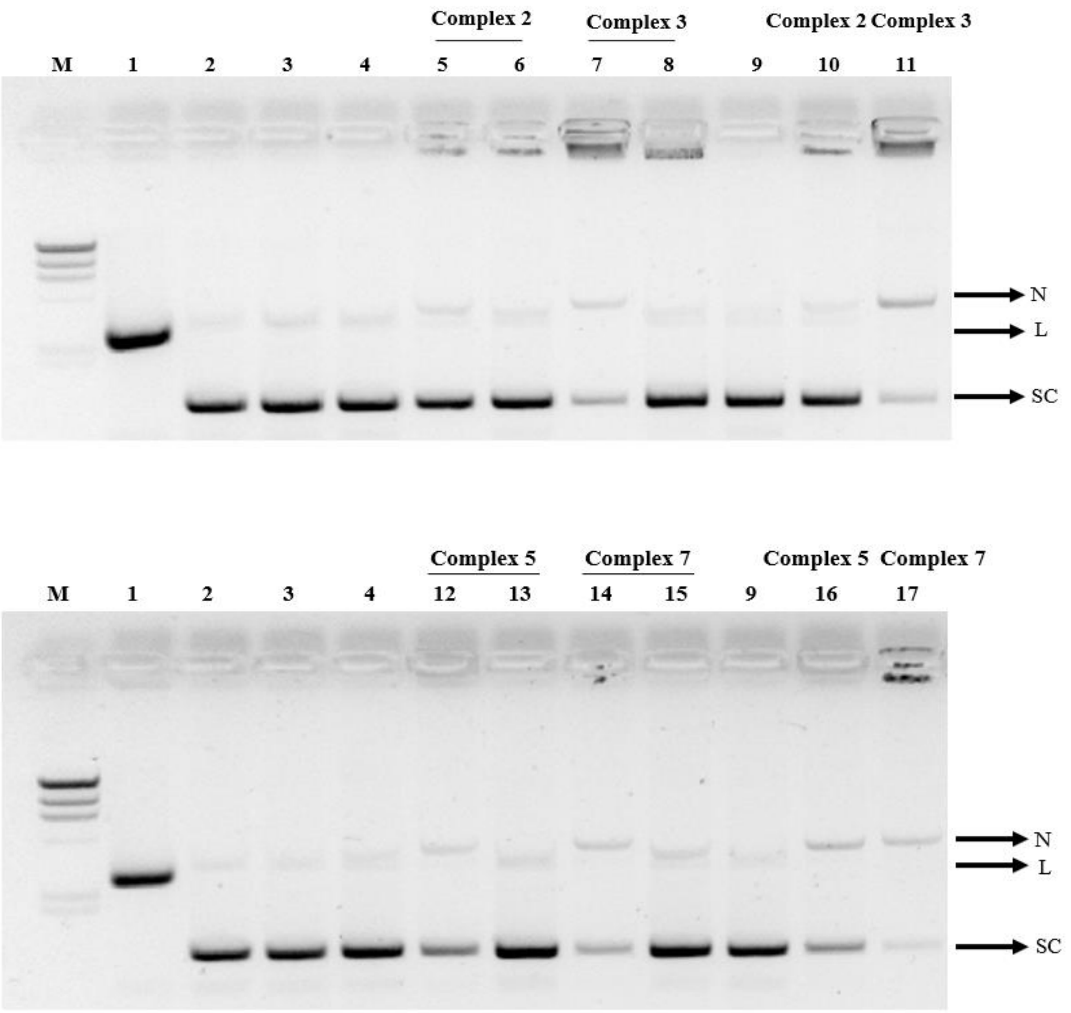

Figure S21. Electrophoresis in 1% (w/v) agarose gel to determine the mechanisms of pDNA cleavage by complexes **2, 3, 5,** and **7**. Conditions: TAE 1x; Gel Red 0.0015% (v/v); 70 V, 80 min. M - HindIII lambda molecular weight marker; 1 - pUC18 incubated with HindIII for 2 h; 2 - pUC18 exposed to 5 mM Tris-HCl and 50 mM NaCl pH=7.0 buffer solution and 1% (v/v) DMSO for 24 h; 3 - pUC18 exposed to 5 mM Tris-HCl and 50 mM NaCl pH=7.0 buffer solution for 24 h; 4 - pUC18 exposed to 50 µM NaN3 for 24 h; 5, 7, 12, 14 - pUC18 exposed to 50 µM of the indicated complex for 24 h; 6, 8, 13, 15 - pUC18 exposed to 50 µM of NaN3 and 50 µM of the indicated complex for 24 h; 9 - pUC18 exposed to 20 µM of TBHP for 24 h; 10, 11, 16, 17 - pUC18 exposed to 20 µM of TBHP and 50 µM of the indicated complex for 24 h. The arrows correspond to the isoforms of the pUC18 plasmid (N - nicked; L - linear; SC - supercoiled).

References:

(1) Sheldrick, G. M. A Short History of SHELX. *Acta Crystallogr. A* **2008**, *64* (1), 112–122. https://doi.org/10.1107/S0108767307043930.

(2) Sheldrick, G. M. Crystal Structure Refinement with SHELXL. *Acta Crystallogr. Sect. C Struct. Chem.* **2015**, *71* (1), 3–8. https://doi.org/10.1107/S2053229614024218.

(3) Sheldrick, G. M. SHELXT – Integrated Space-Group and Crystal-Structure Determination. *Acta Crystallogr. Sect. Found. Adv.* **2015**, *71* (1), 3–8. https://doi.org/10.1107/S2053273314026370.

(4) Pedrosa, P.; Mendes, R.; Cabral, R.; Martins, L. M. D. R. S.; Baptista, P. V.; Fernandes, A. R. Combination of Chemotherapy and Au-Nanoparticle Photothermy in the Visible Light to Tackle Doxorubicin Resistance in Cancer Cells. *Sci. Rep.* **2018**, *8* (1). https://doi.org/10.1038/s41598-018-29870-0.

(5) Stockert, J. C.; Horobin, R. W.; Colombo, L. L.; Blázquez-Castro, A. Tetrazolium Salts and Formazan Products in Cell Biology: Viability Assessment, Fluorescence Imaging, and Labeling Perspectives. *Acta Histochem.* **2018**, *120* (3), 159–167. https://doi.org/10.1016/j.acthis.2018.02.005.

(6) Valente R, Cordeiro S, Luz A, Melo MC, Rodrigues CR, Baptista PV, Fernandes AR. Doxorubicin-sensitive and -resistant colorectal cancer spheroid models: assessing tumor microenvironment features for therapeutic modulation. *Front Cell Dev Biol.* **2023**, *11,* 1310397. https://doi.org/10.3389/fcell.2023.1310397.

(7) Luís, D. V.; Silva, J.; Tomaz, A. I.; De Almeida, R. F. M.; Larguinho, M.; Baptista, P. V.; Martins, L. M. D. R. S.; Silva, T. F. S.; Borralho, P. M.; Rodrigues, C. M. P.; Rodrigues, A. S.; Pombeiro, A. J. L.; Fernandes, A. R. Insights into the Mechanisms Underlying the Antiproliferative Potential of a Co(II) Coordination Compound Bearing 1,10-Phenanthroline-5,6-Dione: DNA and Protein Interaction Studies. *J. Biol. Inorg. Chem.* **2014**, *19* (6), 787–803. https://doi.org/10.1007/s00775-014-1110-0.

(8) Jahanban-Esfahlan, A.; Panahi-Azar, V.; Sajedi, S. Spectroscopic and Molecular Docking Studies on the Interaction between *N* -acetyl Cysteine and Bovine Serum Albumin. *Biopolymers* **2015**, *103* (11), 638–645. https://doi.org/10.1002/bip.22697.

(9) Reichmann, M. E.; Rice, S. A.; Thomas, C. A.; Doty, P. A Further Examination of the Molecular Weight and Size of Desoxypentose Nucleic Acid. *J. Am. Chem. Soc.* **1954**, *76* (11), 3047–3053. https://doi.org/10.1021/ja01640a067.

(10) Marmur, J. A Procedure for the Isolation of Deoxyribonucleic Acid from Micro-Organisms. *J. Mol. Biol.* **1961**, *3* (2), 208-IN1. https://doi.org/10.1016/S0022-2836(61)80047-8.

(11) Choroba, K.; Machura, B.; Szlapa-Kula, A.; Malecki, J. G.; Raposo, L.; Roma-Rodrigues, C.; Cordeiro, S.; Baptista, P. V.; Fernandes, A. R. Square Planar Au(III), Pt(II) and Cu(II) Complexes with Quinoline-Substituted 2,2′:6′,2″-Terpyridine Ligands: From in Vitro to in Vivo Biological Properties. *Eur. J. Med. Chem.* **2021**, *218*, 113404. https://doi.org/10.1016/j.ejmech.2021.113404.

(12) Reigosa-Chamorro, F.; Raposo, L. R.; Munín-Cruz, P.; Pereira, M. T.; Roma-Rodrigues, C.; Baptista, P. V.; Fernandes, A. R.; Vila, J. M. In Vitro and in Vivo Effect of Palladacycles: Targeting A2780 Ovarian Carcinoma Cells and Modulation of Angiogenesis. *Inorg. Chem.* **2021**, *60* (6), 3939–3951. https://doi.org/10.1021/acs.inorgchem.0c03763.
